# Hydroboration-Oxidation of (±)-(1α,3α,3aβ,6aβ)-1,2,3,3*a*,4,6*a*-Hexahydro-1,3-pentalenedimethanol and Its *O*-Protected Derivatives: Synthesis of New Compounds Useful for Obtaining (*iso*)Carbacyclin Analogues and X-ray Analysis of the Products

**DOI:** 10.3390/molecules22122032

**Published:** 2017-11-24

**Authors:** Constantin I. Tănase, Florea G. Cocu, Miron Teodor Căproiu, Constantin Drăghici, Sergiu Shova

**Affiliations:** 1 National Institute for Chemical-Pharmaceutical Research and Development, Department of bioactive substances and pharmaceutical technologies, 112 Vitan Av., 031299, Bucharest-3, Romania; fgcocu@gmail.com; 2 Organic Chemistry Center “C.D.Nenitescu”, Spectroscopy Laboratory, 202 B Splaiul Independentei, 060023 Bucharest, Romania; dorucaproiu@gmail.com (M.T.C.); cst_drag@yahoo.com (C.D.); 3 Institute of Macromolecular Chemistry “Petru Poni”, Crystallography Department, 700478 Iasi, Romania; shova@icmpp.ro

**Keywords:** hydroboration-oxidation, borane, sodium acetoxyborohydride, hexahydro-1,3-pentalenedimethanol, octahydropentalene-triols, octahydropentalene-ketones, X-ray analysis

## Abstract

Hydroboration-oxidation of 2α,4α-dimethanol-1β,5β-bicyclo[3.3.0]oct-6-en dibenzoate (**1**) gave alcohols **2** (symmetric) and **3** (unsymmetric) in ~60% yield, together with the monobenzoate diol **4a** (37%), resulting from the reduction of the closer benzoate by the intermediate alkylborane. The corresponding alkene and dialdehyde gave only the triols **8** and **9** in ~1:1 ratio. By increasing the reaction time and the temperature, the isomerization of alkylboranes favours the un-symmetrical triol **9**. The PDC oxidation of the alcohols gave cleanly the corresponding ketones **5** and **6** and the deprotection of the benzoate groups gave the symmetrical ketone **14**, and the cyclic hemiketal **15**, all in high yields. The ethylene ketals of the symmetrical ketones **11** and **13** were also obtained. The compounds **5**, **6**, **11**, **13**, **14** could be used for synthesis of new (iso)carbacyclin analogues. The structure of the compounds was established by NMR spectroscopy and confirmed by X-ray crystallography.

## 1. Introduction

**Figure 1:**
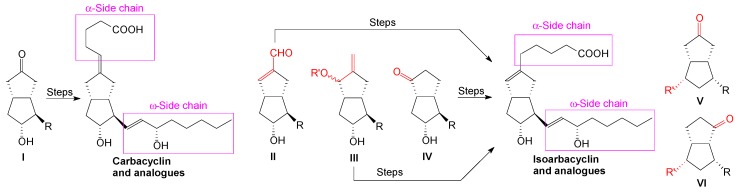
Key intermediates for synthesis of carbacyclin and isocarbacyclin analogues.

The synthesis of hydrogenated pentalene ketone intermediates has found applications in obtaining new drugs, of which the best known are carbacyclin [1] and isocarbacyclin [2,3,4,5,6,7,8] analogues, cioctol [9] and a few antibacterial [10] and antitumor natural products [11,12]. In carbacyclin synthesis, the standard key intermediate **I** has a symmetric ketone for linking the α-side chain by a Wittig *E*-olefination (at the ω-side chain) and a R group which finally is transformed into an aldehyde used to introduce the ω-side chain by a stereoselective Wardworth-Emmons *E*-olefination with a phosphonate (Figure 1) [13,14,15,16].

In the synthesis of isocarbacyclin analogues, a few pentalene intermediates with different structures have been used to build the α-side chain (Figure 1). The type **II** intermediate contains an exocyclic allylic aldehyde for building the α-side chain [17], while the type **III** intermediate has an allylic alcohol or ester for introducing the α-side chain by a regioselective SN2’ alkylation with zinc-copper organic reagents [18,19,20], and the unsymmetric ketone intermediate of type **IV** links the α-side chain by different methods [21,22,23,24].

The synthesis of new carbacyclin and isocarbacyclin analogs requires new key intermediate octahydropentalene ketones. In this paper, we describe the synthesis of the ketone-octahydropentalenes of types **V** and **VI** useful for obtaining new carbacyclin and isocarbacyclin analogues.

## 2. Results and Discussion

**Scheme 1:**
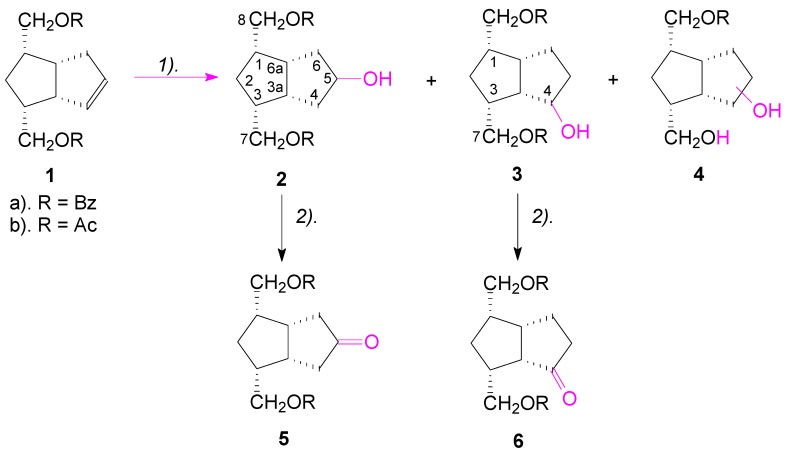
Hydroboration-oxidation of the compounds **1** for obtaining the octahydro-pentalenofurane ketones **5** and **6**. Reagents and Conditions: (*1*) (a) Sodium acetoxyborohydride or BH_3_·THF, r.t, 24 h; (b) 30% H_2_O_2_/3 M AcONa or 3 M NaOH, 5 °C, ~35 min. (*2*) PDC/molecular sieves, r.t., overnight.

Retrosynthetic analysis of the key intermediates **5** and **6** (Scheme 1), indicates that these compounds could be obtained by a sequence of two reactions: hydration of the double bond of alkene **1** to an alcohol, followed by the oxidation of the alcohol to the corresponding ketone. The starting compounds **1** were previously synthesized [25] and have already been used for the synthesis of “pseudocarbacyclin” type compounds [26,27], of the corresponding diols by hydroxylation with KMnO_4_ [28], of the corresponding α and β-epoxides, by epoxidation of the double bond with MPBA [29], and of pentalenofuranic compounds by regioselective reactions [30]. Following our retrosynthetic analysis, we decided to hydrate the double bond of alkene **1** by hydroboration-oxidation with sodium acetoxyborohydride and with borane.

**Scheme 2:**
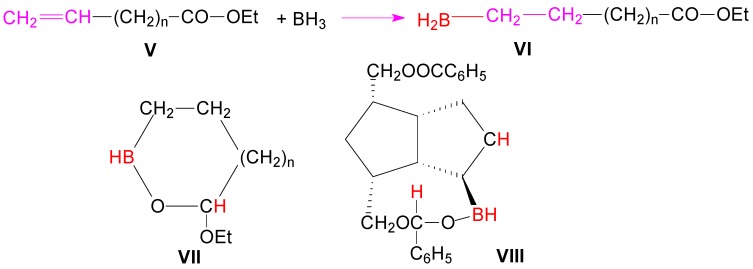
Hydroboration mechanism through a cyclic borane ester.

Hydroboration-oxidation of alkene **1a** with sodium acetoxyborohydride [31] resulted in the formation, in a non-regioselective manner, of both alcohols **2a** (34%) and **3a** (25%), slightly in favor of the symmetrical alcohol **2**. In the reaction, the monobenzoate-triol **4a** was also formed in a great quantity (38%) (Scheme 1). Browsing the literature, we found that in the case of the unsaturated esters **V**, by forming a cyclic intermediate with 5 or 6 atoms **VII**, hydroboration of the double bond proceeded with concomitant reduction of the ester group (Scheme 2) [32].

Probably it is the same formation of a similar cyclic ester **VIII** in the hydroboration of diester compound **1**, which favored the reduction of the closer ester group to double bond, this reduction being responsible for the formation of the monoacylated triols **4a** and **4b**.

**Figure 2:**
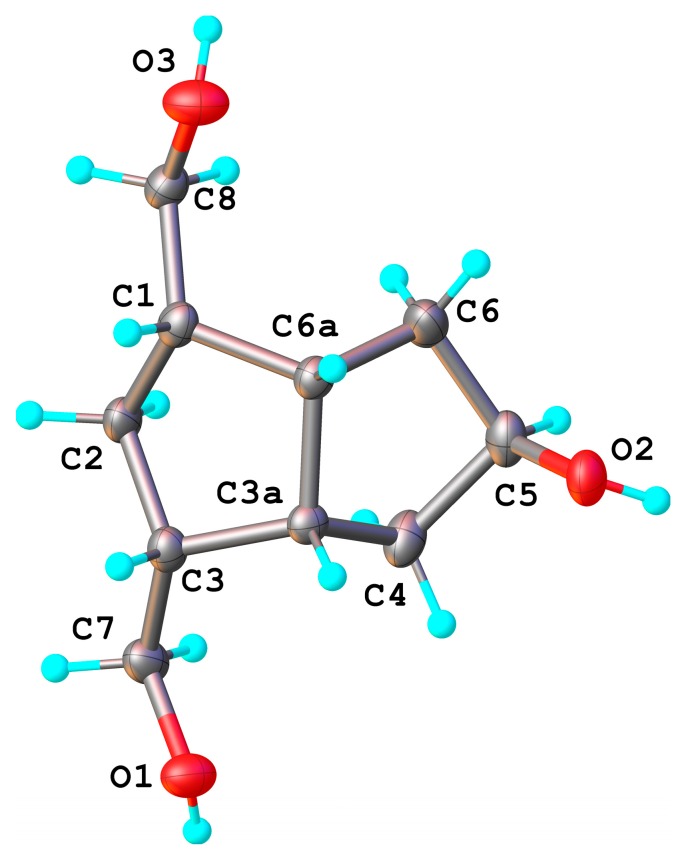
X-ray molecular structure of the symmetrical triol compound **8** with thermal ellipsoids at 50% probability level. Proposed atom numbering is shown in the scheme attached and will be kept the same for all compounds.

In the hydroboration-oxidation of diacetate **1b**, the monoacetate-triol **4b** was formed also in 38% yield, and this is consistent with the mechanism presented in Figure 2 for the formation of monoesters **4**; the corresponding alcohols **2b** and **3b** were formed in about 1:1 ratio, but their isolation in pure form by low-pressure chromatography (LPC) was more difficult than in the case of the benzoate compounds **2a** and **2b**.

The hydroboration of **1** with BH_3_·THF, obtained in situ from NaBH_4_ and dimethyl sulfate, followed by H_2_O_2_ oxidation gave alcohols **2a**, **3a** and **4a** in 31.5%, 28.8% and 34.5% yield; hence, there is no significant difference in the yields and the ratio of the alcohols between the hydroboration with NaBH_3_OOCCH_3_ and BH_3_. It is worth mentioning that the isolation of the alcohols by PC is easier for the benzoate esters than the acetate esters. The formation of the unwanted secondary by-products **4a** and **4b** could represent also an advantage, because it is easy to selectively protect the primary hydroxyl group, with a group different from benzoate, like an ether, trityl, *tert*-butyldimethylsilyl or other bulky silyl-protecting group, by the methods known in the art, and thus to obtain different protection of the hydroxymethyl groups, useful for the next steps.

The fact that by-product **4** has the hydroxyl group linked at the C_4_ atom (see below) means that the hydroboration-oxidation of benzoate **1a** gave the C_4_-alcohol in a total yield of 62–63%.

**Scheme 3:**
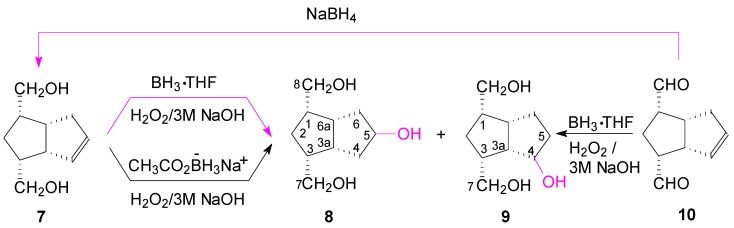
Hydroboration-oxidation of alkene-diol **7** with borane and sodium acetoxyborohydride and dialdehyde **10** with borane.

We then used the hydroboration-oxidation on the diol **7** (Scheme 3). We observed that hydroboration with borane is still a slow reaction and a certain amount of alkene **7** remains unreacted. We used different molar ratios of BH_3_·THF/**7**, from 1.2:1 to 4:1, and the results are presented in Table 1.

At a ratio of 2:1 BH_3_·THF/**7**, the borane reacted with the hydroxymethyl groups forming two alcoxyborane groups. The alkoxyborane group, closer to the double bond, hydroborated at the nearest carbon atom (C-4) with the formation in excess of the un-symmetrical alcohol **9**, through an intermediate of type **VIII** (Scheme 2); the yield of alcohols **8** and **9** was still low (40.8%). By increasing the ratio of BH_3_·THF/**7** to 3:1 and 4:1, there remained free borane which increased the yield of alcohols to 64.4%, and respectively to 73.3%, but there was no selectivity against **9** and the ratio of alcohols was nearly 1:1 (**8**/**9**).

**Scheme 4:**
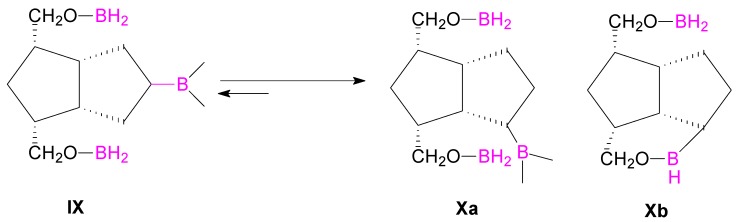
Isomerization of alkyl-boranes **IX** to **X**.

Finally, we performed the hydroboration-oxidation of the double bond, concomitant with the reduction of the aldehyde groups of dialdehyde **10** (from which we previously [25] obtained the alkene-diol **7** by NaBH_4_ reduction of the aldehyde groups) with 2.2 molar equivalents of BH_3_·THF (20 h), and a mixture of alkene **7**, alcohols **9** and **8** was obtained in a ratio of 1.0:1.1:1.2. When the hydroboration was done with a greater molar ratio BH_3_·THF:**7** of 3:1 (preparative scale on 0.359 M alkene **7**) and time was increased to 72 h, the ratio of alcohols (**8**/**9**) was 1.0:1.2. Then another reaction was performed for 24 h at r.t. and for 2 h at 45–50 °C and the ratio of the alcohols changed to ~1.0:2.2 (**8**/**9**). These suggest that an isomerization of the alkyl-boranes took place from type **IX** to **Xa** or **Xb** (Scheme 4), more thermodinamically stable in the reaction conditions.

Such isomerizations between alkyl-boranes are known in the literature [33,34,35,36,37]. The α- or β-configuration of the hydroxyl groups introduced is not important, because the OH is oxidized in the next step to a ketone. Nonetheless, we believe that the secondary hydroxyl group is mainly introduced in a β orientation, because the access of the hydroboration reagent to the double bond ocurrs from the *exo*-side (the bulky crystallized compounds were only analyzed; the compound(s) remaining in the mother liquors were not analyzed for α/β isomers). The H_5_ appears in ^1^H-NMR as a broad singlet and is not a clear evidence for the α-configuration, but the X-ray diffraction analysis (Figure 2) of the crystallized triol **8** confirmed the *exo*-configuration of the secondary alcohol linked to the C_5_ position, 5β-OH. Bond distances and angles for compound **8** are listed in Table S1 and crystallographic data, details of data collection and structure refinement parameters in Table S2 (also for compounds **3**, **5** and **14**, Supplementary Material).

**Figure 3:**
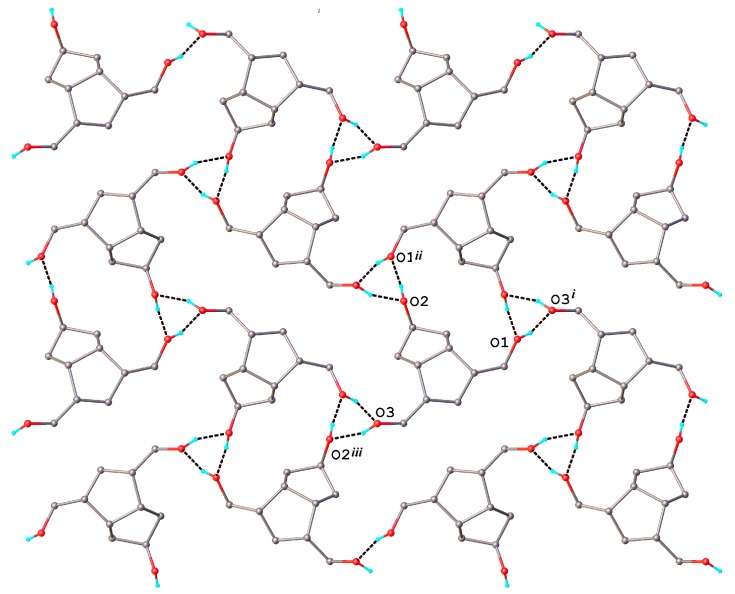
Three-dimensional supramolecular architecture in the crystal structure **8**.

The compound **8** exhibits a molecular crystal structure where the neutral molecules interact through O-H···O hydrogen bonding to form a three-dimensional supramolecular network, as shown in Figure 3. H-bonding parameters are listed in Table 2.

**Figure 4:**
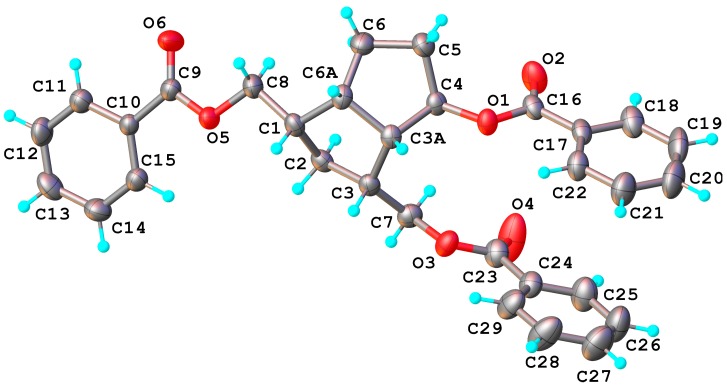
X-ray molecular structure of non-symmetrical triol tribenzoate compound **3** with atom labeling scheme and thermal ellipsoids at 50% probability level.

The configuration of the 4-OH in **9** was not studied, since the compound was obtained as an oil. For confirming the structure of the unsymmetrical alcohols (OH linked to C_4_), we synthesized the tri-benzoates of **3** and **4** (and also of **2**) to obtain suitable crystals for X-ray analysis, and their preparation is presented in the paper; their characterization by NMR was also done. At least for the fractions used for the analysis of the trisbenzoate obtained from **3**, X-ray crystallography confirmed that the secondary 4-OH group are linked *exo* (β) to the carbon atom, as in the case of **8**. The result of single crystal X-ray diffraction study for this compound is shown in Figure 4, while bond distances and angles are summarized in Table S1.

Thus, the hydroboration-oxidation of alcohol **7** or dialdehyde **10** gives the symmetrical alcohol **8** in about 38% yield, but by increasing the reaction time and using hydroboration at elevated temperatures, the unsymmetrical alcohol **9** is formed in excess due to isomerization of the intermediary alkylboranes.

**Scheme 5:**
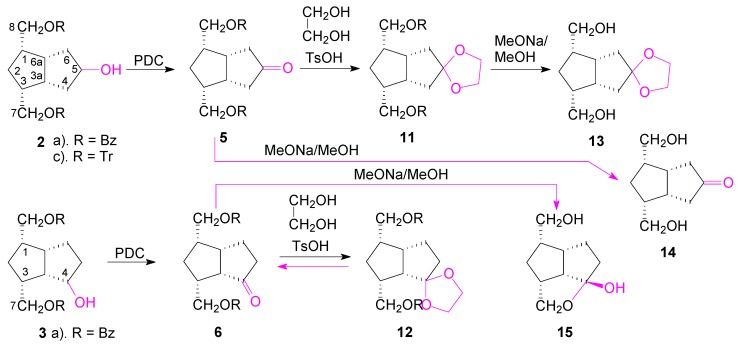
Synthesis of carbonyl compounds from alcohols **2** and **3**.

The regioisomers **8** (crystallized, m.p. 98–99 °C) and **9** (oil) were separated by LPC; their use in the next steps requires the selective protection of the primary hydroxyl groups, as exemplified for the obtaining of **2c** (R = Tr) by treating **8** with trityl chloride; the following sequence is similar to the one of **2a** (Scheme 5).

**Figure 5:**
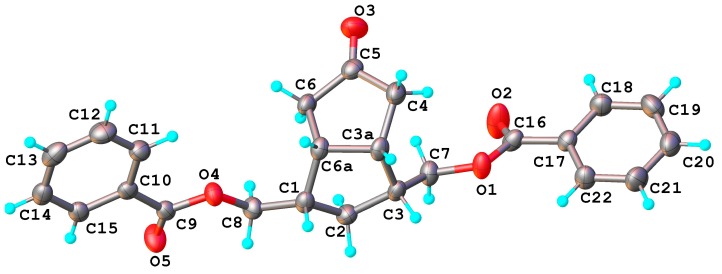
X-ray molecular structure of symmetric ketone bis-benzoate compound **5** with atom labeling scheme and thermal ellipsoids at 50% probability level.

The secondary alcohols of **2** and **3** were oxidized with PDC to the corresponding ketones **5** and **6** and then the benzoate protecting groups were cleanly removed by transesterification with MeONa in MeOH (Scheme 5). The structure of ketone **5** is easily established by NMR, just like the structure of the symmetrical alcohols **2** and **8**, and that of the following compounds obtained from it: the ketone **14** and the ethylene ketal compounds **11** and **13**. The X-ray diffraction investigation has confirmed that compound **5** crystalizes in *P*2_1_/n space group of monoclinic system and its molecular crystal consists of isolated neutral molecules, as illustrated in Figure 5. Bond distances and angles are summarized in Table S1.

**Figure 6:**
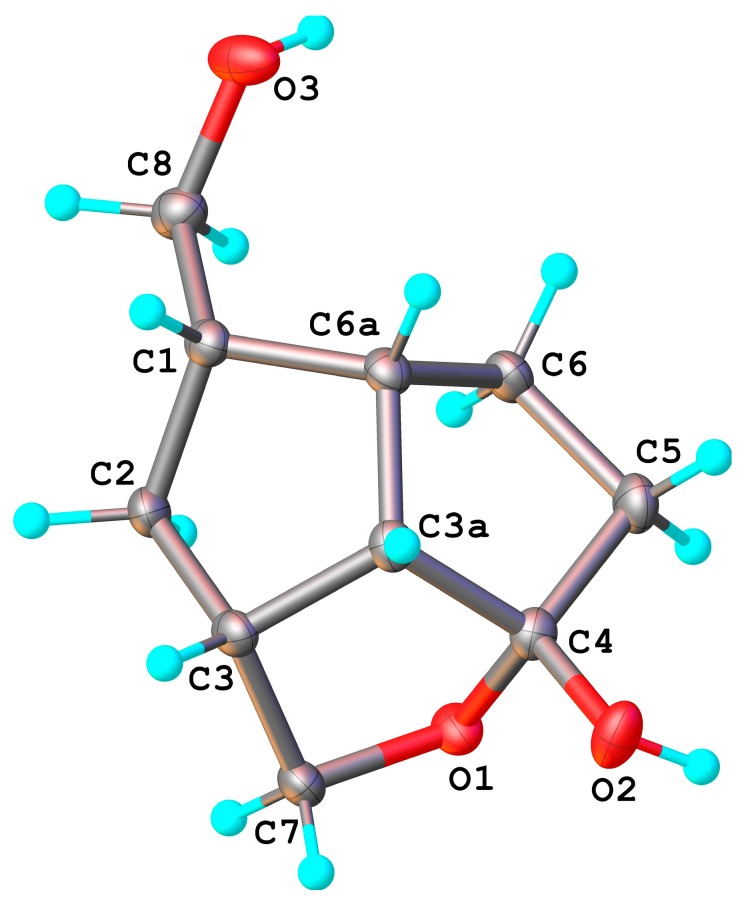
X-ray molecular structure of the un-symmetrical ketone, as internal hemiketal compound **15** (molecule **B**). Thermal ellipsoids are drawn at 50% probability level.

The structure of ketone **6** was also established by NMR spectroscopy. By transesterification (MeONa/MeOH), the benzoate groups were removed and the symmetrical ketone bis-hydroxymethyl compound **14** was obtained from ketone **5**. In the case of the unsymmetrical ketone **6**, during the transesterification of the benzoate groups, the closer hydroxymethyl reacted with the ketone C_4_=O and gave a cyclic hemiketal **15**. Its molecular structure was also confirmed by X-ray crystallography (Figure 6), showing the formation of the tetrahydrofuran ring with *exo*-linked 4-hydroxyl of the hemiketal. According to X-ray crystallography, compound **15** crystallizes in *P*2_1_/n space group of monoclinic system with two crystallographically independent but chemically identical units, denoted as molecules **A** and **B**. In Figure 6 only the structure of molecule **B** is shown.

**Figure 7:**
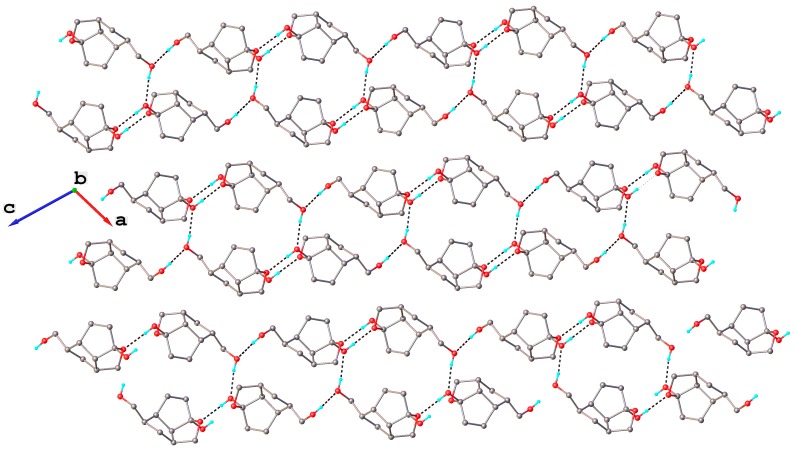
A fragment of the crystal structure of compound **15** viewed along the *b* crystallographic axis.

The crystal packing shows a parallel arrangement of two-dimensional supramolecular layers extended in the 101 plane. A partial view of the crystal structure is shown in Figure 7. Each layer involves both independent molecules **A** and **B** connected by O-H···O hydrogen bonds, the formation of which is completely realized in the crystal. The corresponding hydrogen bond parameters are listed in Table 3.

Both ketone groups of compounds **5** and **6** were transformed into the corresponding ethylene ketals by standard treatment with ethylene glycol (in C_6_H_6_ at reflux, TsOH catalyst). In the first case, we obtained compound **11** which by a similar transesterification reaction, gave compound **13**, with the ketone protected as an ethylene ketal, a group useful for the next reactions for discrimination between the two hydroxymethyl groups. In the case of compound **12**, though the reaction proceeded until all **6** reacted (TLC), during work-up or column chromatography purification, only the starting compound **6** was isolated, indicating that slightly acid conditions favored the deprotection of the ethylene ketal group. The applications of the symmetric-ketone compounds in the synthesis of new carbacyclin analogues are in progress.

## 3. Materials and Methods

### 3.1. General Information

Melting points (uncorrected) were determined in open capillaries on an OptiMelt apparatus (MPA 100, Stanford Research System, Inc., Sunnyvale, CA, USA). The progress of the reactions was monitored by TLC on silica gel 60F_254_ plates (Merck, Darmstadt, Germany) in solvent systems: **I** (benzene–ethyl acetate–hexane, 5:3:2), **II** (cyclohexane–ethyl acetate, 5:1), **III** (ethyl acetate–methanol–acetic acid, 90:13:1), **IV** (acetone–hexanes, 2:1), **V** (hexane–ethyl acetate–acetic acid, 5:3:0.1), **VI** (hexane–ethyl acetate–acetic acid, 5:3:0.1), **VII** (ethyl acetate–hexane–acetic acid, 5:1:0.1). Spots were visualized in UV or with 15% H_2_SO_4_ in MeOH (heating at 110 °C, 10 min) and 2,4-dinitrophenylhydrazine reagent for ketones. The compounds were purified by low pressure chromatography (<2 atm) (LPC), on a glass column, in the solvent systems presented at experimental. IR spectra were recorded on an FT-IR spectrometer 100 (Perkin Elmer, Shelton, CT, USA) and frequencies are expressed in cm^−1^. MS were recorded on a 1200 L/MS/MS triple-quadrupole instrument (Varian, Inc., Walnut Creek, CA, USA) equipped with an ESI interface, fragments obtained by collision with Ar and relative abundances (%) are given in parenthesis. ^1^H-NMR and ^13^C-NMR spectra were recorded on a Varian Gemini 300 BB spectrometer (300 MHz for ^1^H and 75 MHz for ^13^C, Varian, Inc., Palo Alto, CA, USA). Chemical shifts are given in ppm relative to TMS as an internal standard. Complementary spectra: 2D-NMR and decoupling were done for correct assignment of NMR signals. The numbering of the carbon atoms in the compounds is presented in the Schemes.

Crystallographic measurements were carried out with an XCALIBUR E CCD diffractometer (Oxford-Diffraction, Ltd., Abingdon, Oxfordshire, UK) equipped with graphite-monochromated Mo-Kα radiation. Single crystals were positioned at 40 mm from the detector and 481, 258, 251, and 774 frames were measured each for 20, 10, 5, and 20 s over 1° scan width for **8**, **3**, **5** and **15**, respectively. The unit cell determination and data integration were carried out using the CrysAlis package of Oxford Diffraction [38]. The structures were solved by direct methods using the Olex2 [39] software (OlexSys Ltd., Durham University, UK) with the SHELXS structure solution program and refined by full-matrix least-squares method on *F*^2^ with SHELXL-97 [40]. The atomic displacements for the non-hydrogen atoms were refined using an anisotropic model. Hydrogen atoms were placed in fixed, idealized positions and refined as rigidly bonded to the corresponding atoms. Positional parameters of the H attached to the O atoms were obtained from difference Fourier syntheses and verified by the geometric parameters of the corresponding hydrogen bonds. In the absence of significant anomalous scattering, the absolute configuration for **8** could not be reliably determined. Friedel pairs were merged and any references to the Flack parameter were removed. The molecular plots were obtained using the Olex2 program. CCDC: 1566141 (for **8**), 1566142 (for **3**), 1566143 (for **5**) and 1566145 (for **15**). These data can be obtained free of charge via www.ccdc.cam.ac.uk/conts/retrieving.html (or from the Cambridge Crystallographic Data Centre, 12 Union Road, Cambridge CB2 1EZ, UK; fax: (+44) 1223-336-033; or deposit@ccdc.ca.ac.uk).

### 3.2. Hydroboration of 2α,4α-Dimethanol-1β,5β-bicyclo[3.3.0]oct-6-en dibenzoate with Sodium Acetoxy-Borohydride

To a suspension of 98% NaBH_4_ (772 mg, 20 mmol) in anhydrous THF (60 mL), cooled on an ice-water bath, 1.14 mL (1.2 g, 20 mmol) 99.9% acetic acid were added in Ar atmosphere, under stirring for 10 min. and stirring was continued for 20 min. A solution of **1a** (7.53 g, 20 mmol) in THF (15 mL) was added during 20 min, stirring was continued overnight, monitoring the reaction by TLC (I, R_f**1b**_ = 0.91, R_f**3a**_ = 0.47, R_f**2a**_ = 0.35, R_f**4a**_ = 0.05). Water (5 mL) was added, the reaction mixture was cooled on an ice-water bath, then the alkylboranes were oxidized with 30% H_2_O_2_ (6 mL) and 3 M sodium acetate (10 mL) (instead of 3 M NaOH to prevent the hydrolysis of the benzoate protecting groups) both added at the same time during ~15 min, and stirred for 20 min. Ethyl ether (50 mL) was added, phases were separated, aqueous phase was extracted with ether (3 × 50 mL), organic phases were washed with sat. soln. NaHCO_3_ (50 mL), dried (MgSO_4_) and concentrated, obtaining 7.77 of crude product, which was purified by low-pressure chromatography (LPC) (eluent: hexanes, then solvent system: benzene–hexane–ethyl acetate, 5:3:2). The following fractions were eluted:

(a) 1.6 g (21.2%) of unreacted **1a**, (the yields below are based on the reacted **1a**).
(b) 1.54 g (24.8%, based on the reacted alkene) of pure unsymmetrical alcohol (±)-(1α,3α,3aβ,6aβ)-1,2,3,3*a*,4,5,6,6*a*-octahydro-4-hydroxy-1,3-diyl)bis(methylene) dibenzoate (**3a**) as an oil, IR (2% CHCl_3_): 3550–3400, 3350, 3035, 2910–2885, 2850, 1695, 1595, 1580, 1440, 1350, 1270–1200, 1090, 1055, 1040, 945 cm^−1^, ^1^H-NMR (DMSO-*d*_6_, *δ* ppm, *J* Hz): 7.98 (dd, 2H, 8.0, 1.2, H-*o*), 7.95 (dd, 2H, 8.0, 1.2, H-*o*), 7.67–7.61 (m, 2H, 8.0, 1.2, H-*p*), 7.51 (apparent t, 2H, 8.0, H-*m*), 7.50 (t, 2H, 8.0, H-*m*), 4.60 (d, 1H, OH, exchangeable with D_2_O, 4.8), 4.51 (dd,1H, 11.1, 5.0, H-7), 4.18–4.45 (m, 3H, H-7, H-8), 3.82 (m, 1H, H-4), 2.70 (m, 1H, H-3a), 2.20–2.40 (m, 3H, H-6a, H-1, H-3), 1.05–1.90 (m, 6H, H-5, H-6, H-2), ^13^C-NMR (DMSO-*d*_6_, *δ* ppm): 165.75, 165.58 (COO), 133.19, 133.08 (C-*p*), 130.04, 129.81 (C-*q*), 129.12, 129.03 (C-*o*), 128.71, 128.61 (C-*m*); 72.98 (C-4), 65.60 (C-7 or C-8), 65.21 (C-8 or C-7), 51.58 (C-3a), 42.61 (C-1), 40.42 (C-3 or C-6a), 40. 40 (C-6a or C-3), 35.85 (C-5), 31.02 (C-2), 23.01 (C-6), elem. anal. calcd for C_24_H_26_O_5_ (%), C: 73.07, H: 6.64, found: C: 72.80, H: 6.42.
(c) 2.11 g (34%) of pure symmetrical more polar alcohol, (±)-(1α,3α,3aβ,6aβ)-1,2,3,3*a*,4,5,6,6*a*-5-hydroxyoctahydropentalene-1,3-diyl)bis(methylene) dibenzoate (**2a**) which was crystallized (1.69 g) by ethyl acetate-gas extraction, m.p. 115–117 °C. A fraction recrystallized twice had m.p. 119.5–120.5 °C, ^1^H-NMR (CDCl_3_, *δ* ppm, *J* Hz): 8.03 (dd, 4H, 7.1, 1.4, H-*o*), 7.56 (tt, 2H, 7.1, 1.4, H-*p*), 7.44 (t, 4H, 7.1, H-*m*), 4.41 (dd, 2H, 11.0, 7.1, H-7, H-8), 4.31 (dd, 2H, 11.0, 8.2, H-7, H-8), 4.11 (tt, 1H, 5.9, 10.4, H-5), 2.69–2.64 (m, 2H, H-3a, H-6a), 2.48–2.38 (m, 2H, 8.2, 7.1, H-1, H-3), 2.05–1.97 (m, 2H, 10.4, 5.9, H-4, H-6), 1.89 (dt, 1H, 11.1, 5.4, H-2), 1.34 (q, 1H, 11.1, H-2), 1.36–1.26 (m, 2H, 10.4, 5.9, H-4, H-6), ^13^C-NMR (CDCl_3_, *δ* ppm): 166.45 (COO), 132.88 (2C-*p*), 130.24 (2C-*q*), 129.48 (4C-*o*), 128.32 (4C-*m*), 73.33 (C5), 65.58 (C-7, C-8), 41.05 (C-1, C-3 or C-3a, C-6a), 40.85 (C-3a, C-6a or C-1, C-3), 35.98 (C-4, C-6), 30.01 (C-2), elem. anal. Calcd. for C_24_H_26_O_5_, C:d 73.07, H: 6.64, found: C: 72.88, H: 6.52.
(d) 1.72 g (37.6%) pure (±)-(1α,3α,3aβ,6aβ)-1,2,3,3*a*,4,5,6,6*a*-4-hydroxy-3-(hydroxymethyl)-octahydropentalen-1-yl)methyl benzoate (**4a**), which was crystallized by ethyl acetate-gas extraction to give 1.12 g of product, m.p. 74–77 °C, ^1^H-NMR (CDCl_3_, *δ* ppm, *J* Hz): 8.02 (dd, 2H, 1.4, 7.4, H-*o*), 7.53 (t, 1H, 1.4, 7.4, H-*p*), 7.44 (t, 2H, 7.4, 2H-*m*), 4.36 (dd, 1H, 7.1, 11.0, H-8), 4.27 (dd, 1H, 8.0, 11.0, H-8), 4.02 (ddd, 1H, 5.5, 8.2, 10.4, H-4), 3.85 (dd, 1H, 5.8, 11.0, H-7), 3.76 (t, 1H, 11.0, H-7), 3.14–3.00 (br d, 2H, OH), 2.86 (q_v_, 1H, 9.9, H-6a), 2.51 (dt, 1H, 8.2, 9.9, H-3a), 2.44–2.28 (m, 2H, H-1, H-3), 1.98 (dt, 1H, 5.5, 11.0, H-5), 1.75 (m, 1H, H-6), 1.67 (dt, 1H, 5.5, 12.6, H-2), 1.47 (dd, 1H, 5.8, 11.0, H-5), 1.27 (m, 1H, H-6), 0.99 (q, 1H, 12.6, H-2), ^13^C-NMR (CDCl_3_, *δ* ppm): 166.68 (COO), 133.06 (C-*p*), 130.45 (C-*q*), 129.65 (C-*o*), 128.51 (C-*m*), 73.99 (C-4), 65.56 (C-8), 63.50 (C-7), 52.09 (C-3a), 43.68 (C-1), 42.98 (C-6a), 41.11 (C-3), 35.10 (C-5), 30.43 (C-2), 23.76 (C-6).

### 3.3. Hydroboration of 2α,4α-Dimethanol-1β,5β-bicyclo[3.3.0]oct-6-en diacetate with Sodium Acetoxy-Borohydride

Hydroboration of diacetate **1b** (20 mmol) was realized as for **1a**, affording 5.23 g of crude product which was similarly purified. The following fractions were collected: 0.86 g (3.41 mmol) of starting alkene **1b**, (16.9%); 0.13 g slightly impure unsymmetrical alcohol **3b**, as an oil; 1.62 g of a mixture of alcohols **2b** and **3b**, as an oil; 0.26 g of slightly impure symmetric alcohol **2b**, as an oil and 1.74 g (7.6 mmol, 38%) of (±)-(1α,3α,3aβ,6aβ)-1,2,3,3*a*,4,5,6,6*a*-4-hydroxy-3-(hydroxymethyl)octahydro-pentalen-1-yl)methyl acetate (**4b**) which gave 1.32 g (28.8%) as of the title product as needles, m.p. 58–59.5 °C, upon crystallization from ethyl acetate-hexane; ^1^H-NMR (DMSO-*d*_6_, *δ* ppm, *J* Hz): 3.97 (d, 2H, 7.7, H-8), 3.77 (dd + TFA, 1H, 3.3, 6.3, H-4), 3.62 (dd, 1H, 7.6, 10.8, H-7), 3.54–3.40 (br, 2H, OH), 3.45 (dd, 1H, 7.4, 10.8, H-7), 2.57 (dt + TFA, 1H, 6.2, 9.2, H-6a), 2.20 (dt, 1H, 6.3, 8.8, H-3a), 2.08–1.98 (m, 2H, H-1, H-3), 1.98 (s, 3H, CH_3_), 1.68 (m, 1H, H-5), 1.61 (dt, 1H, 6.5, 12.8, H-2), 1.49 (m, 1H, H-6), 1.28 (m, 1H, H-5), 1.13 (m, 1H, H-6), 0.85 (q, 1H, 12.8, H-2), ^13^C-NMR (CDCl_3_, *δ* ppm): 170.40 (COO), 72.72 (C-4), 64.69 (C-8), 61.97 (C-7), 51.83 (C-3a), 43.83 (C-1), 42.53 (C-6a), 40.50 (C-3), 35.40 (C-5), 31.03 (C-2), 23.73 (C-6), 20.73 (CH_3_), elem. anal. calcd. for C_12_H_20_O_4_ (%), C: 63.12, H: 8.83, found: C: 63.20, H: 8.65.

### 3.4. Hydroboration of (±)-(1α,3α,3aβ,6aβ)-1,2,3,3a,4,6a-Hexahydro-1,3-pentalenodimethanol Dibenzoate with Borane (BH_3_·THF)

To a suspension of 98% NaBH_4_ (0.3 g, 7.8 mmol) in anhydrous THF (25 mL), dimethyl sulfate (DMS, 0.76 mL, 1.014 g, 7.8 mmol) was added dropwise under mechanical stirring under an Ar atmosphere. The reaction mixture was stirred for 2 h at room temperature (r.t.) and for 30 min at 50–60 °C, cooled under −10 °C, and a solution of alkene dibenzoate **1a** (7.58 g, 20 mmol; alkene/borane molar ratio of 2.56:1, for forming the trialkylborane) in THF (15 mL) was added dropwise and stirred for 20 h at r.t., following the reaction by TLC. An equal quantity of borane was prepared and added to the reaction mixture at 10–13 °C, then stirred overnight at r.t. Though all the alkene was not consumed, the alkylboranes formed were oxidized, after cooling on an ice-water bath, with 30% H_2_O_2_ (4.4 mL) and 1 N NaOH (8.1 mL), added dropwise at the same time. The stirring was continued then for 20 min at r.t., 40 mL water were added and the mixture was extracted with dichloromethane (3 × 100 mL). Organic phases were washed with brine (2 × 50 mL), dried (MgSO_4_), concentrated and the crude product (7.2 g) was purified as in ex. 3.2, resulting 1.25 g of unreacted alkene (16.6%), 1.90 g (28.8%) **3a**, 2.07 g (31.5%) **2a** and 1.67 g (34.5%) **4a**.

### 3.5. Hydroboration of Alkene-Diol ***7*** with Sodium Acetoxyborohydride

2α,4α-Dimethanol-1β,5β-bicyclo[3.3.0]octene-6 **7** (6.72 g, 40 mmol) dissolved in anhydrous THF (50 mL), was hydroborated with acetoxyborohydride (as described in Section 3.2), obtained from NaBH_4_ (4.63 g, 120 mmol) and 99.9% acetic acid (7.2 mL, 120 mmol) in THF (100 mL) for 3 h at 0–5 °C, and over a weekend at r.t. TLC (III, R_f**7**_ = 0.84, R_f**9**_ = 0.62, R_f**8**_ = 0.41). The alkylborane was oxidized with 30% H_2_O_2_ (33 mL) and 3 N NaOH (60 mL) added dropwise in the same time. The solvent was removed under reduced pressure, the concentrate was co-evaporated with anhydrous ethanol (3 × 50 mL), extracted with ethanol, resulting in an 8.9 g of a mixture of triols **8** and **9**. The pure products were obtained by LPC (silica gel, eluent: acetone–hexanes, 1:2, then 1:1), resulting in 2.57 g (34.5%) of the symmetric alcohol **8** as an oil and 3.58 g (48%) of pure unsymmetric alcohol **9** as a slightly yellow oil (the isolated alcohols were obtained in a 1.0:1.4 ratio of **8/9**).

(a) (±)-(1α,3α,3aβ,6aβ)-1,2,3,3*a*,4,5,6,6*a*-5-Hydroxyoctahydropentalene-1,3-diyl)dimethanol (**8**). The symmetric alcohol was crystallized from acetone-hexanes giving 1.35 g **8** as needle crystals, m.p. 98–99 °C. IR (KBr): 3380–3180, 2945, 2980, 2850, 1480, 1460, 1440, 1350, 1200, 1080, 1020, 960, 870 cm^−1^, ^1^H-NMR (DMSO-*d*_6_, *δ* ppm, *J* Hz): 4.26 (t, 2H, 5.0, HO-C_7,8_, exchangeable with D_2_O), 4.16 (d, 1H, 3.0, HO-C_5_, exchangeable with D_2_O), 4.10 (br s, 1H, H-5), 3.34 (dd, 4H, 7.3, 5.0, H-7, H-8), 2.67–2.60 (m, 2H, H-3a, H-6a), 1.99–1.89 (m, 2H, 7.3, 5.5, H-1, H-3), 1.56 (dt, 1H, 12.1, 5.5, H-2), 1.52–1.44 (m, 2H, H-4, H-6), 1.30–1.20 (m, 2H, H-4, H-6), 0.71 (q, 1H, 12.1, H-2), ^13^C-NMR (DMSO-*d*_6_, *δ* ppm): 72.92 (C-5), 61.97 (C-7, C-8), 44.31 (C-1, C-3 or C-3a, C-6a), 41.98 (C-3a, C-6a or C-1, C-3), 35.30 (C-4, C-6), 30.62 (C-2), MS, *m/z*: 168 (PM), 150, 137 (68%), 109 (73%), 95, 93, 91, 79 (PB).
(b) (±)-(1α,3α,3aβ,6aβ)-1,2,3,3*a*,4,5,6,6*a*-4-Hydroxyoctahydropentalene-1,3-diyl)dimethanol (**9**). IR: practically identical to that of **8**, ^1^H-NMR (DMSO-*d*_6_, *δ* ppm, *J* Hz): 4.65 (dd, 1H, 3.8, 0.5, HO-C_4_, exchangeable with D_2_O), 4.43 (t, 1H, 5.6, HO-C_7_, exchangeable with D_2_O), 4.37 (t, 1H, 4.7, HO-C_8_, exchangeable with D_2_O), 3.76 (dt, 1H, 6.7, 7.3, H-4), 3.62 (dd, 1H, 10.7, 7.7, H-7), 3.46 (dd, 1H, 10.7, 7.5, H-7), 3.37 (d, 2H, 6.0, H-8), 2.56 (d, 1H, 9.0, H-6a), 2.20 (q, 1H, 8.8, H-3a), 2.00 (m, 1H, H-3), 1.90 (m, 1H, H-1), 1.69–1.44 (m, 3H, H-2, H-6, H-5), 1.28 (m, 1H, H-5), 1.17 (m, 1H, H-6), 0.74 (q, 1H, 12.3, H-2), ^13^C-NMR (DMSO-*d*_6_, *δ* ppm): 72.61 (C-4), 61.88 (C-7), 61.37 (C-8), 51.71 (C-3a), 44.30 (C-1), 43.62 (C-3), 42.45 (C-6a), 35.24 (C-5), 31.01 (C-2), 22.83 (C-6), MS, *m/z*: 168 (PM), 150, 138, 119, 106, 93, 91, 83 (PB).

### 3.6. Hydroboration of Alkene-Diol ***7*** with Borane

Alkene **7** (3.26 mmol) was hydroborated with borane in the conditions mentioned in Section 3.4 and using the ratios of borane/**7** mentioned in Table 1. The purification of the crude mixtures was performed as in Section 3.5 and the yields are presented in Table 1.

### 3.7. Hydroboration of Alkene-Dialdehyde ***10*** with Borane (at a 0.359 M Scale)

To a solution of BH_3_·THF, prepared from 98% NaBH_4_ (41 g, 1.056 mol) and 97% dimethyl sulfate (105 mL, 1.056 mol) in THF (500 mL), cooled to <−10 °C, a solution of 2α,4α-diformyl-1β,5β-bicyclo[3.3.0]octene-6 (0.359 mol, previously co-evaporated with benzene) in THF (150 mL) was added dropwise in 90 min, maintaining the temperature under −5 °C. The temperature was left to reach the r.t. and the reaction mixture was further stirred over weekend. TLC (IV, R_f**7**_ = 0.43, R_f**9**_ = 0.23, R_f**8**_ = 0.09). The crude product (75 g) was purified (3×) by LPC (acetone–hexanes, 1:1, then 2:1), resulting 7.55 g (12.5%) alkene **7**, 29.77 g (44.5%) **9** as an oil and 25.1 g (37.5%) **8**, crystallized in mass, m.p. 102–104 °C (twice recrystallized from acetone). The ratio of **9**/**8** was 1.185/1.0.

### 3.8. Benzoylation of Symmetrical Alcohol ***2a***

The dibenzoate alcohol **2a** (160 mg, 0.4 mmol) in pyridine (5 mL) was benzoylated with benzoyl chloride (0.24 mL) in usual conditions. TLC (V, Kieselgel plastifollien, Merck, R_f**2a**_ = 0.57, R_f**2a-triBz**_ = 0.93). The crude product was purified by LPC, resulting 190 mg (95.2%) of symmetrical tribenzoate **2a**, m.p. 85–87 °C (crystallized from hexane–ethyl acetate), ^1^H-NMR (CDCl_3_, *δ* ppm, *J* Hz): 8.05–7.99 (m, 6H, H-*o*), 7.58–7.53 (m, 3H, H-*p*), 7.46–7.40 (m, 6H, H-*m*), 5.57 (t, 1H, 3.3, H-5), 4.43 (dd, 2H, 7.1, 11.0, H-7, H-8), 4.33 (dd, 2H, 8.0, 11.0, H-7, H-8), 3.12–3.03 (m, 2H, H-3a, H-6a), 2.60–2.51 (m, 2H, H-1, H-3), 2.11–2.04 (m, 2H, H-4, H-6), 1.95 (dt, 1H, 5.5, 12.4, H-2), 1.73–1.62 (m, 2H, H-4, H-6), 1.26 (q, 1H, 12.4, H-2), ^13^C-NMR (CDCl_3_, *δ* ppm): 166.63 (COO), 133.09, 132.97 (3C-*p*), 130.24 (3C-*q*), 129.71, 129.67 (6C-*o*), 128.53, 128.46 (6C-*m*), 79.06 (C-5), 65.58 (C-7, C-8), 42.97 (C-1, C-3 or C-3a, C-6a), 40.99 (C-3a, C-6a or C-1, C-3), 33.52 (C-4, C-6), 30.59 (C-2).

### 3.9. Benzoylation of Unsymmetrical Alcohol ***3a***

The dibenzoate alcohol **3a** (160 mg, 0.4 mmol) was benzoylated as above; 150 mg (73%) of un-symmetrical tribenzoate **3a**, m.p. 82–83 °C (hexane-ethyl acetate) were obtained, ^1^H-NMR (CDCl_3_, *δ* ppm, *J* Hz): 8.05 (dd, 2H, 1.4, 7.8, H-*o*), 7.94 (dd, 2H, 1.4, 7.8, H-*o*), 7.87 (dd, 2H, 1.4, 7.4, H-*o*), 7.57 (tt, 1H, 1.4, 7.8, H-*p*-in 4-O-Bz), 7.50–7.23 (m, 8H, 2H-*p*, 6H-*m*), 5.33 (dt, 1H, 5.5, 8.2, H-4), 4.51 (dd, 1H, 6.9, 11.3, H-7 or H-8), 4.51 (dd, 1H, 8.0, 11.3, H-7 or H-8), 4.43 (dd, 1H, 6.9, 11.0, H-8 or H-7), 4.37 (dd, 1H, 8.0, 11.0, H-8 or H-7), 3.00–2.89 (m, 2H, H-3a, H-6a), 2.59–2.42 (m, 2H, H-1, H-3), 2.23 (ddt, 1H, 3.6, 5.8, 12.1, H-5), 1.95 (dt, 1H, 5.5, 12.6, H-2), 1.87 (m, 1H, H-6), 1.68 (m, 1H, H-5), 1.51 (m, 1H, H-6), 1.26 (q, 1H, 12.6, H-2), ^13^C-NMR (CDCl_3_, *δ* ppm): 166.63, 166.45, 166.19 (3COO), 133.11 (C-*p*), 132.85 (2C-*p*), 130.45 (2-C-*q*), 130.24 (C-*q*), 129.65 (6C-*o*), 128.55, 128.31, 128.27 (6C-*m*), 76.58 [C-4, with Cr(acac)3], 65.49 (C-7 or C-8), 64.94 (C-8 or C-7), 49.67 (C-3a), 43.38 (C-1), 41.09, 40.98 (C-3, C-6a), 33.26 (C-5), 31.43 (C-2), 24.23 (C-6).

### 3.10. Benzoylation of Monobenzoate Alcohol ***4a***

The monobenzoate alcohol **4a** (145 mg, 0.5 mmol) was benzoylated as above; 230 mg (73%) of tribenzoate **4a**, m.p. 83–85 °C (hexane–ethyl acetate); ^1^H-NMR (CDCl_3_, *δ* ppm, *J* Hz): 8.05 (dd, 2H, 1.4, 7.4, H-*o*), 7.94 (dd, 2H, 1.4, 7.4, H-*o*), 7.87 (dd, 2H, 1.4, 7.4, H-*o*), 7.57 (tt, 1H, 1.4, 7.8, H-*p*-in 4-*O*-Bz), 7.50–7.42 (m, 2H-*p*), 7.35–7.20 (m, 6H-*m*), 5.33 (dt, 1H, 5.5, 8.2, H-4), 4.51 (dd, 1H, 6.9, 11.3, H-7 or 8), 4.46 (dd, 1H, 8.0, 11.3, H-7 or H-8), 4.43 (dd, 1H, 6.9, 11.0, H-8 or H-7), 4.37 (dd, 1H, 8.0, 11.0, H-8 or H-7), 3.00–2.88 (m, 2H, H-3a, H-6a), 2.60–2.39 (m, 2H, H-1, H-3), 2.23 (ddt, 1H, 3.5, 5.8, 12.1, H-5), 1.94 (dt, 1H, 5.5, 12.6, H-2), 1.87 (m, 1H, H-6), 1.68 (m, 1H, H-5), 1.51 (m, 1H, H-6), 1.26 (q, 1H, 12.6, H-2), ^13^C-NMR (CDCl_3_, *δ* ppm): 166.62, 166.43, 166.18 (3COO), 133.10, 132.85, 132.81 (3C-*p*), 130.445 (2-C-*q*), 130.25 (C-*q*), 129.70, 129.65 (6C-*o*), 128.55, 128.31, 128.27 (6C-*m*), 76.76, 65.49 (C-7 or C-8), 64.94 (C-8 or C-7), 49.70 (C-3a), 43.38 (C-1), 41.09, 40.99 (C-3, C-6a), 33.27 (C-5), 31.43 (C-2), 24.23 (C-6); mixt. m.p. tri-Bz-**2a** + triBz-**3a** = 69–72 °C; mixt. m.p. tri-Bz-**2a** + triBz-**4a** = 72–74 °C; mixt. m.p. tri-Bz-**3a** + triBz-**4a** = 83–85 °C.

### 3.11. Tritylation of Symmetric Triol ***8***

The symmetric triol **7** (3.17 g, 17.02 mmol) was dissolved in pyridine (40 mL) and 97% trityl chloride (10.2 g, 35.6 mmol) was added in portions over 30 min at r.t. under stirring. Stirring was continued over weekend (3 days) and TLC (IV, R_f**7**_ = 0.11, R_f**2c**_ = 0.53, R_f**2c-tris-Tr**_ = 0.80) still showed the presence of a minor quantity of the starting triol. Another 1 g of trityl chloride was added and the solution was stirred for 3 h at 45–50 °C. The cooled reaction mixture was poured over crushed ice, stirred for 1 h, the product was extracted with dichloromethane (3 × 100 mL), the unified organic solutions were washed with sat. soln. NaHCO_3_ (2 × 100 mL), brine (100 mL), dried (MgSO_4_), filtered, concentrated and co-evaporated with toluene. The crude product was crystallized from methanol and recrystallized from methanol-ethyl acetate, resulting 3.84 g of pure compound **2c**, m.p. 98–101 °C [By LPC purification of the concentrated mother liquors (eluent, hexanes–ethyl acetate, 3:1), 6.65 g (total yield: 92%) of pure compound **2c** and 700 mg of almost pure tristrityl derivative (4.5%) were obtained], IR (KBr): 3600–3300, 3040–3020, 2920–2850, 1585, 1480, 1440, 1150, 1055, 740, 695 cm^−1^, ^1^H-NMR (CDCl_3_, *δ* ppm, *J* Hz): 7.43 (dd, 12H, 1.7, 8.5, H-*o*), 7.28 (t, 12H, 8.5, H-*m*), 7.21 (td, 6H, 1.7, 8.5, H-*p*), 4.21 (br s, 1H, H-5), 3.14 (dd, 2H, 6.5, 8.6, H-7, H-8), 2.96–2.85 (m, 2H, H-3a, H-6a), 2.80 (t, 2H, 8.6, H-7, H-8), 2.42–2.34 (m, 2H, 6.5, H-1, H-3), 1.71 (dt, 1H, 12.2, 5.1, H-2), 1.55–1.44 (m, 2H, 6.5, 13.1, H-4, H-6), 1.03–0.95 (m, 2H, H-4, H-6), 0,66 (q, 1H, 12.2, H-2), ^13^C-NMR (CDCl_3_, *δ* ppm): 144.42 (6C-q), 128.70 (12C, C-*o*), 127.65 (12C, C-*m*), 126.79 (6C, C-*p*), 86.22 (2C-q), 75.10 (C-5), 64.26 (2C, C-7, C-8), 42.34 (2C, C-3a, C-6a), 42.14 (2C, C-1, C-3), 35.91 (2C, C-4, C-6), 30.87 (C-2), elem. anal. calcd for C_48_H_46_O_3_: th. C: 85.93, H: 6.91, found: C: 85.10 (−0.83, due to traces of solvent, not determined), H: 6.79.

### 3.12. PDC Oxidation of Alcoholdibenzoate ***2a***

To alcohol **2a** (1.2 g, 2.84 mmol) dissolved in dichloromethane (60 mL), PDC (1.65 g, 4.26 mM) and molecular sieve (2 g) were added and stirred overnight, monitoring the reaction by TLC (I, R_f**2a**_ = 0.40, R_f**5a**_ = 0.66). The reaction mixture was diluted with ethyl ether, filtered of a anh. MgSO_4_ bed, the bed was washed with ether, the filtrate was concentrated and the crude product was purified by LPC (hexanes–ethyl acetate, 3:1 or benzene–ethyl acetate–hexane 5:3:2) and the pure ketone, (±)-(1α,3α,3aβ,6aβ)-1,2,3,3*a*,4,6*a*-5-oxooctahydropentalene-1,3-diyl)bis(methylene) dibenzoate (**5**) was obtained in more than 90% yield as an oil, which crystallized in time, m.p. 79.5–81.5 °C (ethyl ether–hexane), IR (1% in CHCl_3_): 3030, 2910–1880, 1700–1690, 1595, 1580, 1450, 1250–1200, 1100, 950 cm^−1^, ^1^H-NMR (CDCl_3_, *δ* ppm, *J* Hz): 8.00 (d, 4H, 7.1, H-*o*), 7.57 (t, 2H, 7.1, H-*p*), 7.44 (d, 4H, 7.1, H-*m*), 4.39 (dd, 2H, 11.4, 6.3, H-7, H-8), 4.30 (dd, 2H, 11.4, 7.6, H-7, H-8), 3.10–3.03 (m, 2H, H-1, H-3), 2.38 (dd, 2H, 18.6, 6.2, H-4, H-6), 2.30 (dd, 2H, 18.6, 6.5, H-4, H-6), 2. 08 (dt, 1H, 12.3, 6.5, H-2), 1.46 (q, 1H, 12.3, H-2), ^13^C-NMR (CDCl_3_, *δ* ppm): 218.50 (C-5), 166.26 (COO), 133.01 (C-*p*), 129.87 (C-q), 129.42 (C-*o*), 128.36 (C-*m*), 65.25 (2C, C-7, C-8), 41.09 (2C, C-3a, C-6a), 40.29 (2C, C-1, C-3), 38.71 (2C, C-4, C-6), 30.47 (C-2), elem. anal. calcd. for C_24_H_24_O_5_, C: 73.45, H: 6.16, found: C: 73.49, H: 6.22.

### 3.13. PDC Oxidation of Alcoholdibenzoate ***3a***

Dibenzoate alcohol **3a** (0.81 g, 2.84 mmol) in dichloromethane (50 mL), was oxidized as before with PDC (1.20 g, 3.19 mmol) and molecular sieve (1.35 g), overnight. TLC (I, R_f**3a**_ = 0.50, R_f**6a**_ = 0.68) showed still the presence of **3a** and more PDC (0.35 g) were added and stirring was continued for 3 days. After work-up and purification as above, 0.67 g (83.3%) of pure ketone, (±)-(1α,3α,3aβ,6aβ)-1,2,3,3*a*,4,6*a*-4-oxooctahydropentalene-1,3-diyl)bis(methylene) dibenzoate (**6**) were obtained as an oil, which crystallized overnight in the refrigerator, m.p. 61–63 °C, IR (1% CHCl_3_), practically the same as those of ketone **5**: 3030, 2910–2880, 1700–1690, 1595, 1580, 1450, 1250–1200, 1100, 1060, 950 cm^−1^, ^1^H-NMR (CDCl_3_, *δ* ppm, *J* Hz): 8.05 (dd, 2H, 8.3, 1.4, H-*o*), 7.98 (dd, 2H, 8.3, 1.4, H-*o*), 7.58 (tt, 1H, 8.3, 1.4, H-*p*), 7.55 (tt, 1H, 8.3, 1.4, H-*p*), 7.45 (t, 2H, 8.3, H-*m*), 7.43 (t, 2H, 8.3, H-*m*), 4.55 (dd, 1H, 11.4, 3.6, H-7 or H-8), 4.48 (dd, 1H, 6.9, 11.1, H-8 or H-7), 4.44 (dd, 1H, 11.1, 6.9, H-7 or H-8), 4.43 (dd, 1H, 11.4, 7.9, H-8 or H-7), 3.01 (m, 1H, H-1), 2.83 (t, 1H, 10.8, H-3a), 2.78 (m, 1H, H-3), 2.65 (m, 1H, H-6a), 2.20–2.28 (m, 2H, H-5), 2.00–2.15 (m, 2H, H-2, H-6), 1.80 (dq, 1H, 13.0, 10.2, H-6), 1.62 (q, 1H, 12.5, H-2), ^13^C-NMR (CDCl_3_, *δ* ppm): 219.83 (C-6), 166.30, 166.05 (COO), 132.95, 132.84 (C-*p*), 129.98, 129.93 (C-q), 129.40, 129.22 (C-*o*), 128.31, 128.28 (C-*m)*, 64.81 (C-8 or C-7), 63.73 (C-7 or C-8), 51.90 (C-3a), 43.83 (C-1), 41.29, 41.20 (2C, C-3, C-6a), 39.66 (C-5), 30.76 (C-2), 22.16 (C-6), elem. anal. calcd for C_24_H_24_O_5_, C: 73.45, H: 6.16, found: C: 73.90, H: 6.24.

### 3.14. PDC Oxidation of Alcohol ***2c***

The compound **2c** (3.42 g, 5.1 mmol) was oxidized (as in 3.12) [CH_2_Cl_2_ (100 mL), PDC (3 g), molecular sieves (3.8 g), r.t., 2 days]; TLC (II, R_f**2c**_ = 0.32, R_f**5c**_ = 0.57); 3.31 g (97%) of pure **5c** were obtained. By recrystallization from ethyl ether, 2.35 g of crystallized (±)-(1α,3α,3aβ,6aβ)-1,2,3,3*a*,4,6*a*-1,3-bis((trityloxy)methyl)hexahydropentalen-5-one (**5**) were obtained, m.p. 189–191 °C, IR (KBr): 3020, 2910, 2860, 1730, 1590, 1480, 1445, 1150, 1060, 750, 700 cm^−1^, ^1^H-NMR (CDCl_3_, *δ* ppm, *J* Hz): 7.40 (dd, 12H, 7.9, 1.2, H-*o*), 7.28 (t, 12H, 7.9, H-*m*), 7.23 (tt, 6H, 7.9, 1.2, H-*p*), 3.13 (dd, 2H, 8.8, 5.8, H-7, H-8), 3.04–2.95 (m, 2H, H-3a, H-6a), 2.80 (t, 2H, 8.8, H-7, H-8), 2.57–2.45 (m, 2H, H-1, H-3), 2.06 (dd, 2H, 19.1, 9.5, H-4, H-6), 1.87 (dd, 2H, 19.1, 5.9, H-4, H-6), 1.82 (dt, 1H, 12.4, 6.2, H-2), 1.00 (q, 1H, 12.4, H-2), ^13^C-NMR (CDCl_3_, *δ* ppm): 220.42 (C-5), 144.05 (6 Cq, Tr), 128.53 (12 C-*m*), 127.72 (12 C-*o*), 126.93 (6 C-*p*), 86.40 (2Cq, Tr), 64.30 (C-7, C-8), 41.75 (2C, C-3a, C-6a or C-1, C-3), 40.88 (2C, C-1, C-3 or C-3a, C-6a), 38.76 (2C, C-4, C-6), 30.89 (C-2).

### 3.15. Synthesis of (±)-(1α,3α,3aβ,6aβ)-1,2,3,3a,4,6,6a-Hexahydro-1H-spiro[pentalene-4,4′-[1,3]dioxolane]-1,3-diyl)bis(methylene)dibenzoate *(**11**)* from Ketone ***5a***

The symmetric ketone **5a** (0.93g, 2.37 mmol) was dissolved in benzene (25 mL), ethylene glycol (0.4 mL) and TsOH (10 mg) were added, and refluxed for 5 h (Dean-Stark trap for removing the water) monitoring the reaction by TLC (VI, R_f**5a**_ = 0.69, R_f**11**_ = 0.77). The reaction mixture was cooled, washed with sat. sol. NaHCO_3_ (2 × 20 mL), brine (20 mL), concentrated, and ketone **11** was obtained in quantitative yield by crystallization from benzene-hexane; the product has m.p. 99–100 °C, IR (KBr): 3045, 2945, 2870, 1690, 1590, 1580, 1445, 1260, 1160, 1090, 1060, 1015, 960, 940, 870, 695 cm^−1^, ^1^H-NMR (CDCl_3_, *δ* ppm, *J* Hz): 8.04 (dd, 4H, 8.3, 1.2, H-*o*), 7.75 (tt, 2H, 7.2, 1.2, H-*p*), 7.44 (td, 4H, 8.3, 7.2, H-*m*), 4.40 (dd, 2H, 11.1, 6.8, H-7, H-8), 4.27 (dd, 2H, 11.1, 8.4, H-7, H-8), 3.92 (apparent t, 4H, O-CH_2_CH_2_-O), 2.90–2.83 (m, 2H, H-3a, H-6a), 2.55–2.46 (m, 2H, 6.8, 8.4, H-1, H-3), 1.91 (dt, 1H, 12.5, 5.3, H-2), 1.82 (dd, 2H, 12.7, 8.6, H4, H-6), 1.68 (dd, 2H, 12.7, 9.6, H-4, H-6), 1.32 (q, 1H, 12.5, H-2), ^13^C-NMR (CDCl_3_, *δ* ppm): 166.46 (COO), 132.93 (C-*p*), 130.54 (C-q), 129.60 (C-*o*), 128.40 (C*-m*), 117.86 (C-5), 65.54 (C-7, C-8), 64.91, 64.26 (OCH_2_CH_2_O), 41.14 (C-3a, C-6a or C-1, C-3), 41.03 (C-1, C-3 or C-3a, C-6a), 35.46 (C-4, C-6), 30.29 (C-2).

### 3.16. Synthesis of the Ethylene Ketal of the Unsymmetric Ketone ***6a***

The same reaction conditions: **6a** (0.93 g, 2.37 mmol); TLC (VI, R_f**5a**_ = 0.69, R_f**11**_ = 0.83). The crude product (0.73 g) was purified similarly, resulting 605 mg (81.2%) of pure starting ketone **6a** (IR and NMR as for compound **6a**).

### 3.17. Synthesis of Symmetrical Ketone ***14*** by Deprotection of the Benzoate Groups of ***5a***

To a soln. of bisbenzoate ketone **5a** (0.4 g, 1.02 mmol) in methanol (30 mL), a solution of MeONa (0.065 g/mL) (1 mL) was added and stirred at r.t. for 3 h, monitoring the reaction by TLC (VII, R_f**14**_ = 0.18). The base was neutralized with methanol-washed Dowex 50 × 4, the filtrate was concentrated and the crude product was purified by LPC (eluent, ethyl acetate–hexanes, 5:1), resulting a pure fraction of 165 mg (86.8%) of (±)-(1α,3α,3aβ,6aβ)-1,2,3,3*a*,4,6,6*a*-1,3-bis(hydroxymethyl)-hexahydropentalen-5-one (**14**) as an oil, ^1^H-NMR (DMSO-*d*_6_, *δ* ppm, *J* Hz): 3.55 (dd, 2H, 10.7, 5.7, H-7, H-8), 3.27 (dd, 2H, 10.7, 7.4, H-7, H-8), 2.81–2.70 (m, 2H, H-3a, H-6a), 2.22–2.12 (m, 2H, H-1, H-3), 2.15 (dd, 2H, 18.5, 5.9, H-4, H-6), 2.07 (dd, 2H, 18.5, 9.5, H-4, H-6), 1.66 (dt, 1H, 12.5, 5.0, H-2), 1.11 (q, 1H, 12.5, H-2), ^13^C-NMR (DMSO-*d*_6_, *δ* ppm): 220.02 (C=O), 61.90 (2C, C-7, C-8), 42.92 (2C, C-1, C-3), 41.02 (2C, C-3a, C-6a), 38.36 (2C, C-4, C-6), 30.07 (C-2).

### 3.18. Synthesis of (±)-(1α,3α,3aβ,6aβ)-1,2,3,3a,4,6,6a-Hexahydro-1H-spiro[pentalene-4,4'-[1,3]dioxolane]-1,3-diyl)dimethanol *(**13**)*

Ethylene ketal **11** (0.49 g, 1.12 mmol) was hydrolyzed as described above for compound **5a**: TLC (VII, R_f**11**_ = 0.81, R_f**13**_ = 0.20); LPC (ethyl acetate-hexane, 5:1); 235 mg (91.2%) of pure compound **13** were obtained as an oil, ^1^-H-NMR (CDCl_3_, *δ* ppm, *J* Hz): 3.91 (m, 4H, OCH_2_CH_2_O), 3.59 (d, 4H, 7.4, H-7, H-8), 3.17 (br s, 2H, exchangeable with D_2_O, OH), 2.75–2.68 (m, 2H, H-3a, H-6a), 2.20–2.10 (m, 2H, H-1, H-3), 1.79 (dt, 1H, 12.4, 5.4, H-2), 1.72 (dd, 2H, 14.6, 8.5, H-4, H-6), 1.55 (dd, 2H, 14.6, 7.7, H-4, H-6), 1.05 (q, 1H, 12.4, H-2), ^13^C-NMR (CDCl_3_, *δ* ppm): 118.02 (C-5), 64.63, 63.95 (OCH_2_CH_2_O), 63.30 (2C, C-7, C-8), 44.32 (C-1, C-3), 40.73 (C-3a, 6a), 34.84 (2C, C-4, C-6), 30.02 (C-2).

### 3.19. Deprotection of Benzoate Groups of Compound ***6a*** by Transesterification

The benzoate groups of the compound **6a** (0.41 g, 1.04 mmol) were hydrolyzed by transesterification, as described above (TLC, VII, R_f**12**_ = 0.24, less active to dinitrophenylhydrazine); to give 185 mg (quantitative yield) of (±)-(2*a*α,2*a*1α,4α,4*a*α,6*a*α)-4-(hydroxymethyl)octahydro-6*aH*-pentaleno[1,6-*bc*]furan-6*a*-ol (**15**) as a cyclic hemiketal. The compound was crystallized from ethyl acetate, m.p. 106–108 °C, IR: 3442, 3353, 3250, 2952, 2913, 2878, 2858, 1462, 1428, 1315, 1078, 1056, 1024, 981, 898, 790 cm^−1^, ^1^H-NMR (DMSO-*d*_6_, *δ* ppm, *J* Hz): 5.75 (s, 1H, OH-4, exchangeable with D_2_O), 4.29 (t, 1H, 4.7, OH-8, exchangeable with D_2_O,), 3.82 (dd, 1H, 8.5, 4.8, H-7), 3.53 (d, 1H, 8.5, H-7), 3.28 (d, 2H, 7.3, H-8), 2.61 (t, 1H, 8.6, H-3a), 2.45–2.60 (m, 2H, H-6a. H-3), 1.96 (m, 1H, 6.6, H-1), 1.86 (dd, 1H, 12.4, 6.5, H-5), 1.80 (m, 1H, 12.6, H-2), 1.54 (dt, 1H, 13.3, 6.5, H-6), 1.43 (dt, 1H, 13.3, 6.9, H-5), 1.25 (m, 1H, 12.4, 10.2, 6.0, H-6), 1.01 (td, 1H, 12.6, 8.9, H-2), ^13^C-NMR (DMSO-*d*_6_, *δ* ppm): 115.00 (C-4), 71.74 (C-7 ), 61.38 (C-8), 58.90 (C-3a), 47.16 (C-1), 44.14 (C-6a or C-3), 43.72 (C-3 or C-6a), 38.44 (C-5), 33.37 (C-2), 24.02 (C-6). ^1^H-NMR (CDCl_3_, *δ* ppm, *J* ppm): 4.05 (dd, 1H, 8.8, 5.1, H-7), 3.75 (d, 1H, 8.8, H-7), 3.58 (d, 2H, 7.3, H-8), 2.86 (t, 1H, 9.0, H-3a), 2.77–2.66 (m, 2H, H-3, H-6a), 2.15 (m, 1H, H-1), 2.05 (dd, 1H, 6.3, 12.4, H-6), 1.93 (ddd, 1H, 12.6, 8.4, 6.1, H-2), 1.78 (td, 1H, 12.5, 6.8, H-5), 1.59 (dt, 1H, 12.5, 7.4, H-5), 1.43 (ddt, 1H, 11.7, 12.4, H-6), 1.20 (dt, 1H, 12.6, 9.6, H-2), ^13^C-NMR (CDCl_3_, *δ* ppm): 115.00 (C-5), 71.44 (C-7), 61.38 (C-8), 58.90 (C-3a), 47.16 (C-1), 44.14 (C-6a or C-3), 43.72 (C-3 or C-6a), 38.44 (C-5), 33.72 (C-2), 24.02 (C-6). The molecular structure was confirmed by X-ray crystallography (Figure 7).

## 4. Conclusions

Hydroboration-oxidation of 2α,4α-dimethanol-1β,5β-bicyclo[3.3.0]oct-6-en dibenzoate (**1**) gave the alcohols **2** (symmetric) and **3** (unsymmetric) in ~60% yield, together with the monobenzoate diol **4a** (37%), resulting from by the reduction of the closer benzoate by the intermediate alkylborane. Alkene **7** and dialdehyde **10** gave only the triols **8** and **9** in ~1:1 ratio, in >73% yield. By increasing the reaction time and the reaction temperature, the isomerization of alkylboranes favors the un-symmetrical triol **9**. The mechanism of reduction of the closer ester to 4-alkylborane and the isomerization of alkylboranes toward the unsymmetrical regioisomer were also presented. The oxidation of the secondary alcohols cleanly gave the corresponding ketones **5** and **6** and the deprotection of the benzoate groups gave the symmetrical ketone **14**, and the cyclic hemiketal **15**, all in high yields. The ethylene ketals of the symmetrical ketones **11** and **13** were also obtained. In conclusion, different symmetric and un-symmetric ketones and protected ketone compounds were synthesized. They are potentially useful key intermediate for the synthesis of new carbacyclin and/or isocarbacyclin analogues with different structural radicals. The structure of the compounds was established by NMR spectroscopy and confirmed by X-ray crystallography.

## Figures and Tables

**Table 1:** Hydroboration of the alkene-diol **7** with borane.

Molar Ratio 7/BH_3_·THF	Reaction Time (h)	Molar Ratio 8/9	Yield (%)		
			8	9	8 + 9
1:1.2	7	1.0:1.2	13.3	16.00	29.3
1:2.0	7	1.0:1.6	15.8	25.0	40.8
1:3.0	6	1.0:1.05	31.4	33.1	64.5
1:4.0	6	1.0:1.07	36.7	38.6	73.3

**Table 2:** H-bond parameters for compound **8**.

D-H···A	Distance, Å			Angle D-H···A, deg	Symmetry Code
	D-H	H···A	D···A		
O1–H···O3	0.82	1.90	2.701(2)	163.9	−0.5 − *x*, *y,* 0.5 + *z*
O2–H···O1	0.82	1.93	2.729(2)	170.9	1 − *x*, −1 − *y*, −0.5 + *z*
O3–H···O2	0.82	1.90	2.674(2)	156.8	0.5 + *x*, −1.5 − *y*, *z*

**Table 3:** H-bond parameters for compound **15**.

D–H···A	Distance, Å			Angle D-H···A, deg	Symmetry Code
	D-H	H···A	D···A		
O2A–H···O1B	0.82	1.94	2.754(2)	171.8	1 − *x*, 1 − *z*
O3A–H···O3B	0.82	2.01	2.794(2)	160.8	0.5 − *x*, 0.5 + *y*, 1.5 − *z*
O2B–H···O2A	0.82	1.96	2.781(2)	173.8	−1 − *x*, 1 − *y*, 1 − *z*
O2B–H···O3A	0.82	1.94	2.757(2)	171.4	−1 + *x*, *y*, *z*

## Supplementary Materials

The Supplementary Material associated with this article (^1^H-NMR and ^13^C-NMR spectra for compounds, crystallography data in Table S1 for compounds **8**, **3**, **5** and **15**) are available online. Also CIF files and cifreport for compounds: **8** (shI_3623-CoTa.cif), **3** (shI_3624-CoTa.cif), **5** (shI_3625-CoTa.cif) and **15** (shI_1265-CoTa.cif) are available as Supplementary material.
