# Supplementary material for: Hydroboration-Oxidation of (±)-(1α,3α,3aβ,6aβ)-1,2,3,3a,4,6a-Hexahydro-1,3-pentalenedimethanol and Its O-Protected Derivatives: Synthesis of New Compounds Useful for Obtaining (iso)Carbacyclin Analogues and X-ray Analysis of the Products
Source: Molecules. 2017 Nov 24;22(12):2032. doi: 10.3390/molecules22122032 (PMC6149920; doi:10.3390/molecules22122032)
Supplement: Supplementary file 1 [file molecules-22-02032-s001.pdf]

Supplementary Materials

Hydroboration-oxidation of (±)-(1α,3α,3aβ,6aβ)-1,2,3,3a,4,6a-Hexahydro-1,3-pentalenedimethanol and its O-Protected Derivatives: Synthesis of New Useful Compounds for Obtaining (Iso)carbacyclin Analogues and X-ray Analysis of the Products.

Contents

1. NMR Spectra of the compounds.

2. X-ray crystallography of compounds **3, 5, 8** and **15** (Tables S1 and S2).

1. NMR Spectra of the compounds.

1.1. ^{1}H, ^{1}H + TFA and ^{13}C spectra of compound **2a**

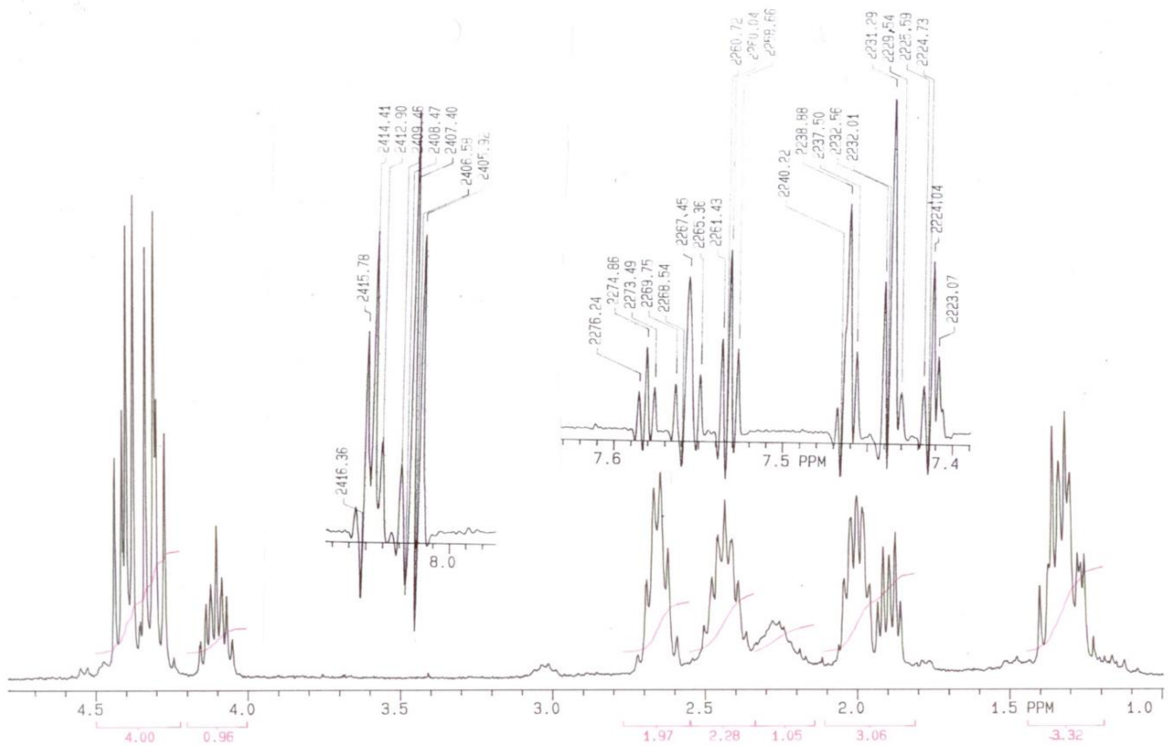
2416.36
2415.78
2414.41
2412.90
2409.46
2408.47
2407.40
8.0
2276.24
2274.86
2273.49
2269.75
2268.54
2267.45
2265.36
2261.43
2260.72
2240.22
2238.88
2237.50
2232.56
2232.01
2231.29
2229.54
2224.73
2224.04
2223.07
7.6
7.5 PPM
7.4
4.5
4.0
3.5
3.0
2.5
2.0
1.5 PPM
1.0
4.00
0.96
1.97
2.28
1.05
3.06
3.32

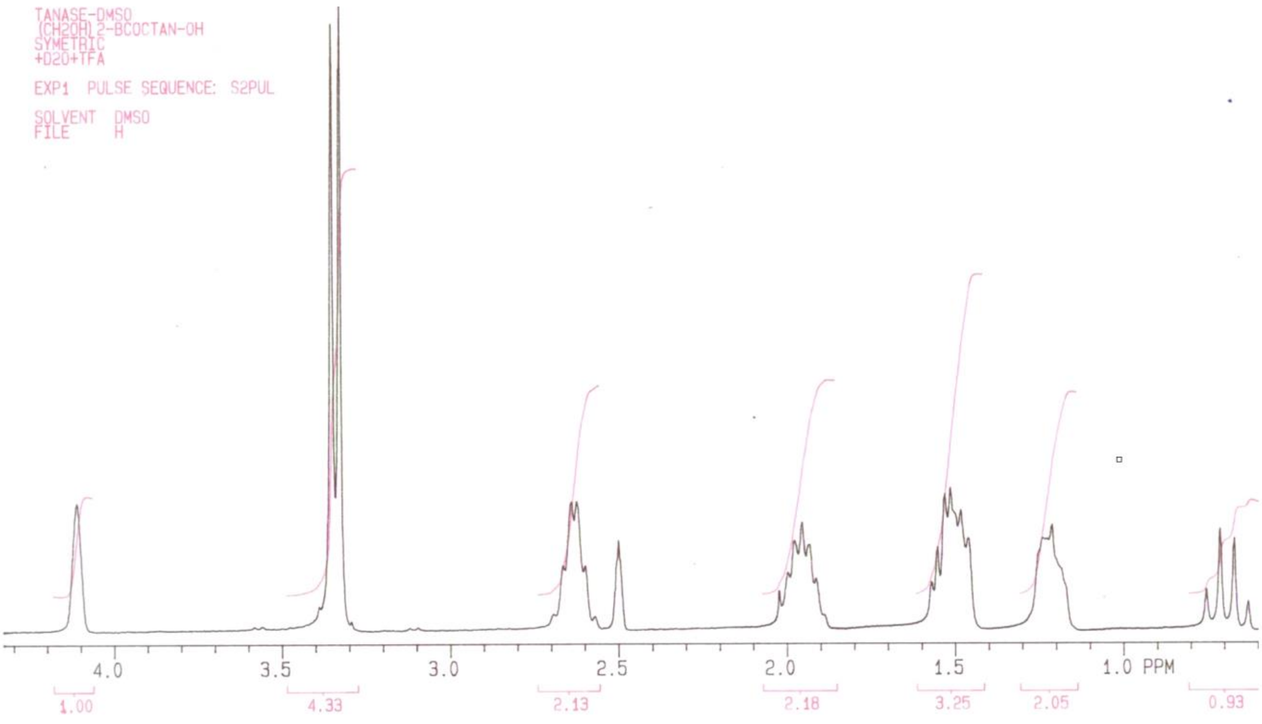
TANASE-DMSO
(CH2OH)2-BCOCTAN-OH
SYMETRIC
+D2O+TFA
EXP1 PULSE SEQUENCE: S2PUL
SOLVENT DMSO
FILE H
4.0
3.5
3.0
2.5
2.0
1.5
1.0 PPM
1.00
4.33
2.13
2.18
3.25
2.05
0.93

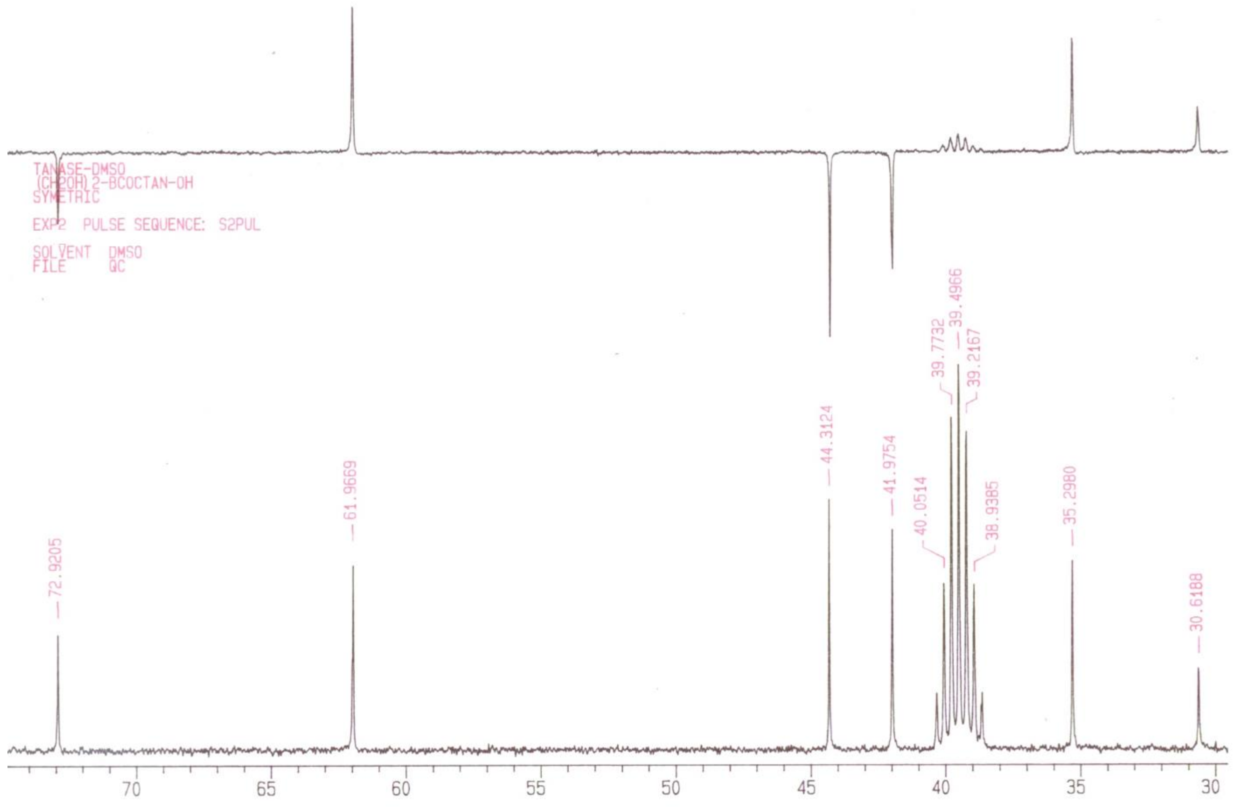

1.2. ^{1}H and ^{13}C spectra of compound **3a**

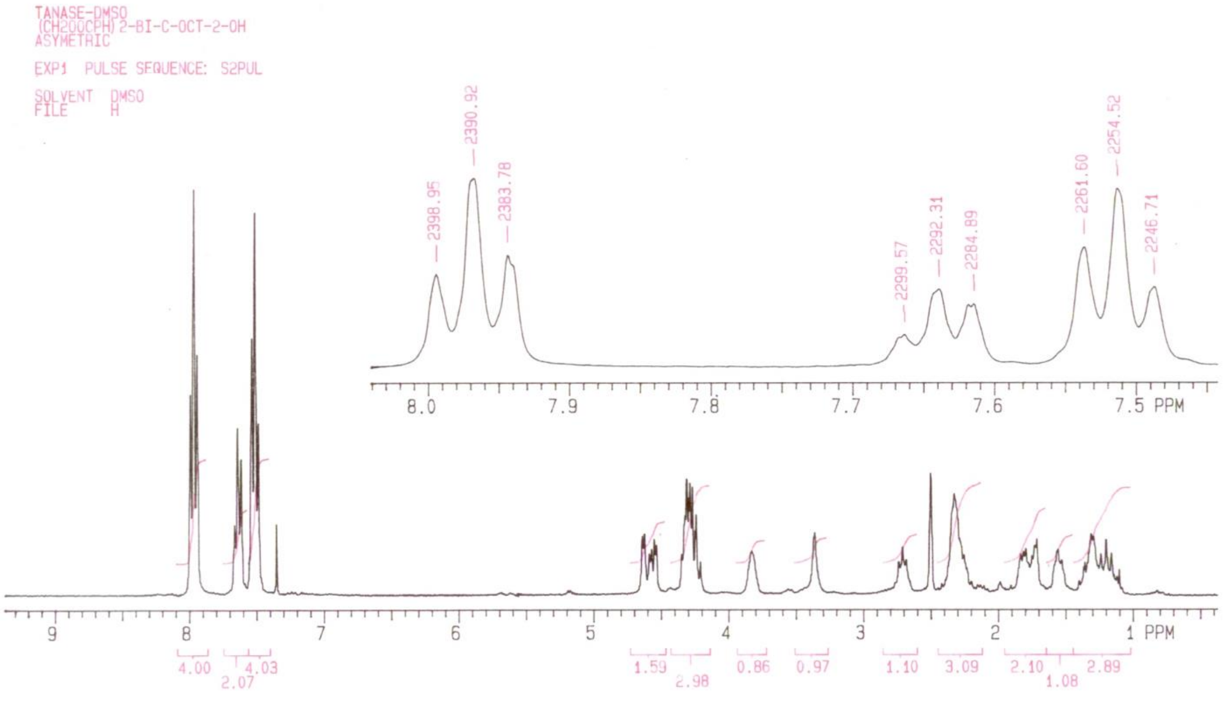

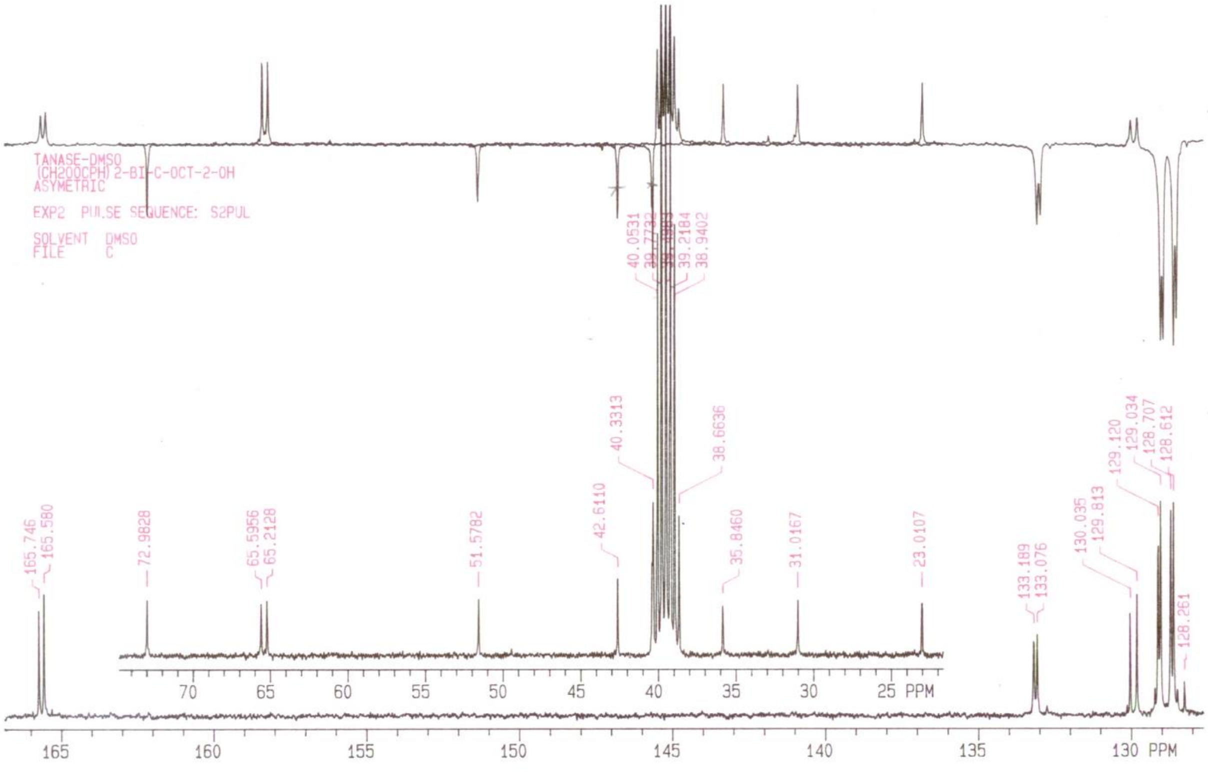
TANASE-DMSO
(CH2OOCPH)2-BI-C-OCT-2-OH
ASYMETRIC
EXP2 PULSE SEQUENCE: S2PUL
SOLVENT DMSO
FILE C
40.0531
39.2184
38.9402
40.3313
38.6636
165.746
165.580
72.9828
65.5956
65.2128
51.5782
42.6110
35.8460
31.0167
23.0107
133.189
133.076
130.035
129.813
129.120
129.034
128.707
128.612
128.261
70
65
60
55
50
45
40
35
30
25 PPM
165
160
155
150
145
140
135
130 PPM

1.3. ^{1}H, ^{13}C, COSY and HETCOR spectra of compound **4a**

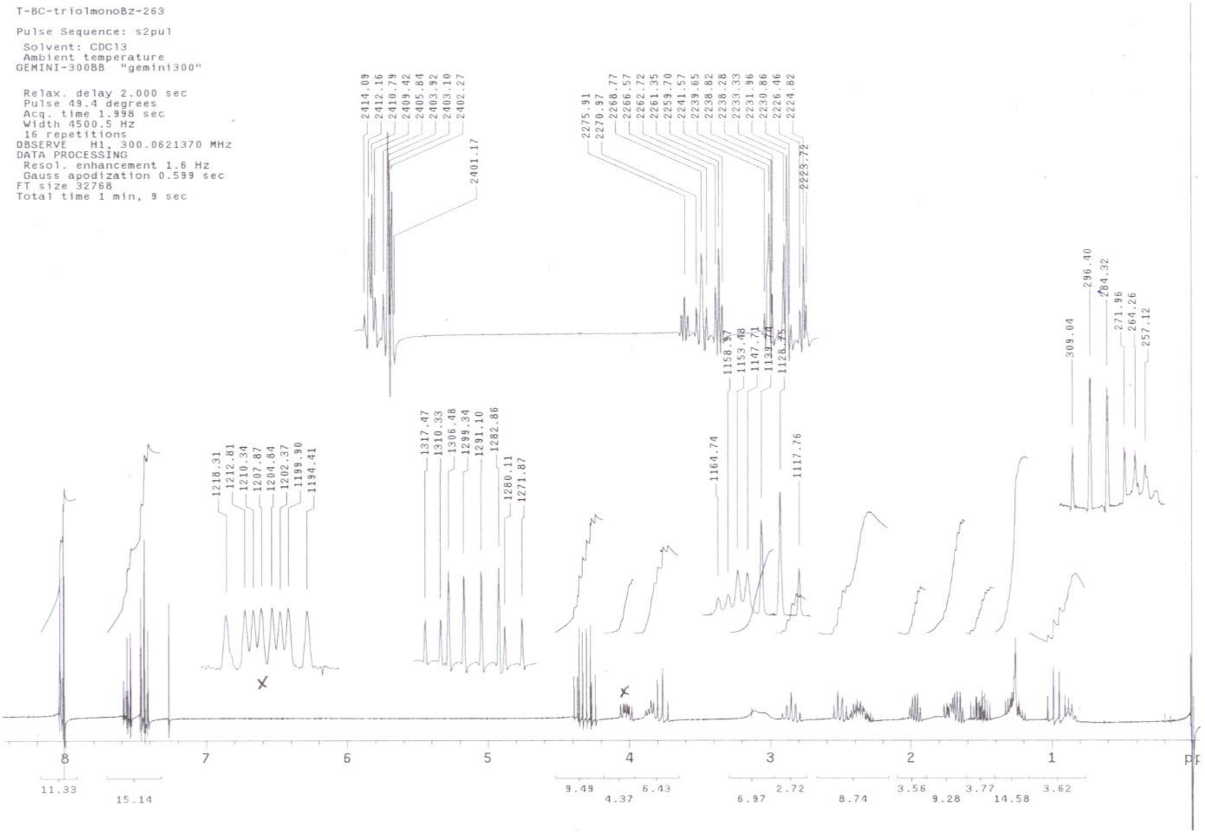

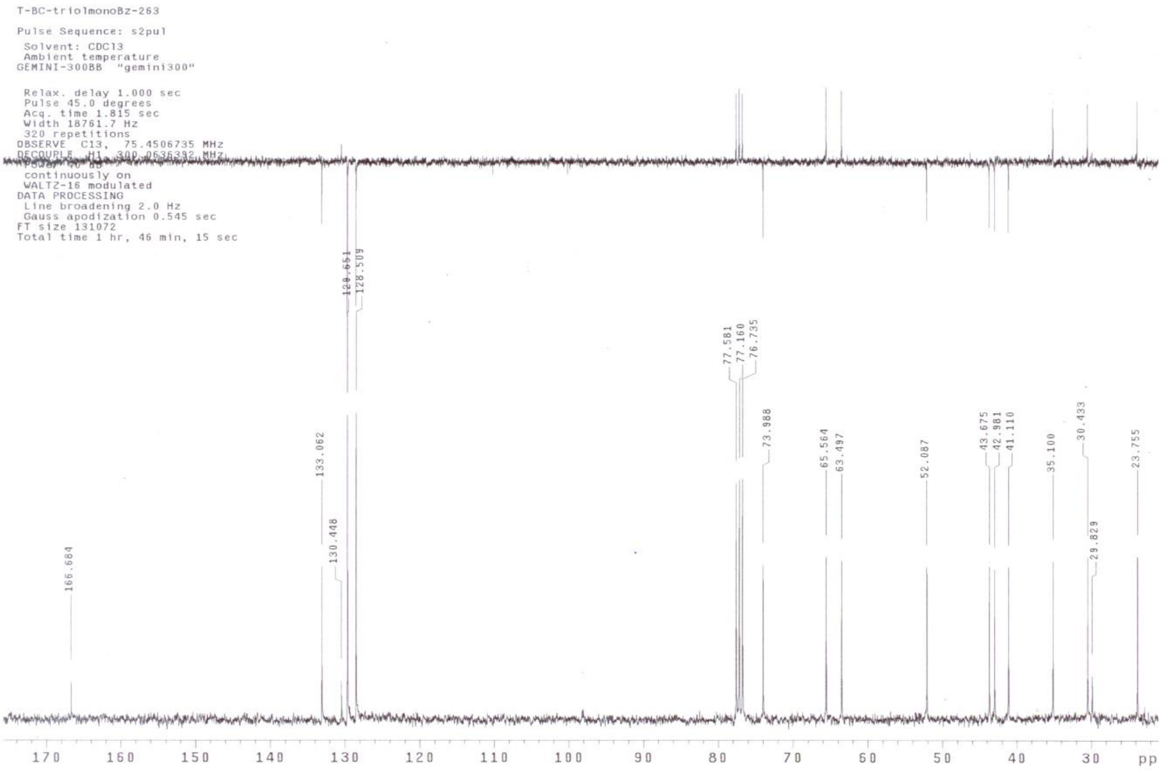

T-BC-triolmonoBz-263

Pulse Sequence: relayh

Solvent: CDCl3
Ambient temperature
GEMINI-300BB "gemini300"

Relax. delay 1.000 sec
COSY 90-90
Acq. time 0.224 sec
Width 1145.0 Hz
2D Width 1145.0 Hz
4 repetitions
256 increments
OBSERVE H1, 300.0621370 MHz
DATA PROCESSING
Sine bell 0.112 sec
F1 DATA PROCESSING
Sine bell 0.056 sec
FT size 512 x 512
Total time 24 min, 34 sec

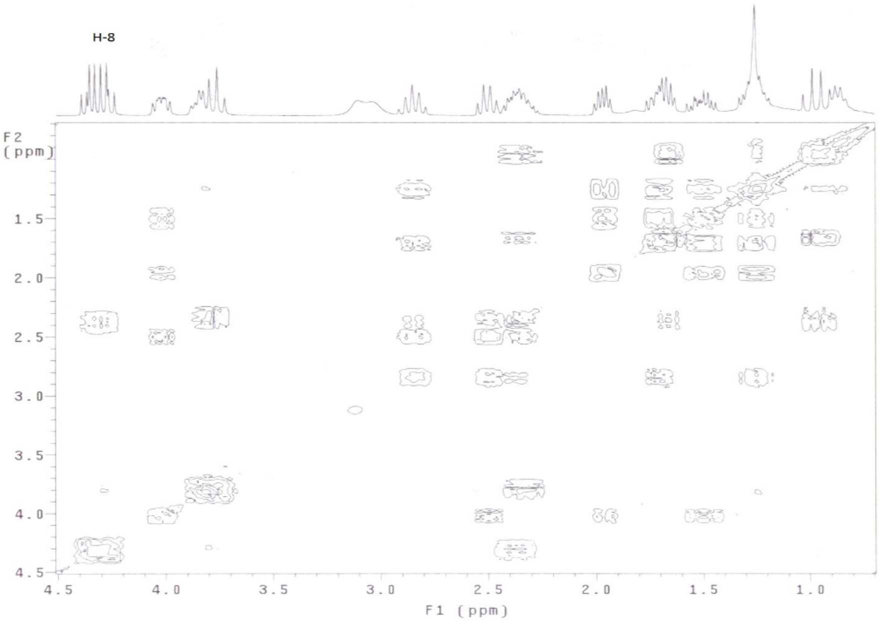

T-BC-triolmonoBz-263

Pulse Sequence: hetcor

Solvent: CDCl3
Ambient temperature
GEMINI-300BB "gemini300"

Relax. delay 1.000 sec
Acq. time 0.063 sec
Width 4040.8 Hz
2D Width 1179.4 Hz
128 repetitions
64 increments
OBSERVE C13, 75.4506735 MHz
DECOUPLE H1, 300.0629045 MHz
Power 40 dB
on during acquisition
off during delay
WALTZ-16 modulated
DATA PROCESSING
Line broadening 2.0 Hz
Gauss apodization 0.545 sec
F1 DATA PROCESSING
Line broadening 0.3 Hz
FT size 512 x 256
Total time 2 hr, 37 min, 52 sec

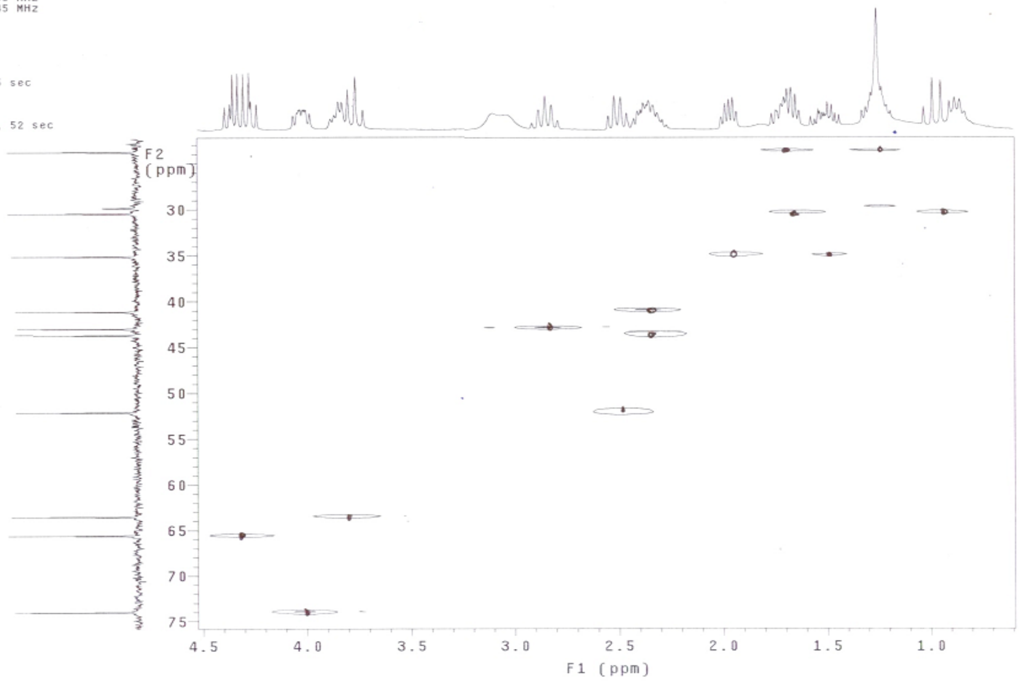

1.4. ^{1}H, ^{13}C, COSY and HETCOR spectra of compound **4b**

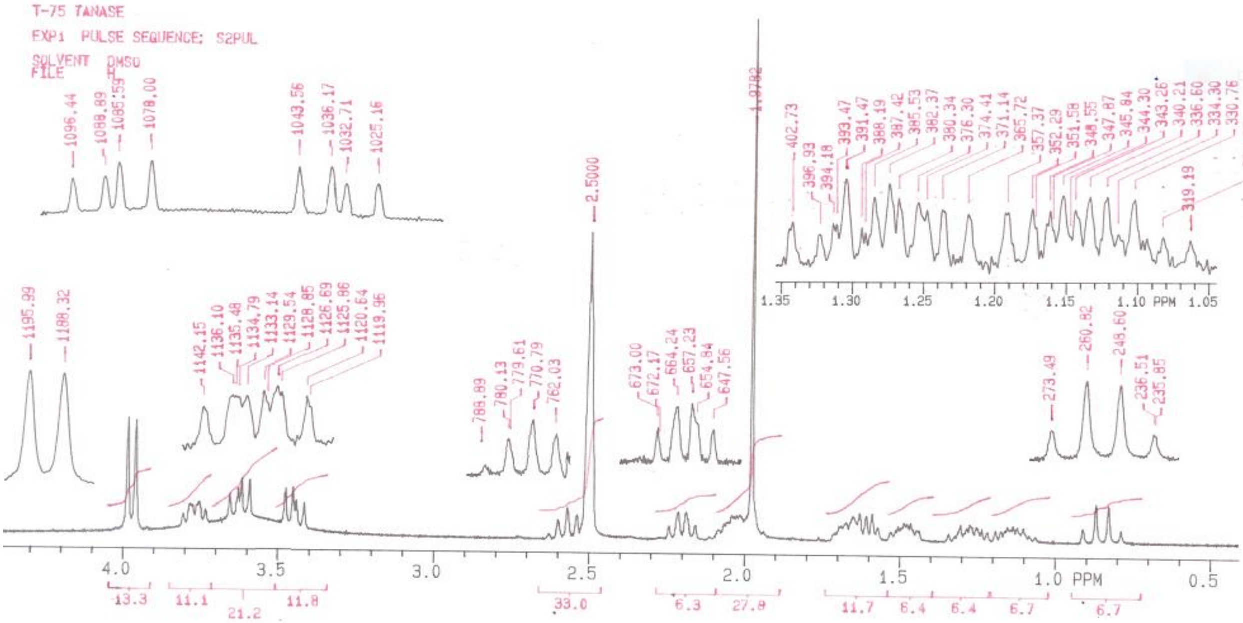

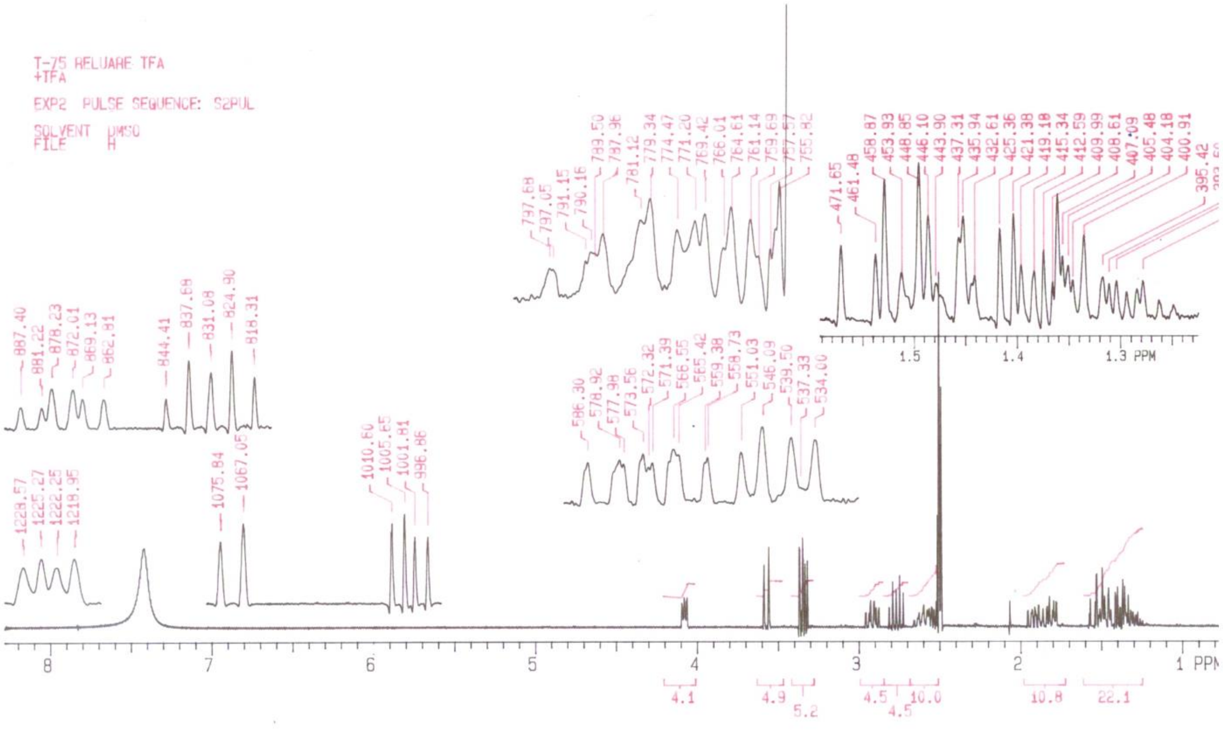

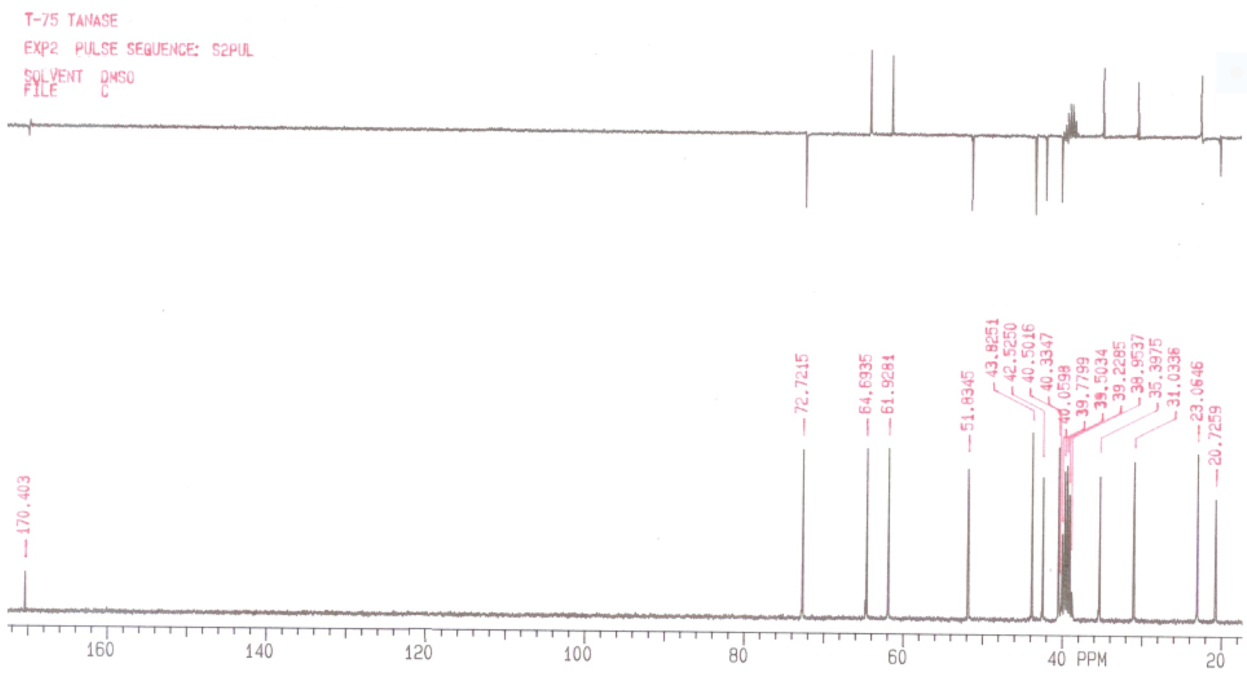
T-75 TANASE
EXP2 PULSE SEQUENCE: S2PUL
SOLVENT DMSO
FILE C
170.403
72.7215
64.6935
61.9281
51.8345
43.8251
42.5250
40.5016
40.3347
40.0598
39.7799
39.5034
39.2285
38.9537
35.3975
31.0336
23.0646
20.7259
160
140
120
100
80
60
40 PPM
20

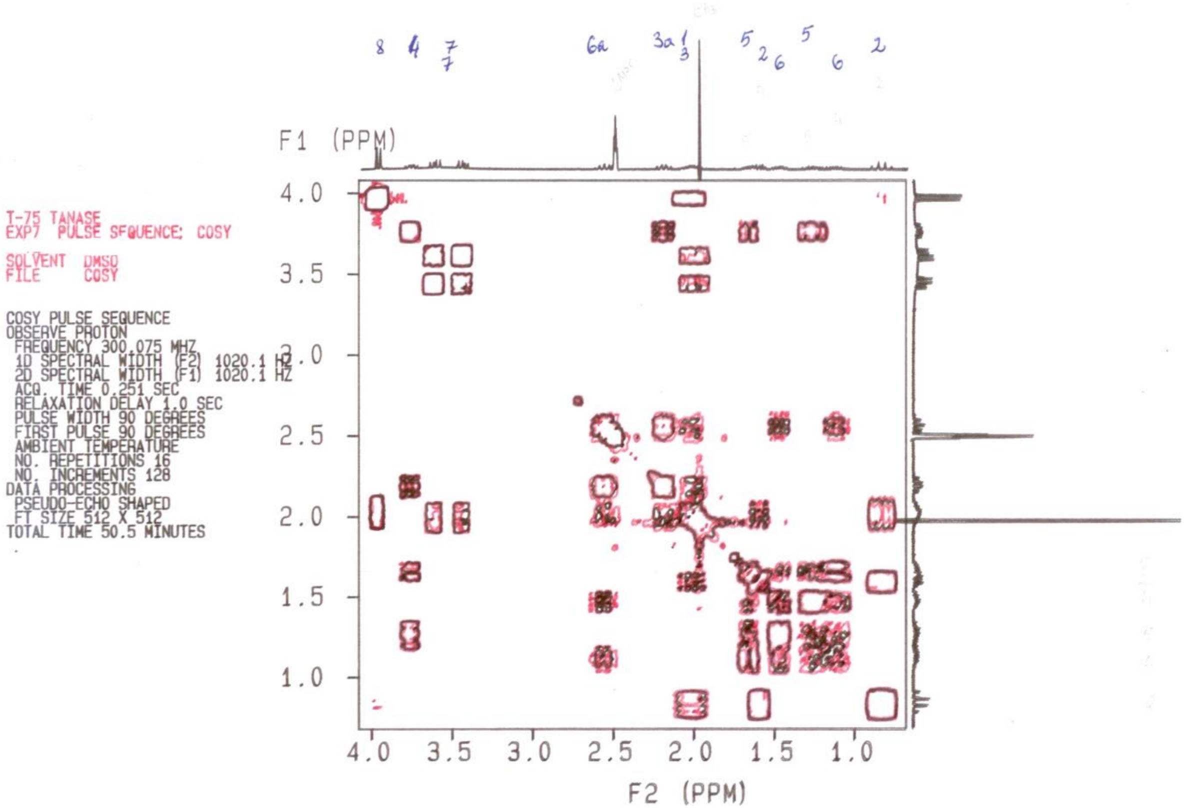
8 4 7 7 6a 3a 1 3 5 2 6 5 6 2
F1 (PPM)
T-75 TANASE
EXP7 PULSE SEQUENCE: COSY
SOLVENT DMSO
FILE COSY
COSY PULSE SEQUENCE
OBSERVE PROTON
FREQUENCY 300.075 MHZ
1D SPECTRAL WIDTH (F2) 1020.1 HZ
2D SPECTRAL WIDTH (F1) 1020.1 HZ
ACQ. TIME 0.251 SEC
RELAXATION DELAY 1.0 SEC
PULSE WIDTH 90 DEGREES
FIRST PULSE 90 DEGREES
AMBIENT TEMPERATURE
NO. REPETITIONS 16
NO. INCREMENTS 128
DATA PROCESSING
PSEUDO-ECHO SHAPED
FT SIZE 512 X 512
TOTAL TIME 50.5 MINUTES
4.0
3.5
3.0
2.5
2.0
1.5
1.0
F2 (PPM)

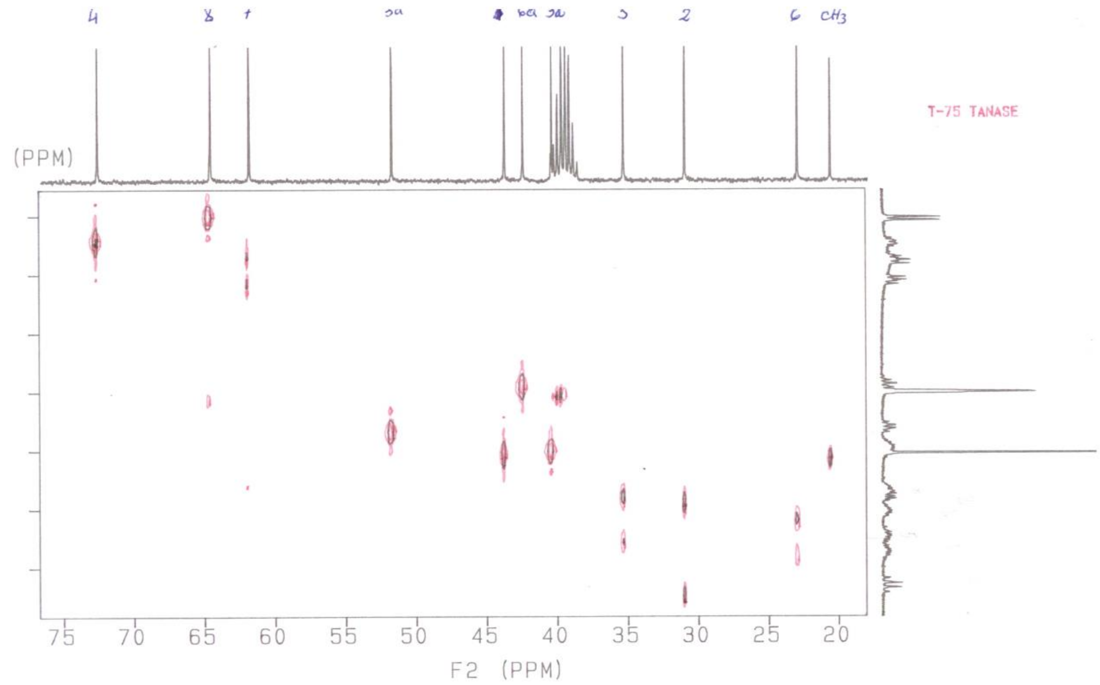

1.5. ^{1}H, ^{13}C and COSY spectra of the triolbenzoate obtained from symmetrical alcohol bisbenzoate **2a**

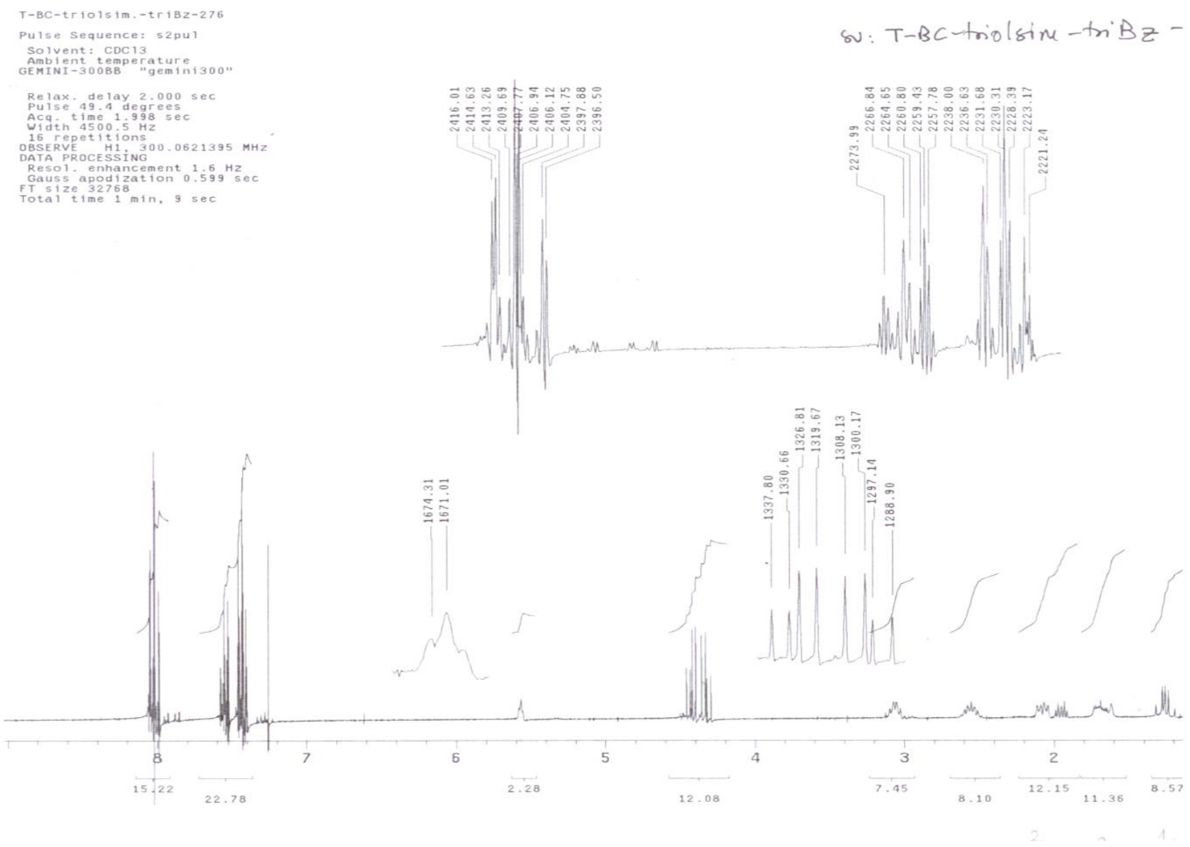

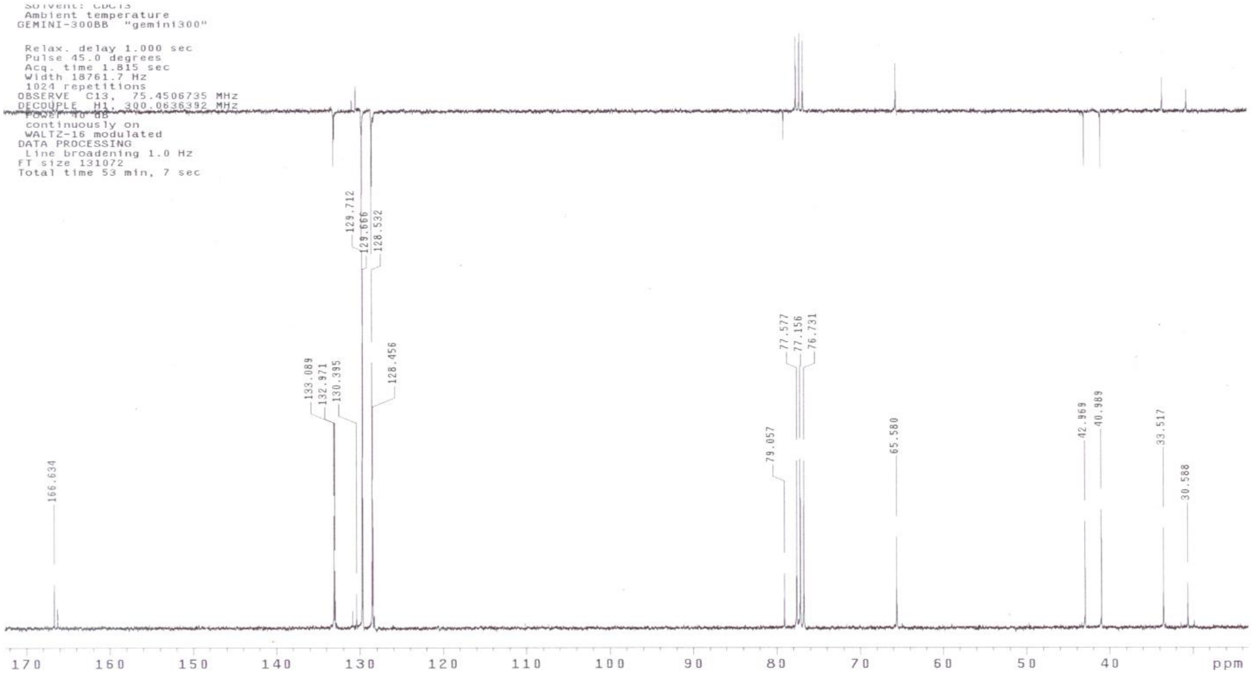
Solvent: CDCl3
Ambient temperature
GEMINI-300BB "gemini300"
Relax. delay 1.000 sec
Pulse 45.0 degrees
Acq. time 1.815 sec
Width 18761.7 Hz
1024 repetitions
OBSERVE C13, 75.4506735 MHz
DECOUPLE H1, 300.0636392 MHz
Power 40 dB
continuously on
WALTZ-16 modulated
DATA PROCESSING
Line broadening 1.0 Hz
FT size 131072
Total time 53 min, 7 sec
166.634
133.089
132.971
130.395
129.712
129.666
128.532
128.456
79.057
77.577
77.156
76.731
65.580
42.969
40.989
33.517
30.588
170 160 150 140 130 120 110 100 90 80 70 60 50 40 ppm

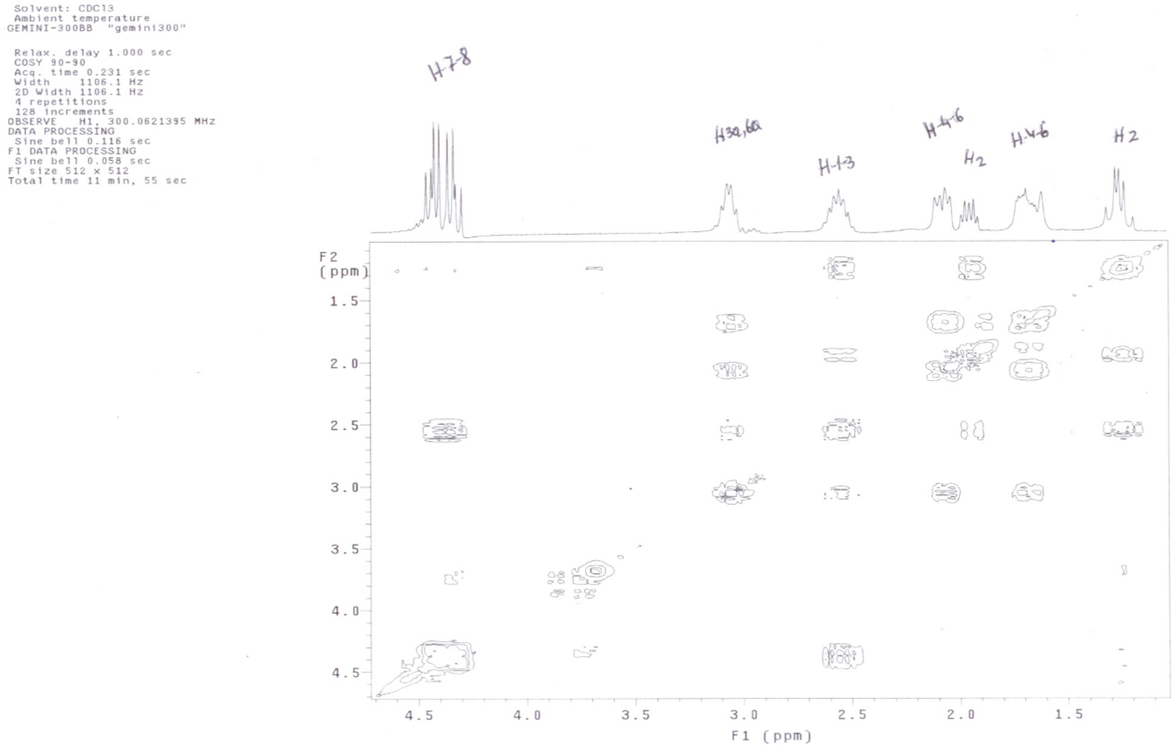
Solvent: CDCl3
Ambient temperature
GEMINI-300BB "gemini300"
Relax. delay 1.000 sec
COSY 90-90
Acq. time 0.231 sec
Width 1106.1 Hz
2D Width 1106.1 Hz
4 repetitions
128 increments
OBSERVE H1, 300.0621395 MHz
DATA PROCESSING
Sine bell 0.116 sec
F1 DATA PROCESSING
Sine bell 0.058 sec
FT size 512 x 512
Total time 11 min, 55 sec
H7-8
H3a,6a
H-1-3
H4-6
H2
H4-6
H2
F2 (ppm)
1.5
2.0
2.5
3.0
3.5
4.0
4.5
4.5 4.0 3.5 3.0 2.5 2.0 1.5
F1 (ppm)

1.6. ^{1}H, ^{13}C, COSY and HETCOR spectra of the triolbenzoate **3a-TriBz** obtained from unsymmetrical alchohol bisbenzoate **3a**

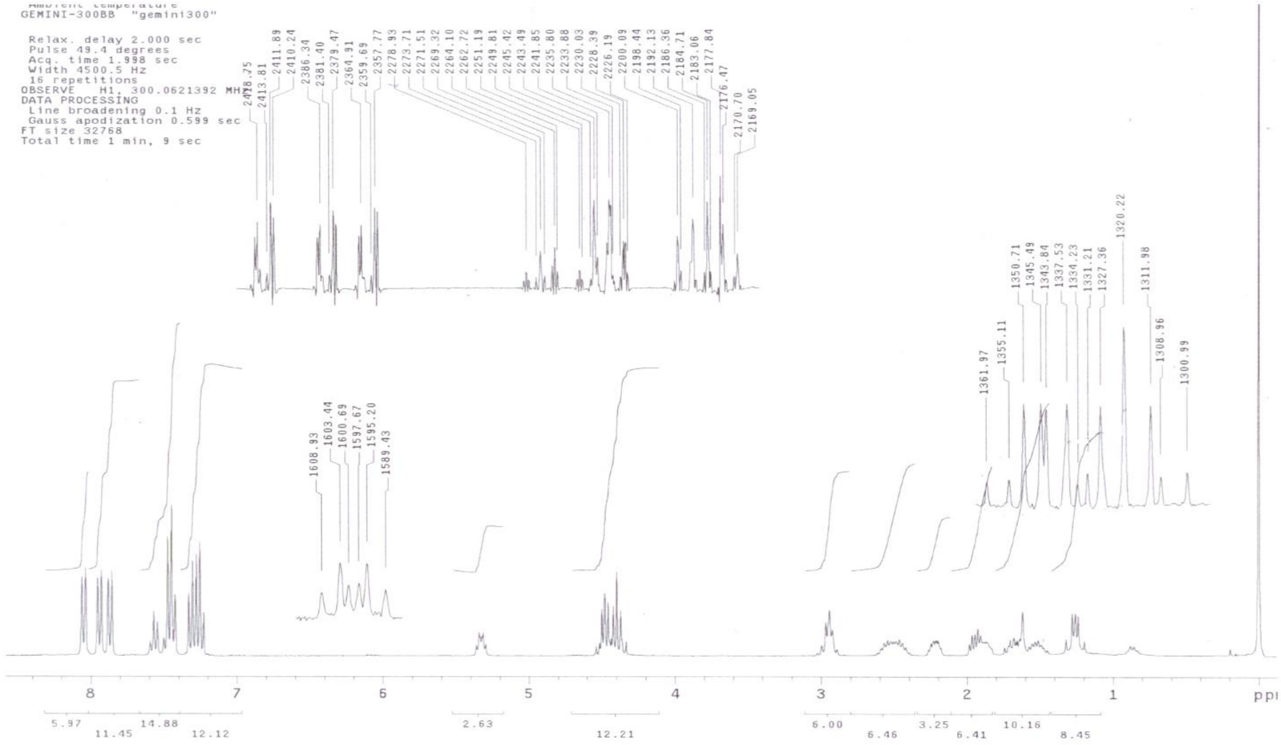

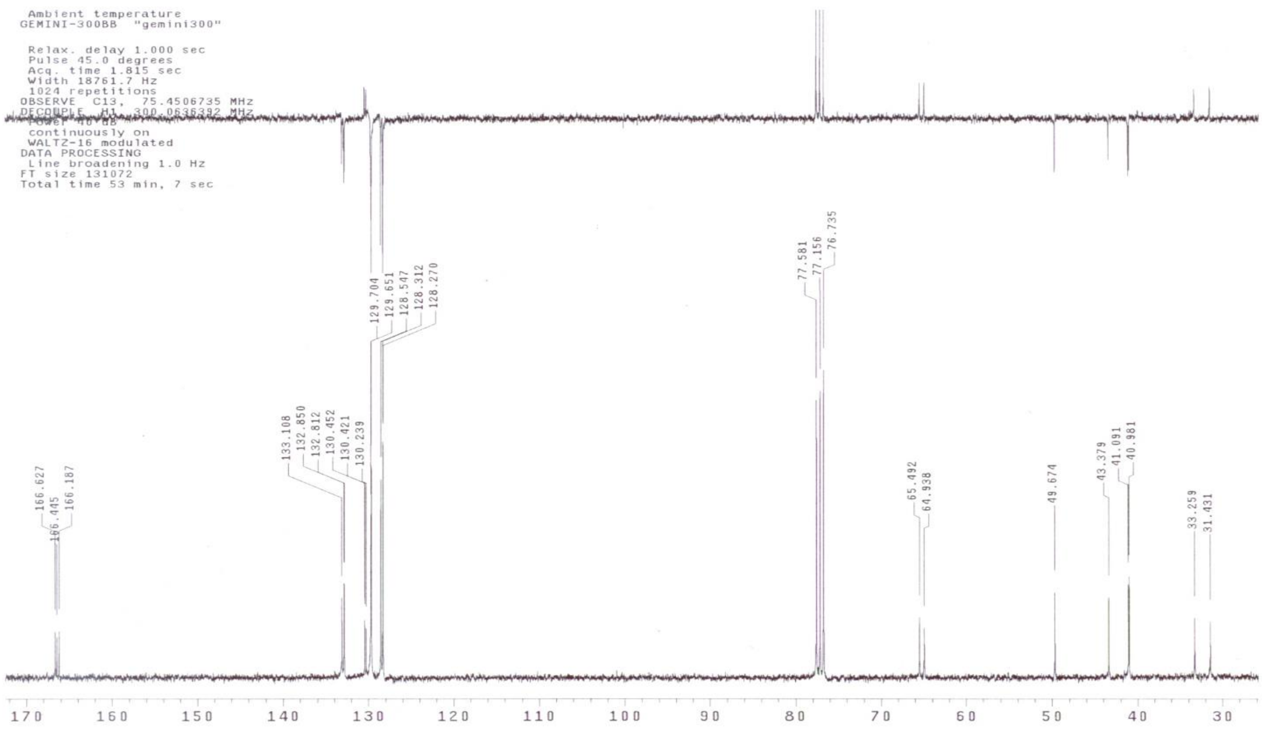

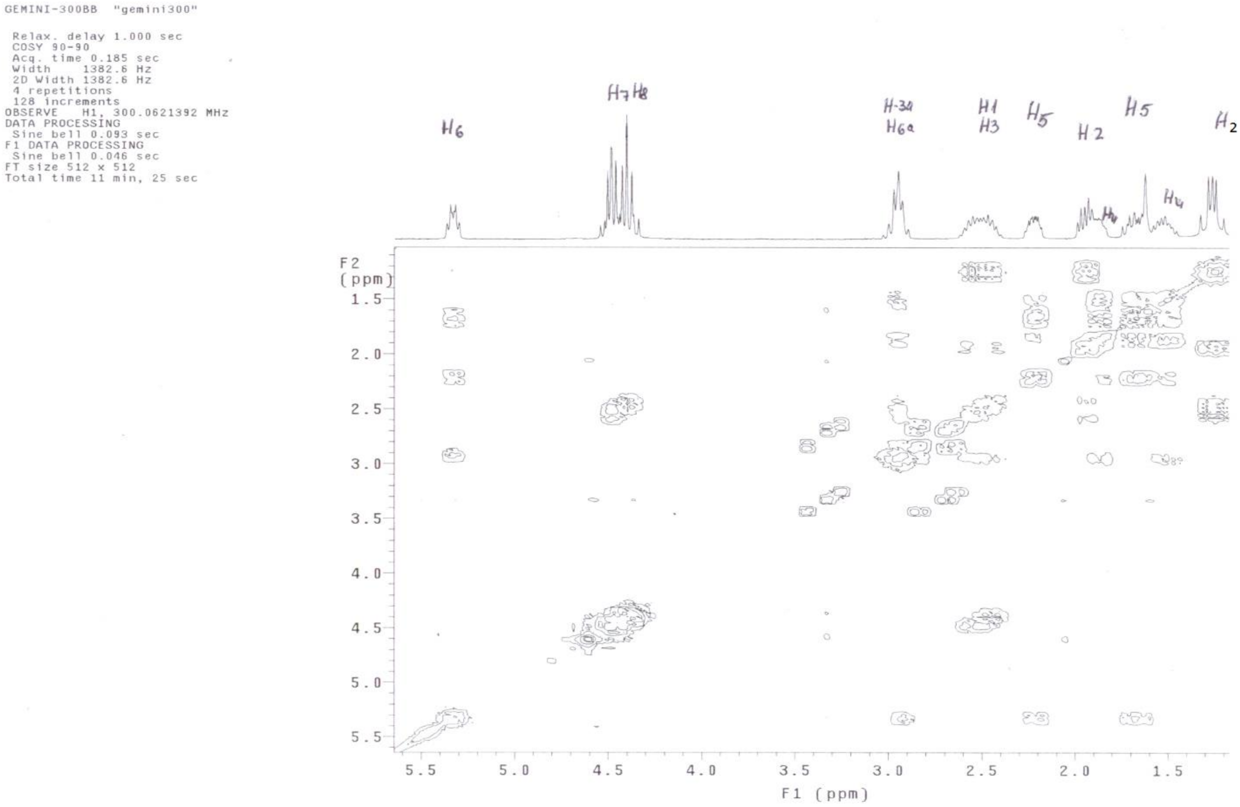
GEMINI-300BB "gemini300"
Relax. delay 1.000 sec
COSY 90-90
Acq. time 0.185 sec
Width 1382.6 Hz
2D Width 1382.6 Hz
4 repetitions
128 increments
OBSERVE H1, 300.0621392 MHz
DATA PROCESSING
Sine bell 0.093 sec
F1 DATA PROCESSING
Sine bell 0.046 sec
FT size 512 x 512
Total time 11 min, 25 sec
H6
H7 H8
H-3a
H6a
H1
H3
H5
H2
H5
H4
H2
F2 (ppm)
F1 (ppm)

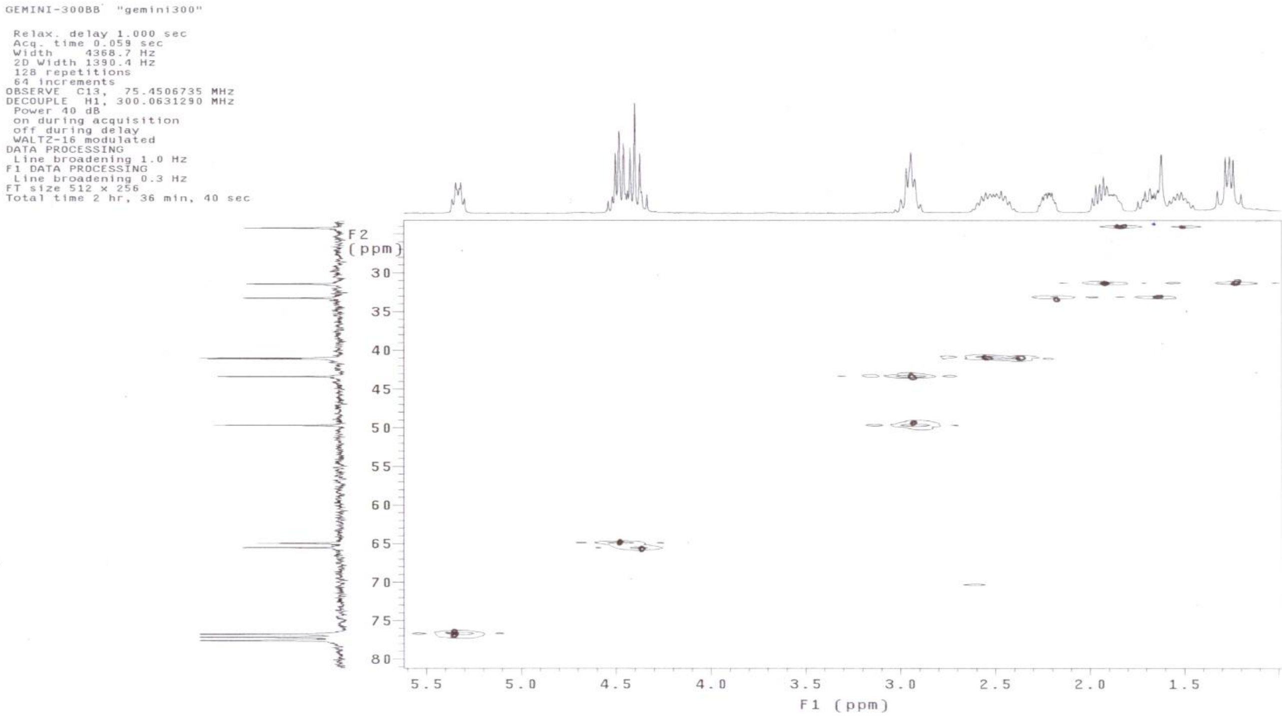
GEMINI-300BB "gemini300"
Relax. delay 1.000 sec
Acq. time 0.059 sec
Width 4368.7 Hz
2D Width 1390.4 Hz
128 repetitions
64 increments
OBSERVE C13, 75.4506735 MHz
DECOUPLE H1, 300.0631290 MHz
Power 40 dB
on during acquisition
off during delay
WALTZ-16 modulated
DATA PROCESSING
Line broadening 1.0 Hz
F1 DATA PROCESSING
Line broadening 0.3 Hz
FT size 512 x 256
Total time 2 hr, 36 min, 40 sec
F2 (ppm)
F1 (ppm)

1.7. ^{1}H, and ^{13}C spectra of the unsymmetric triol trisbenzoate compound **3a-TriBz** obtained from **4a**

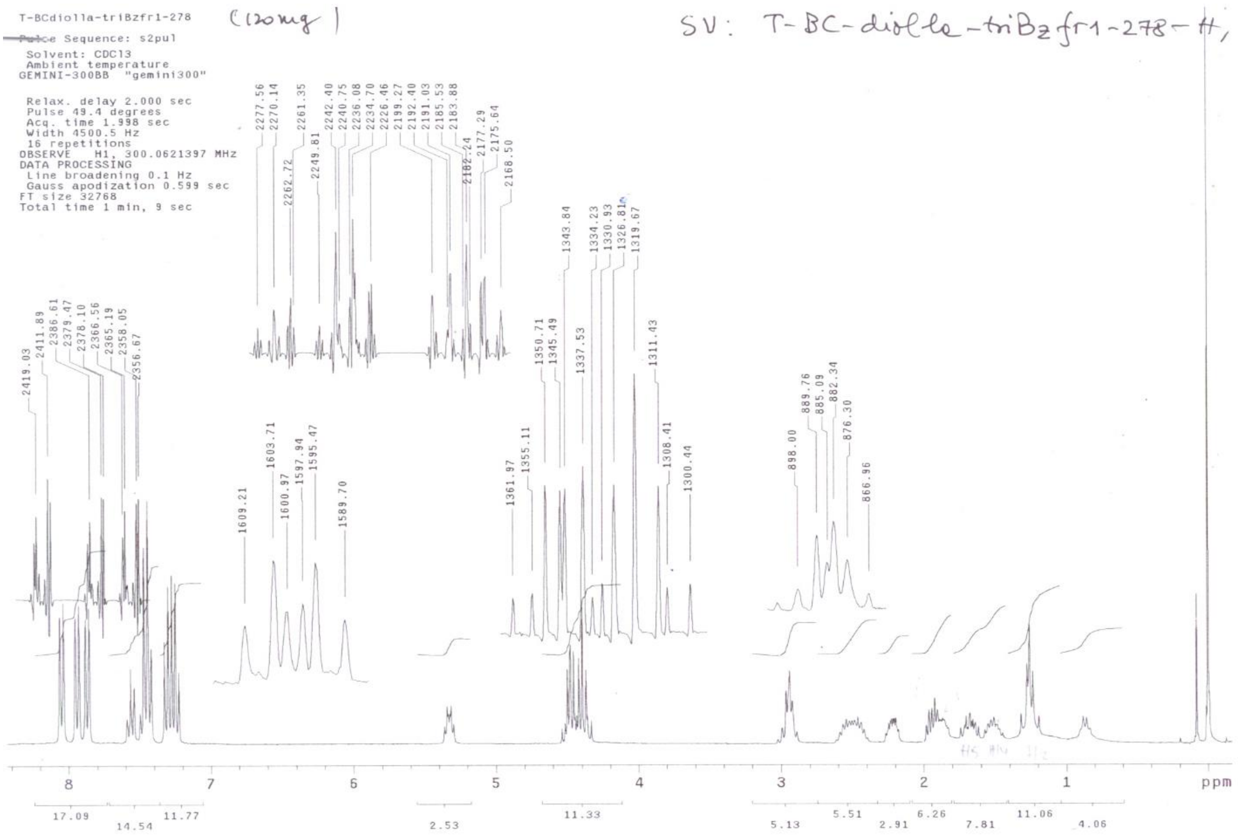

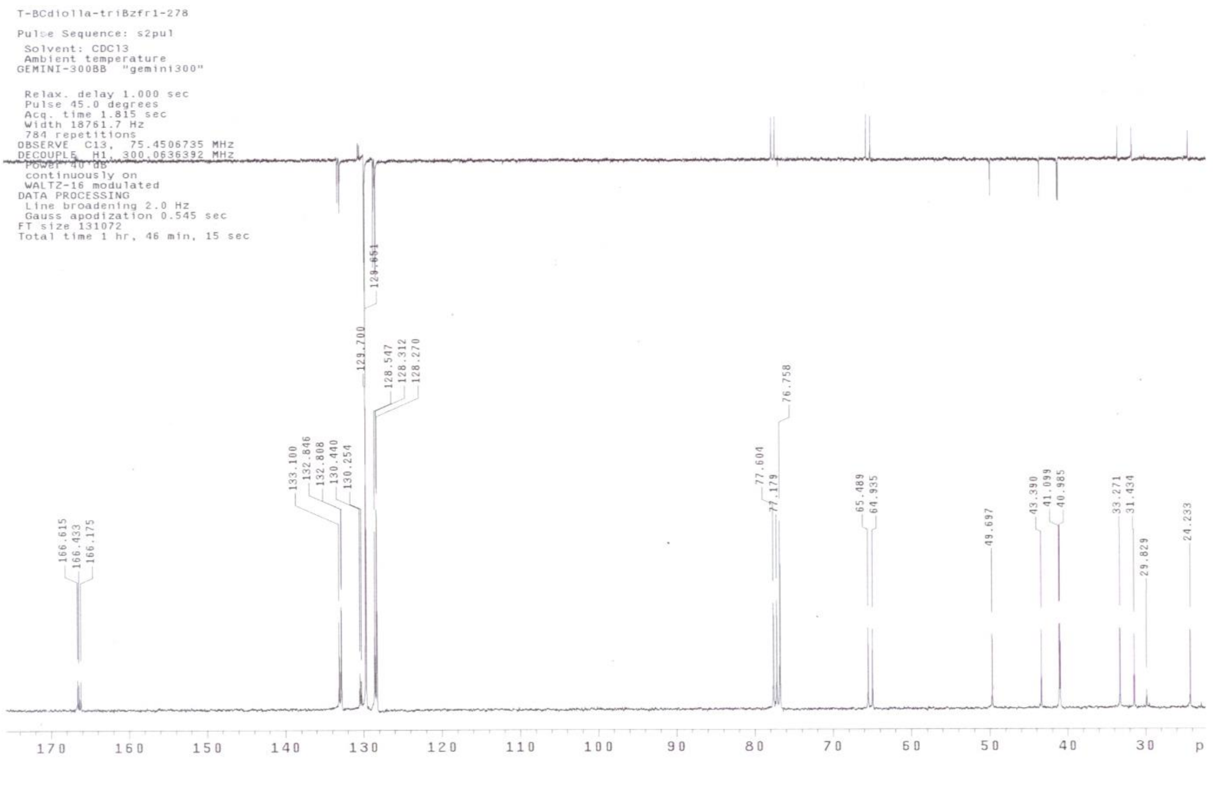

1.8. ^{1}H, ^{1}H+TFA and ^{13}C spectra of the symmetric triol compound **8**

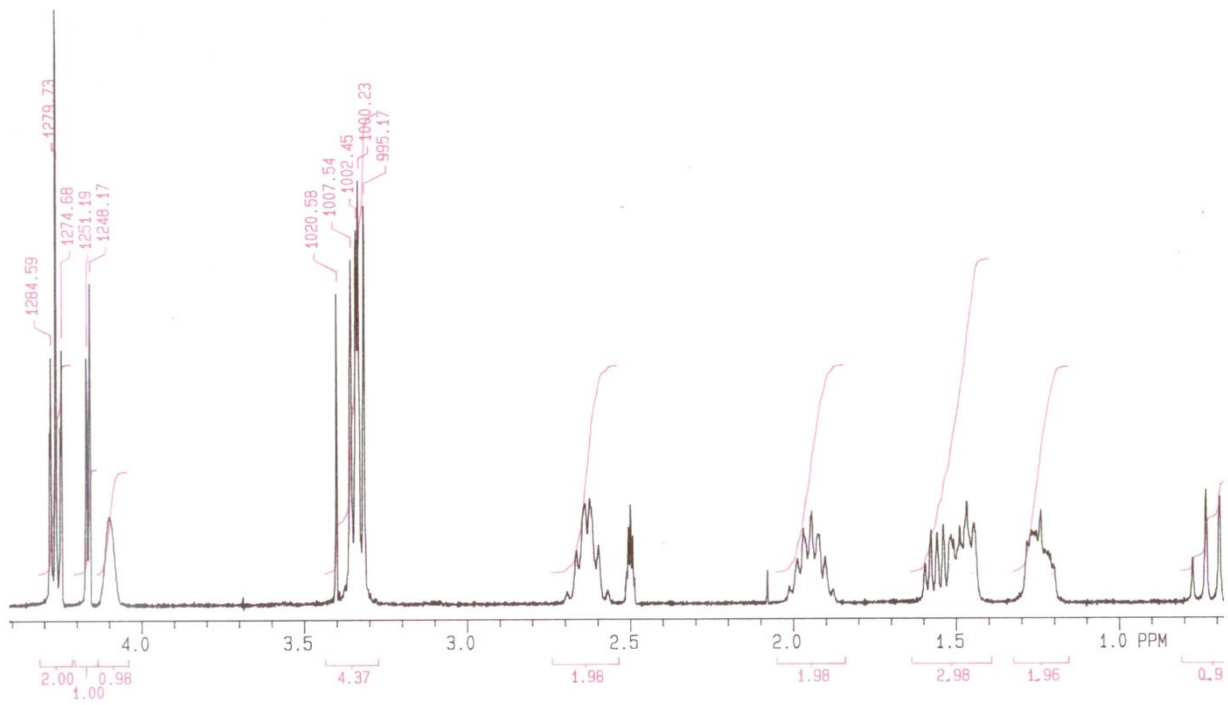

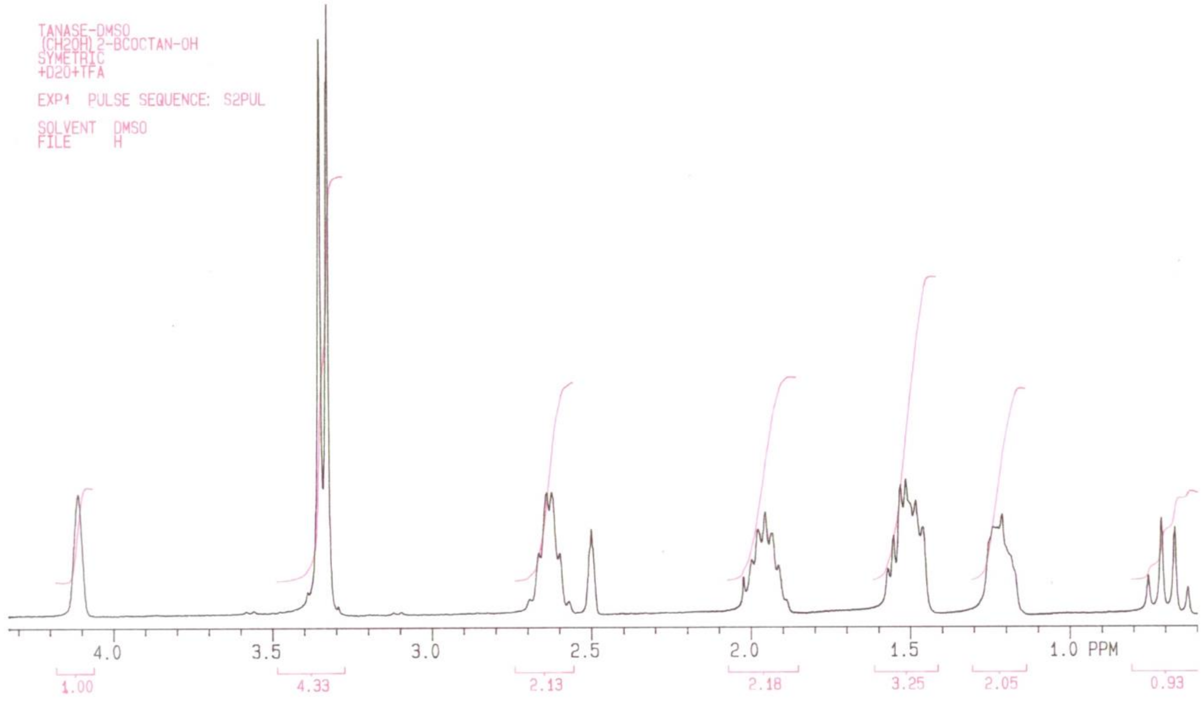

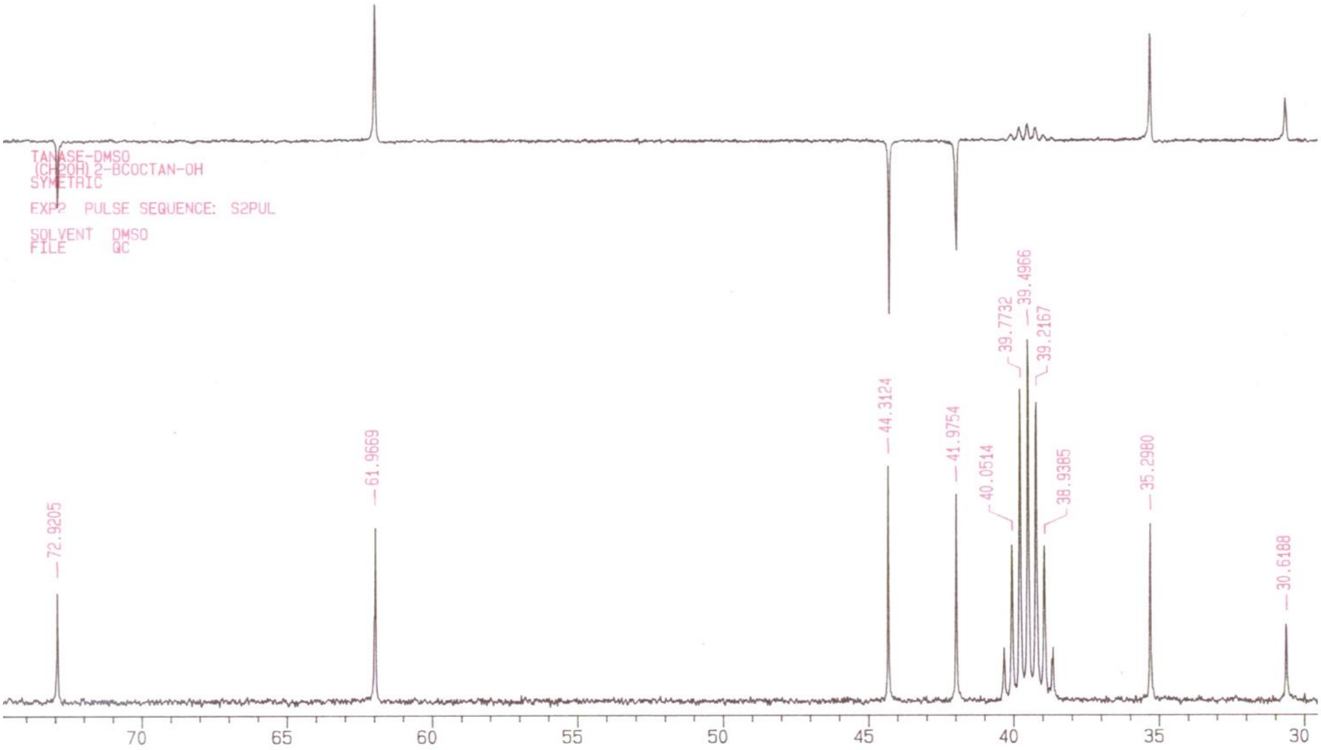

1.9. ^{1}H, ^{13}C and HETCOR spectra in $CDCl_3$ of compound **5a**

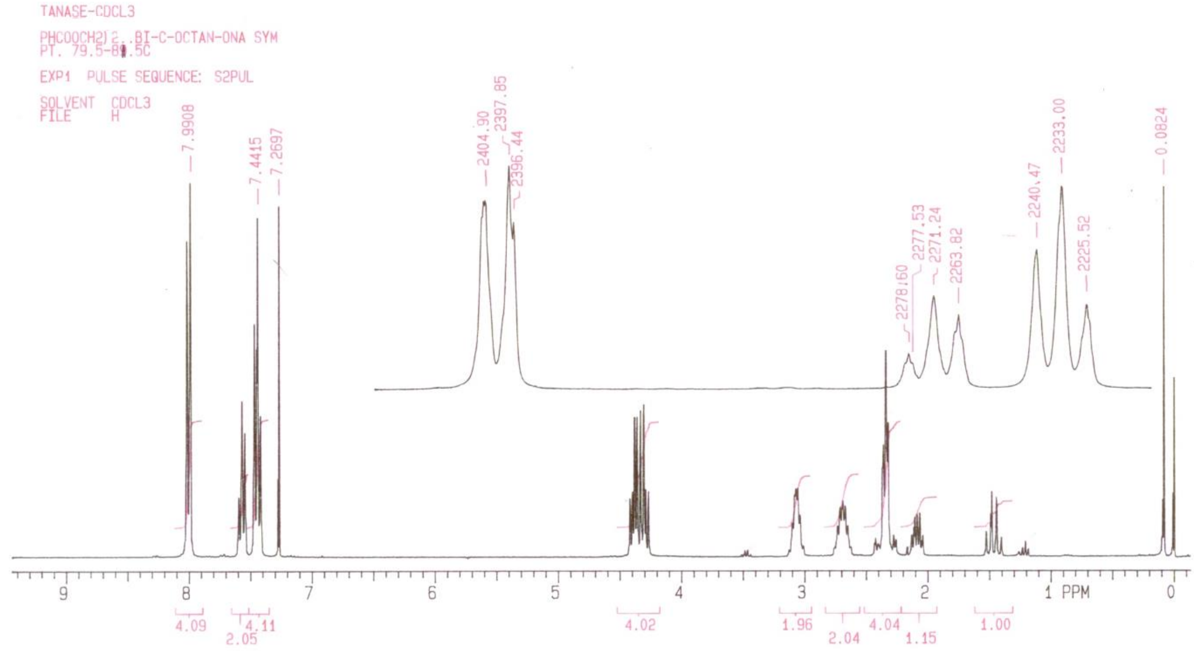

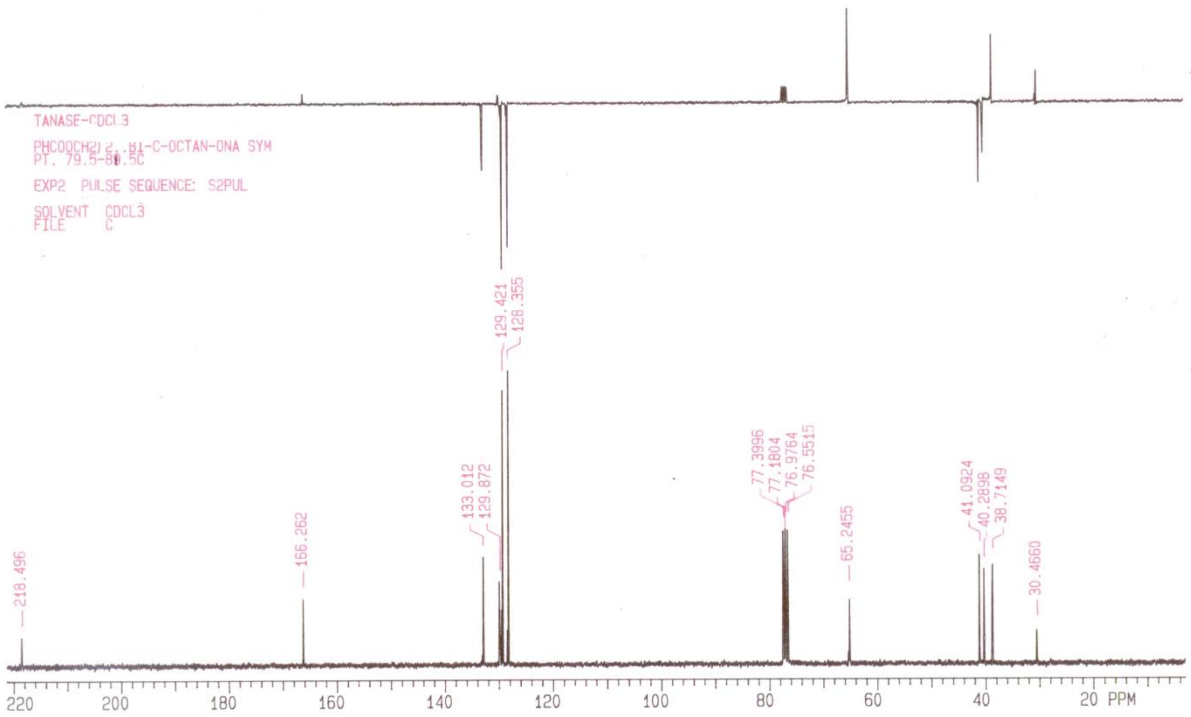
TANASE-CDCL3
PHCOOCH2)2..BI-C-OCTAN-ONA SYM
PT. 79.5-80.5C
EXP2 PULSE SEQUENCE: S2PUL
SOLVENT CDCL3
FILE C
218.496
166.262
133.012
129.872
129.421
128.355
77.3996
77.1804
76.9764
76.5515
65.2455
41.0924
40.2898
38.7149
30.4660
220 200 180 160 140 120 100 80 60 40 20 PPM

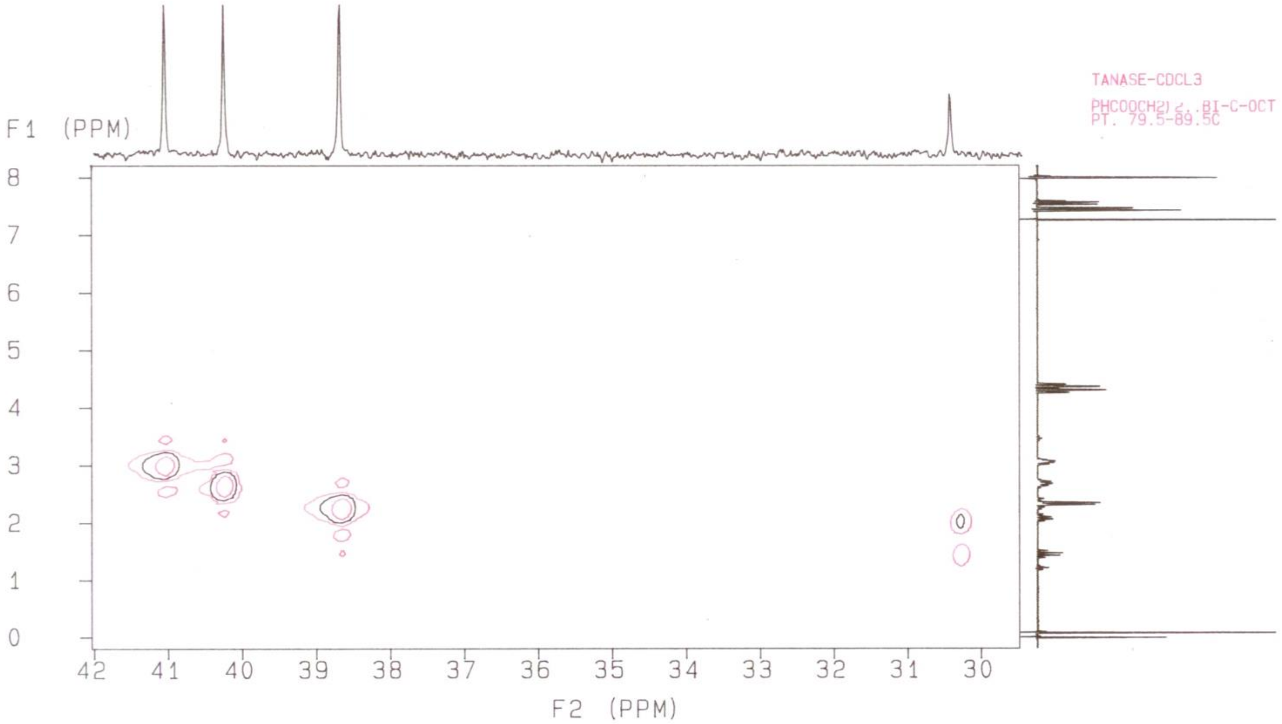
TANASE-CDCL3
PHCOOCH2)2..BI-C-OCT
PT. 79.5-89.5C
F1 (PPM)
8 7 6 5 4 3 2 1 0
42 41 40 39 38 37 36 35 34 33 32 31 30
F2 (PPM)

1.10. ^{1}H, and ^{13}C spectra in $CDCl_3$ of compound **5c**

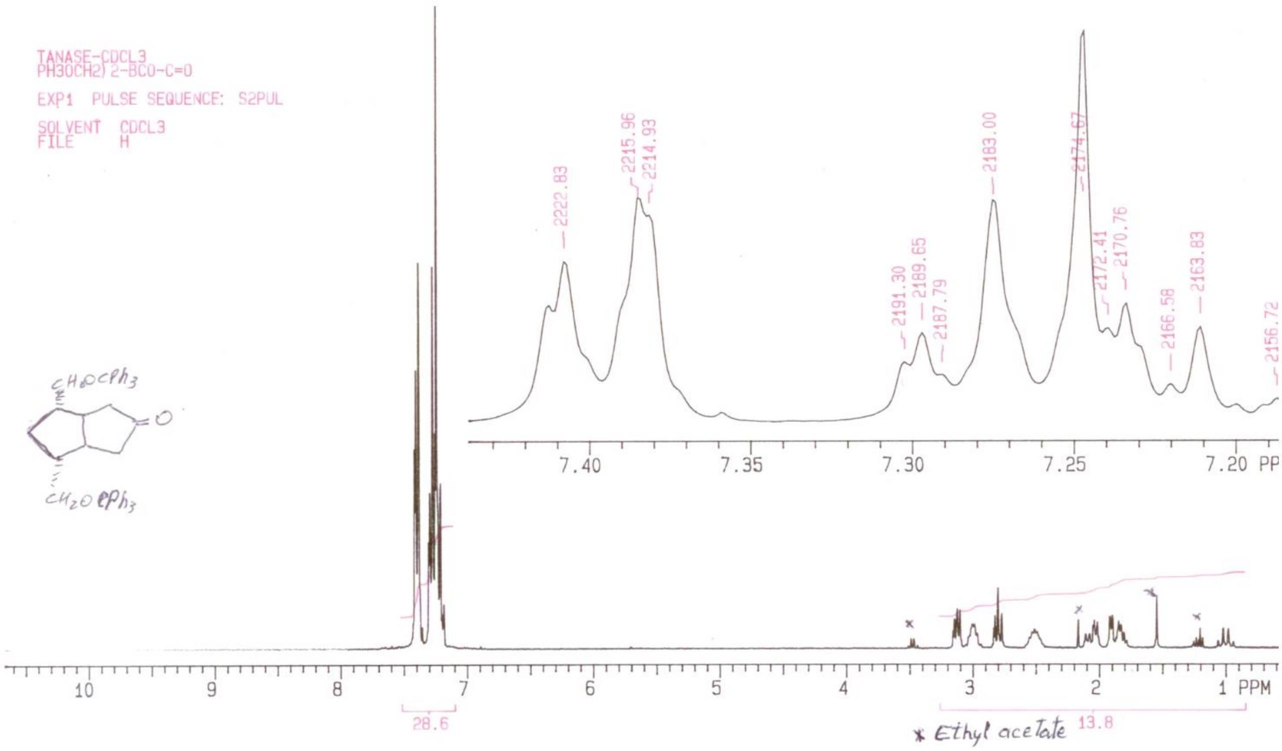

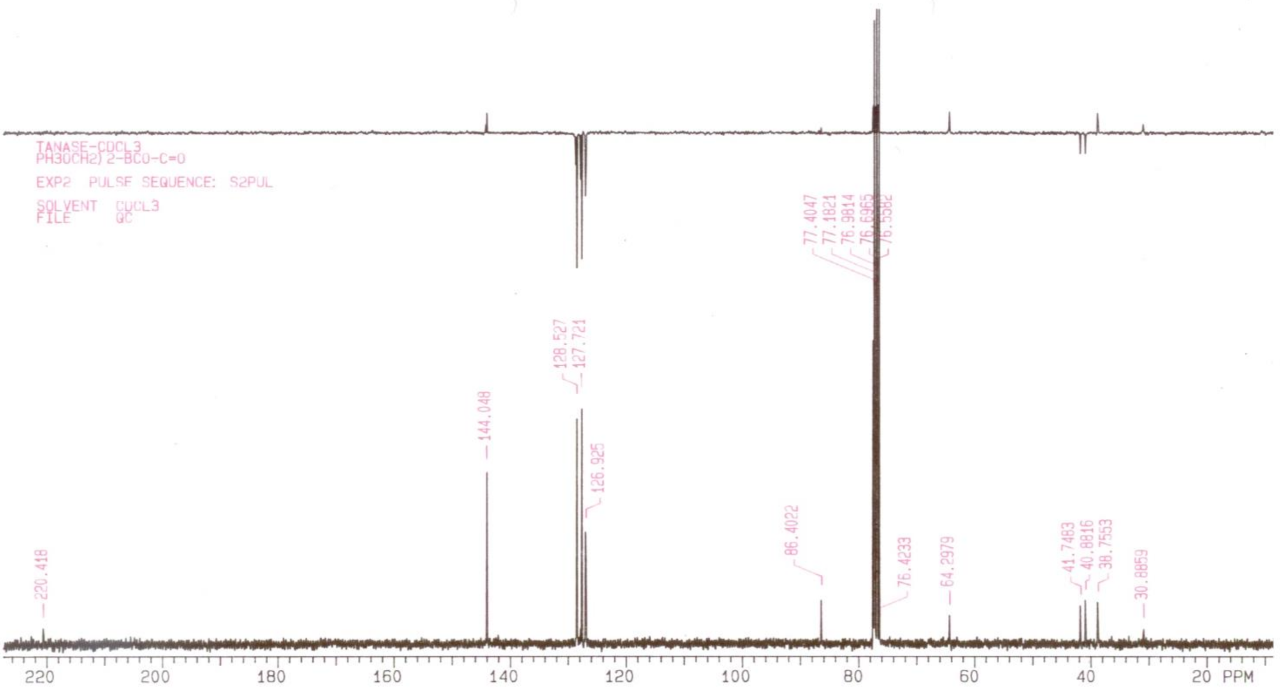

1.11. ^{1}H, ^{13}C, COSY and HETCOR spectra in $CDCl_3$ of compound **6a**

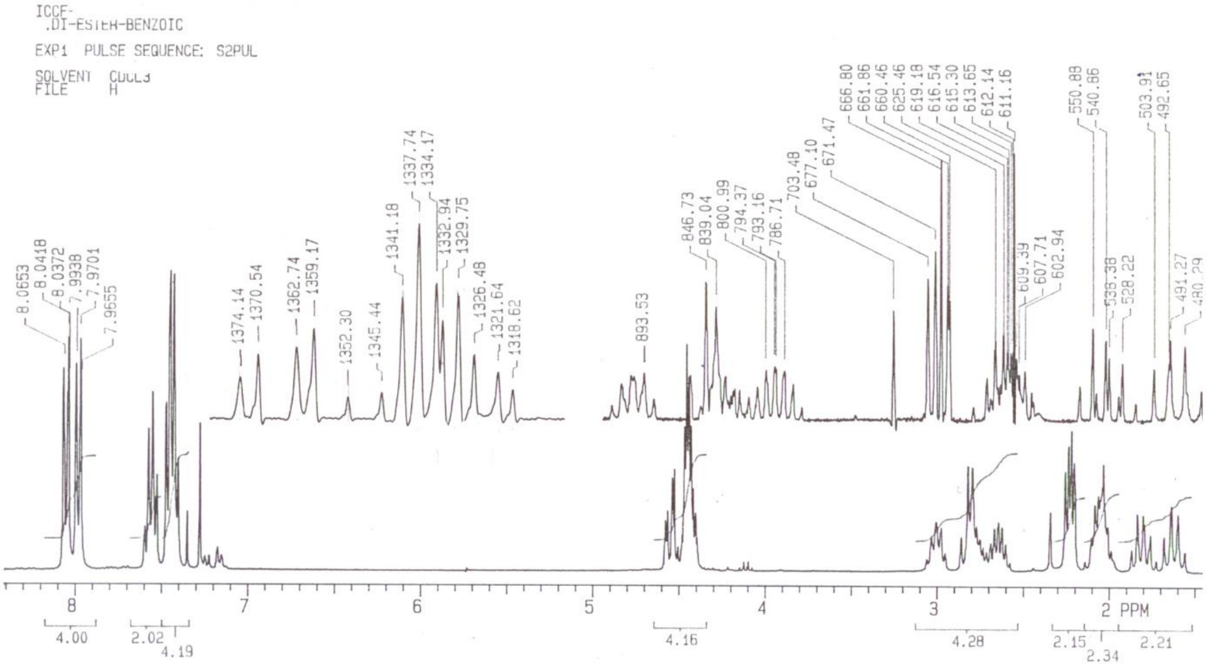

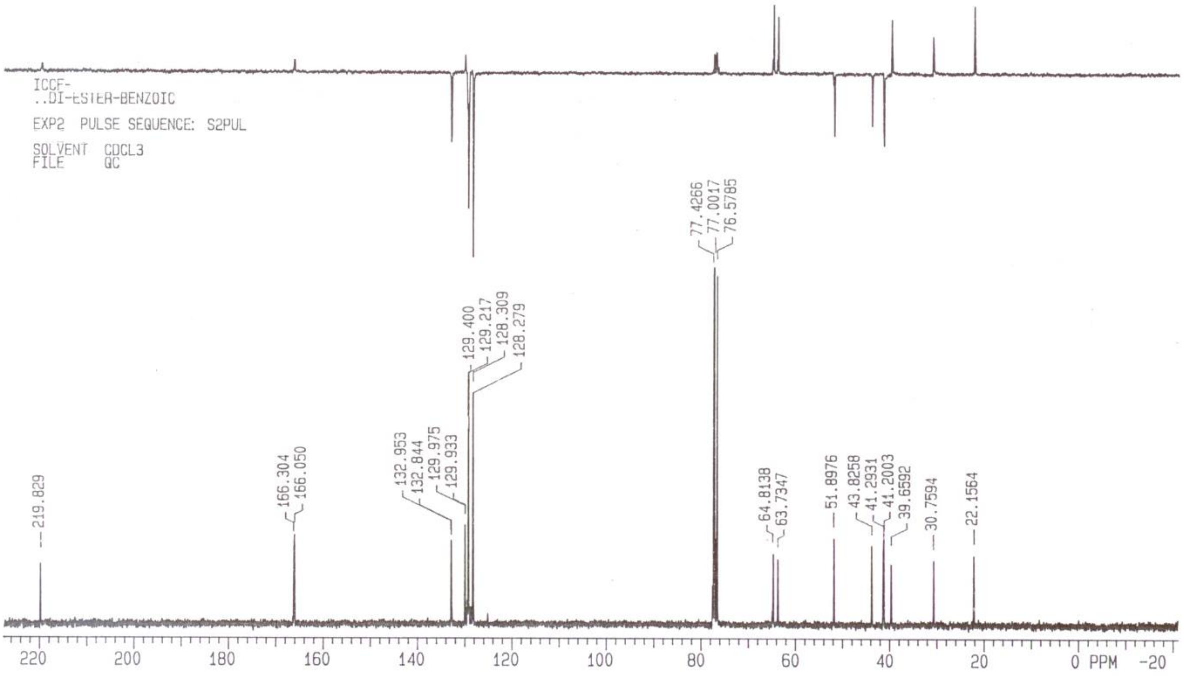

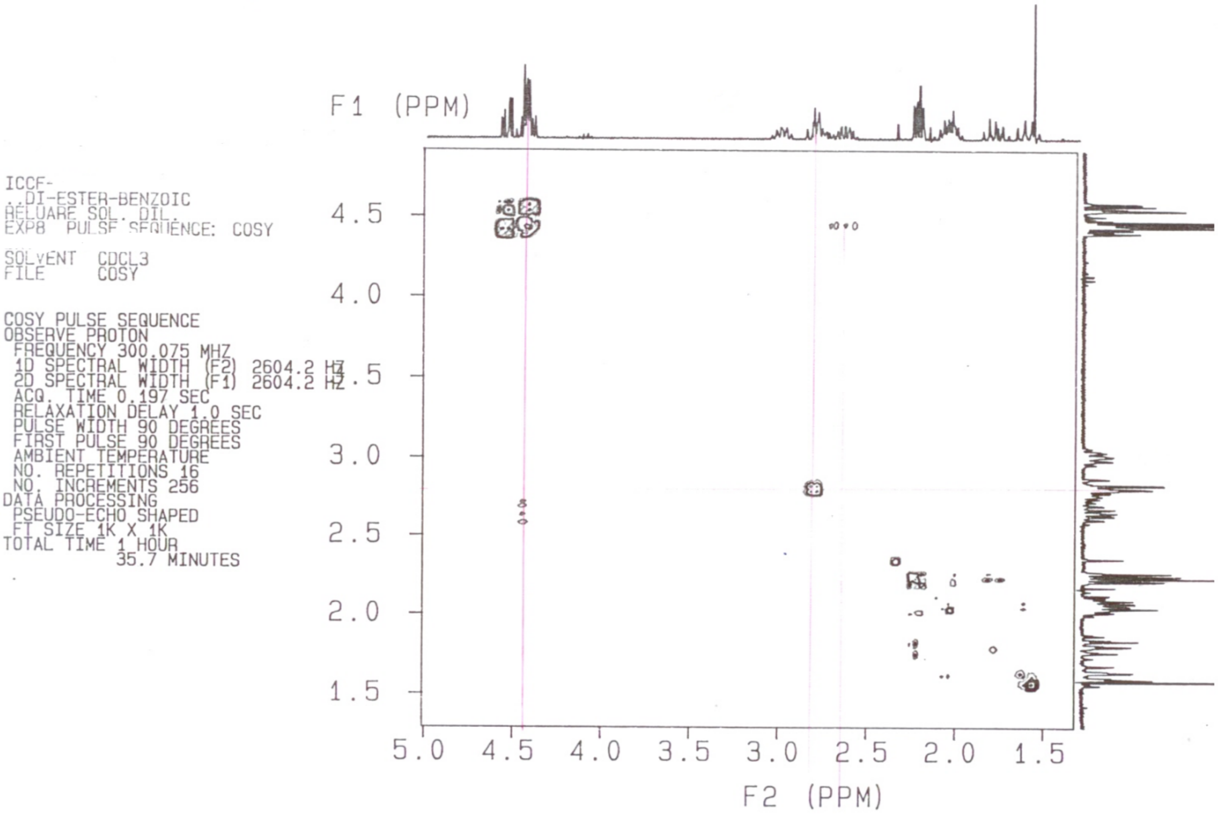
ICCF-
..DI-ESTER-BENZOIC
RELUARE SOL. DIL.
EXP8 PULSE SEQUENCE: COSY
SOLVENT CDCL3
FILE COSY
COSY PULSE SEQUENCE
OBSERVE PROTON
FREQUENCY 300.075 MHZ
1D SPECTRAL WIDTH (F2) 2604.2 HZ
2D SPECTRAL WIDTH (F1) 2604.2 HZ
ACQ. TIME 0.197 SEC
RELAXATION DELAY 1.0 SEC
PULSE WIDTH 90 DEGREES
FIRST PULSE 90 DEGREES
AMBIENT TEMPERATURE
NO. REPETITIONS 16
NO. INCREMENTS 256
DATA PROCESSING
PSEUDO-ECHO SHAPED
FT SIZE 1K X 1K
TOTAL TIME 1 HOUR
35.7 MINUTES
F1 (PPM)
4.5
4.0
3.5
3.0
2.5
2.0
1.5
5.0 4.5 4.0 3.5 3.0 2.5 2.0 1.5
F2 (PPM)

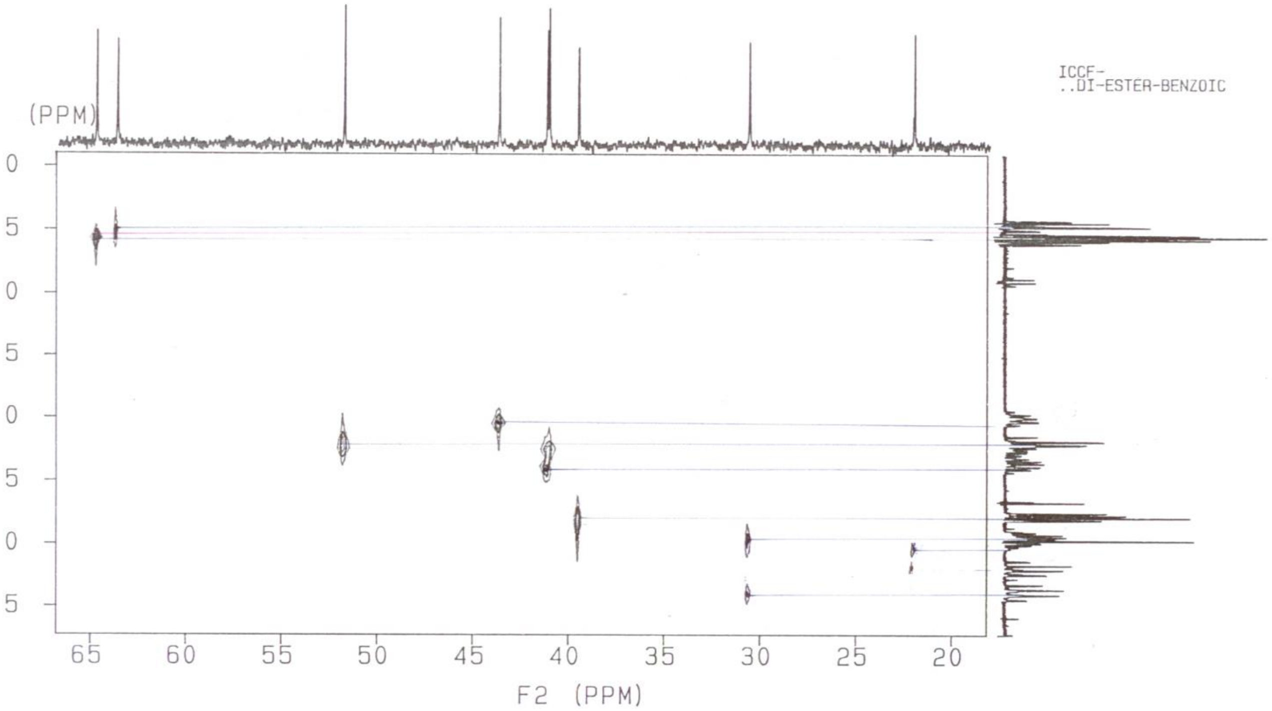
ICCF-
..DI-ESTER-BENZOIC
(PPM)
0
5
0
5
0
5
0
5
65 60 55 50 45 40 35 30 25 20
F2 (PPM)

1.12. ^{1}H, ^{13}C, COSY and HETCOR Spectra in DMSO of the di-Bz-ethylene ketal compound **11**

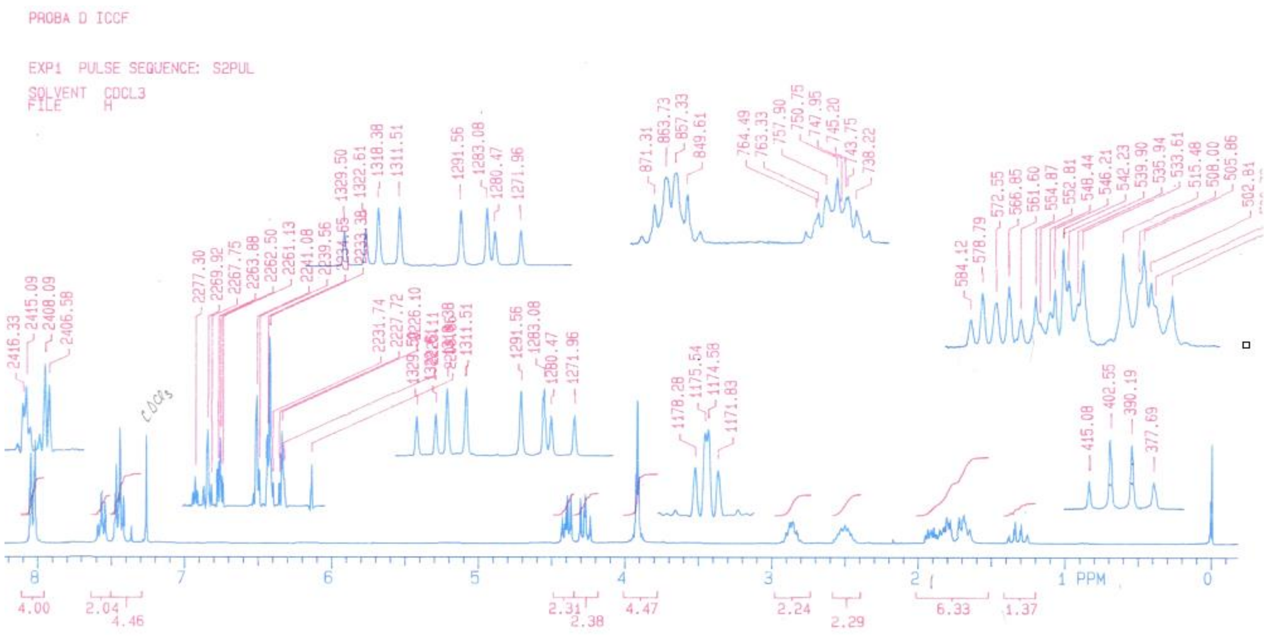

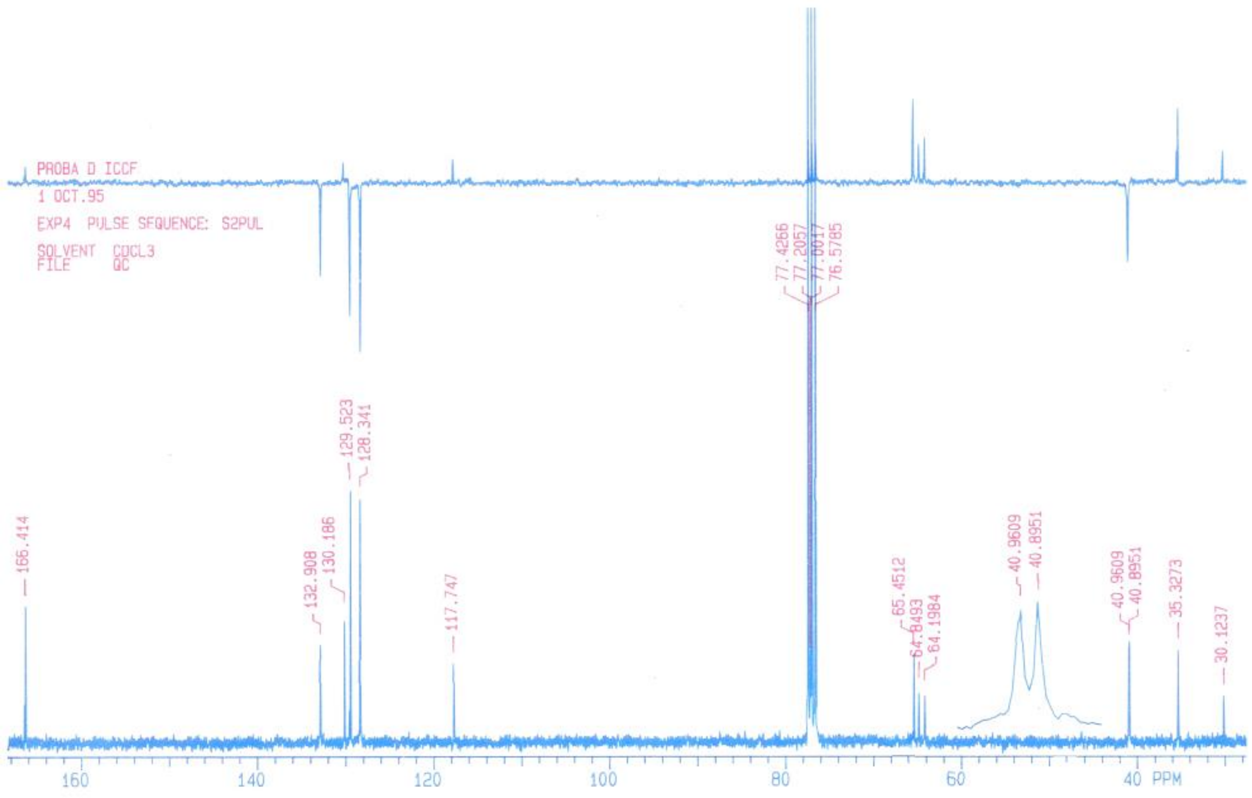

PROBA D ICCF

EXP5 PULSE SEQUENCE: COSY

SOLVENT CDCL3
FILE COSY

COSY PULSE SEQUENCE
OBSERVE PROTON
FREQUENCY 300.075 MHZ
1D SPECTRAL WIDTH (F2) 1112.2 HZ
2D SPECTRAL WIDTH (F1) 1112.2 HZ
ACQ. TIME 0.23 SEC
RELAXATION DELAY 1.0 SEC
PULSE WIDTH 90 DEGREES
FIRST PULSE 90 DEGREES
AMBIENT TEMPERATURE
NO. REPETITIONS 16
NO. INCREMENTS 64
DATA PROCESSING
PSEUDO-ECHO SHAPED
FT SIZE 512 X 512
TOTAL TIME 24.2 MINUTES

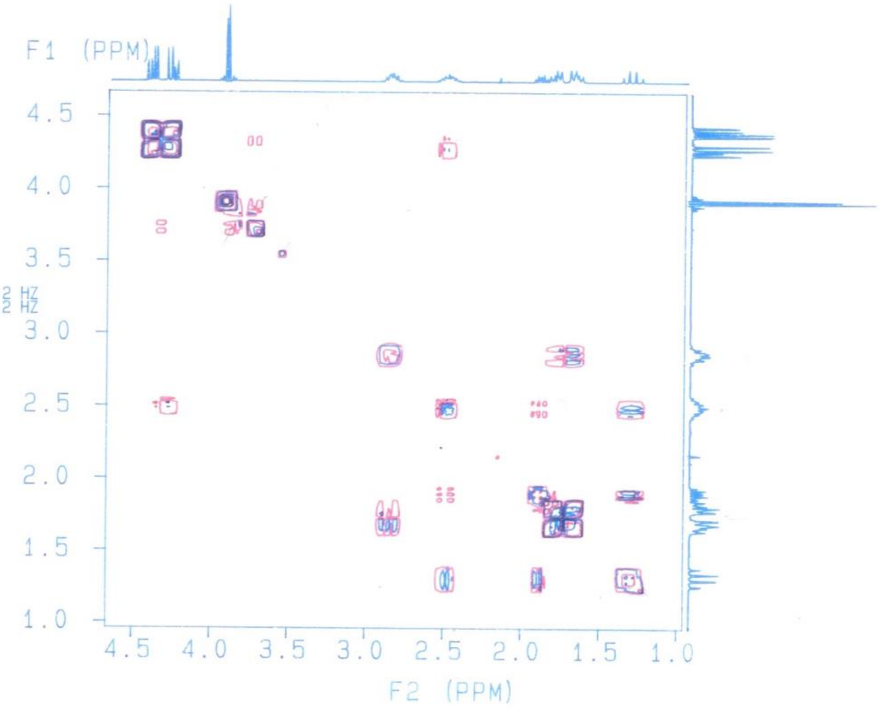

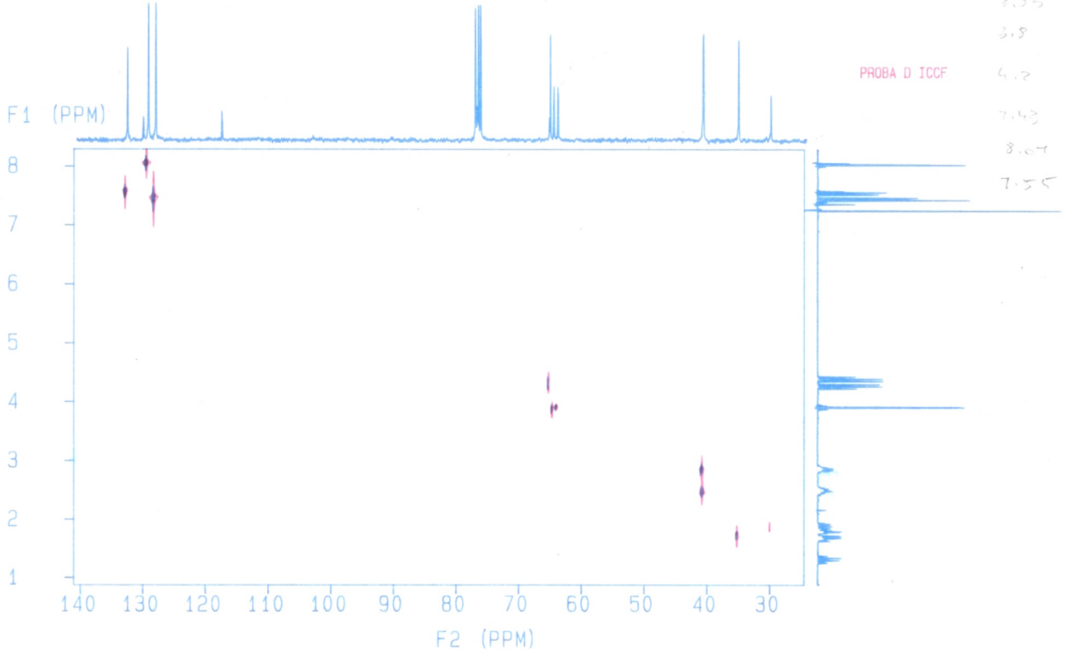

PROBA D ICCF

1.31 –	29.2
1.65 –	34.5
40.2 –	2.39<br>2.78
3.53 –	63.5
3.8	64.2
4.2	64.7
7.43	127.88
8.04	129.41
7.55	132.7

1.13. ^{1}H, ^{13}C, COSY and HETCOR spectra in DMSO of the ethylene ketal compound **13**

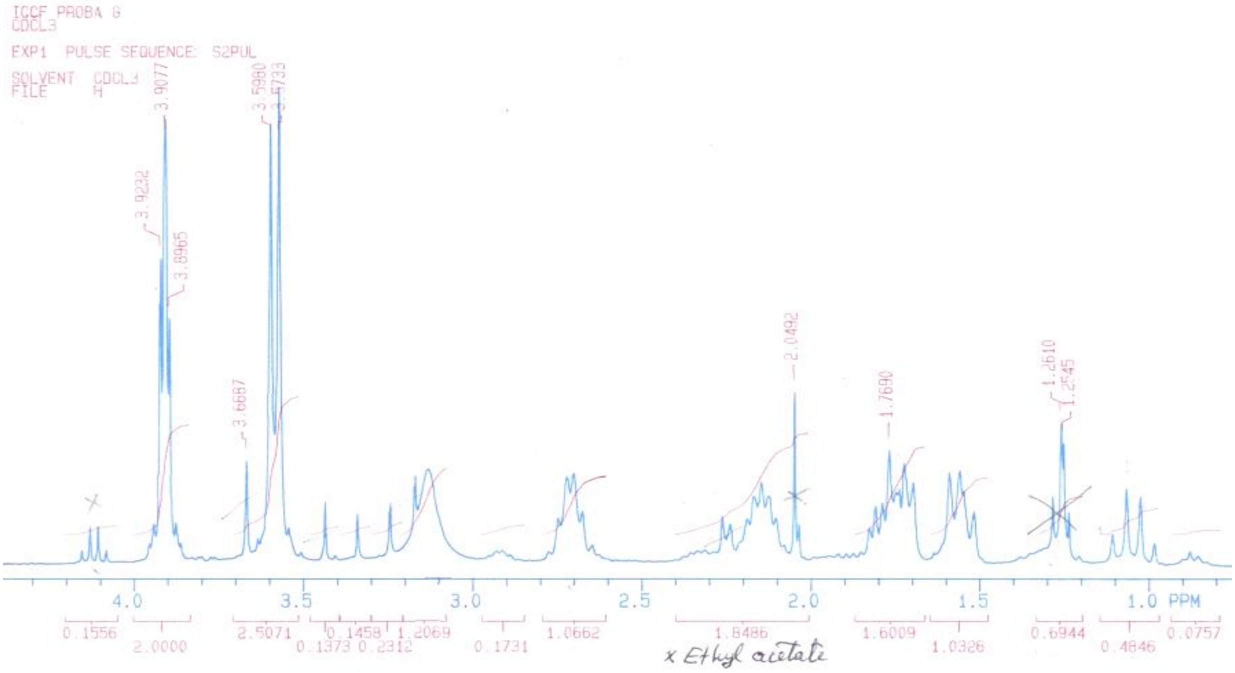

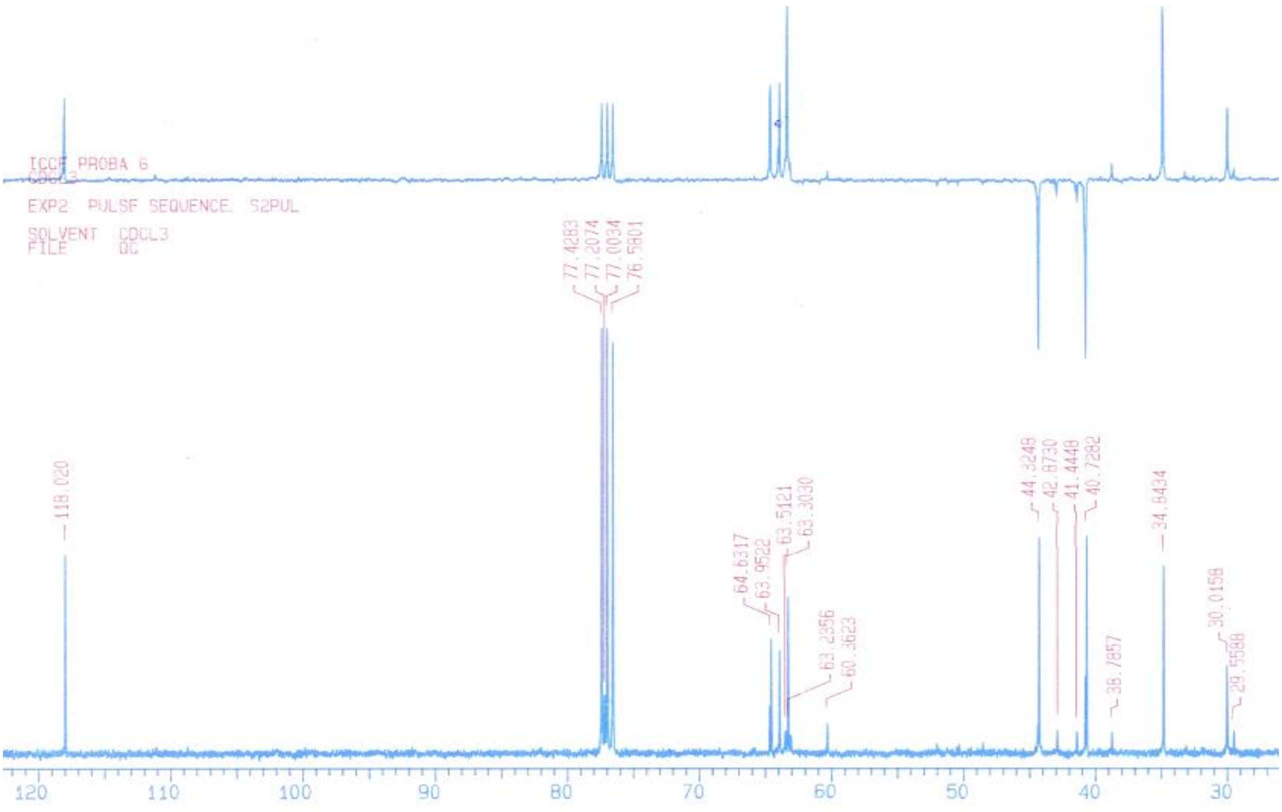

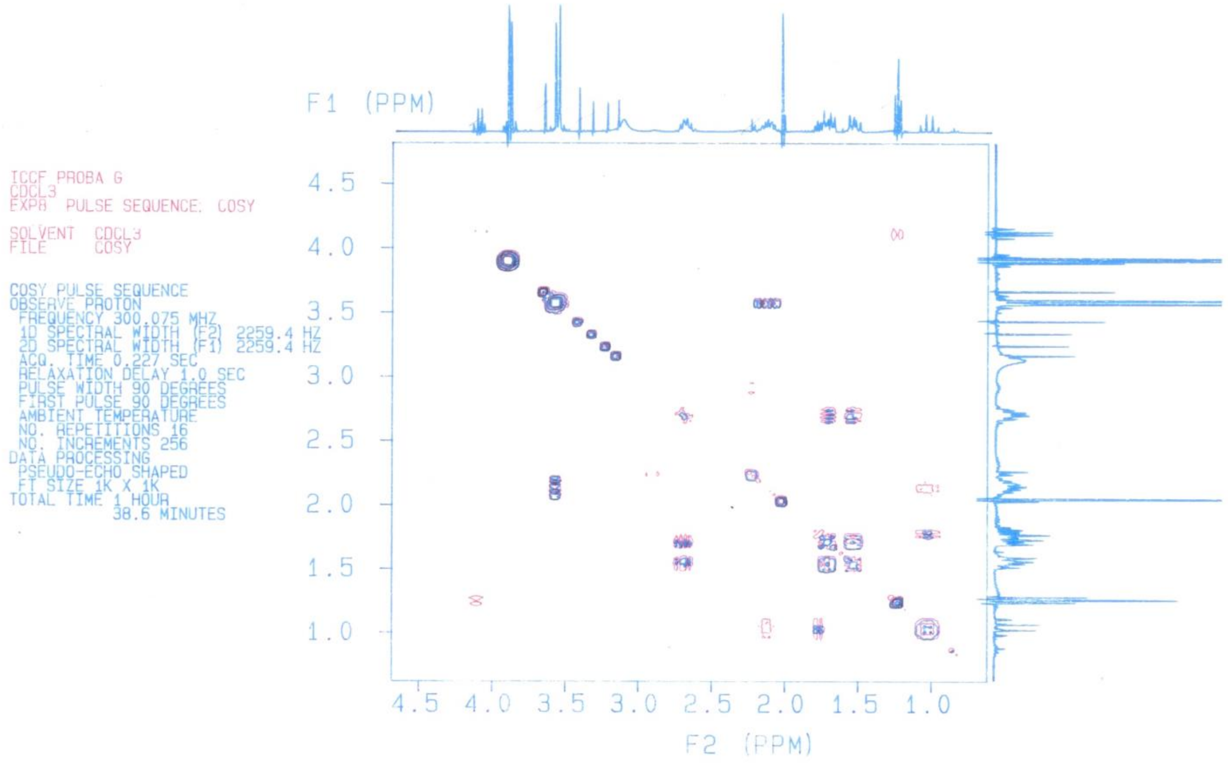
ICCF PROBA G
CDCL3
EXP8 PULSE SEQUENCE: COSY
SOLVENT CDCL3
FILE COSY
COSY PULSE SEQUENCE
OBSERVE PROTON
FREQUENCY 300.075 MHZ
1D SPECTRAL WIDTH (F2) 2259.4 HZ
2D SPECTRAL WIDTH (F1) 2259.4 HZ
ACQ. TIME 0.227 SEC
RELAXATION DELAY 1.0 SEC
PULSE WIDTH 90 DEGREES
FIRST PULSE 90 DEGREES
AMBIENT TEMPERATURE
NO. REPETITIONS 16
NO. INCREMENTS 256
DATA PROCESSING
PSEUDO-ECHO SHAPED
FT SIZE 1K X 1K
TOTAL TIME 1 HOUR
38.6 MINUTES
F1 (PPM)
F2 (PPM)

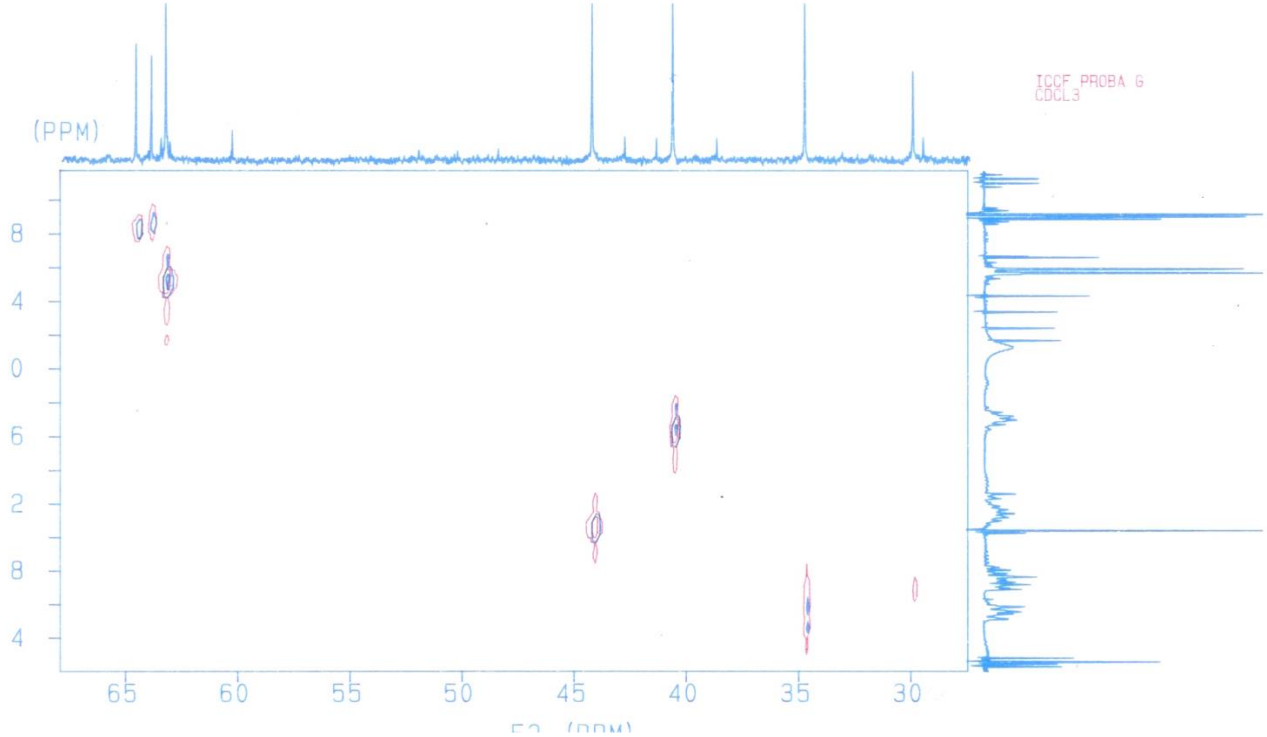
ICCF PROBA G
CDCL3
(PPM)

1.14. ^{1}H, ^{13}C, COSY and HETCOR spectra in DMSO of compound **15**

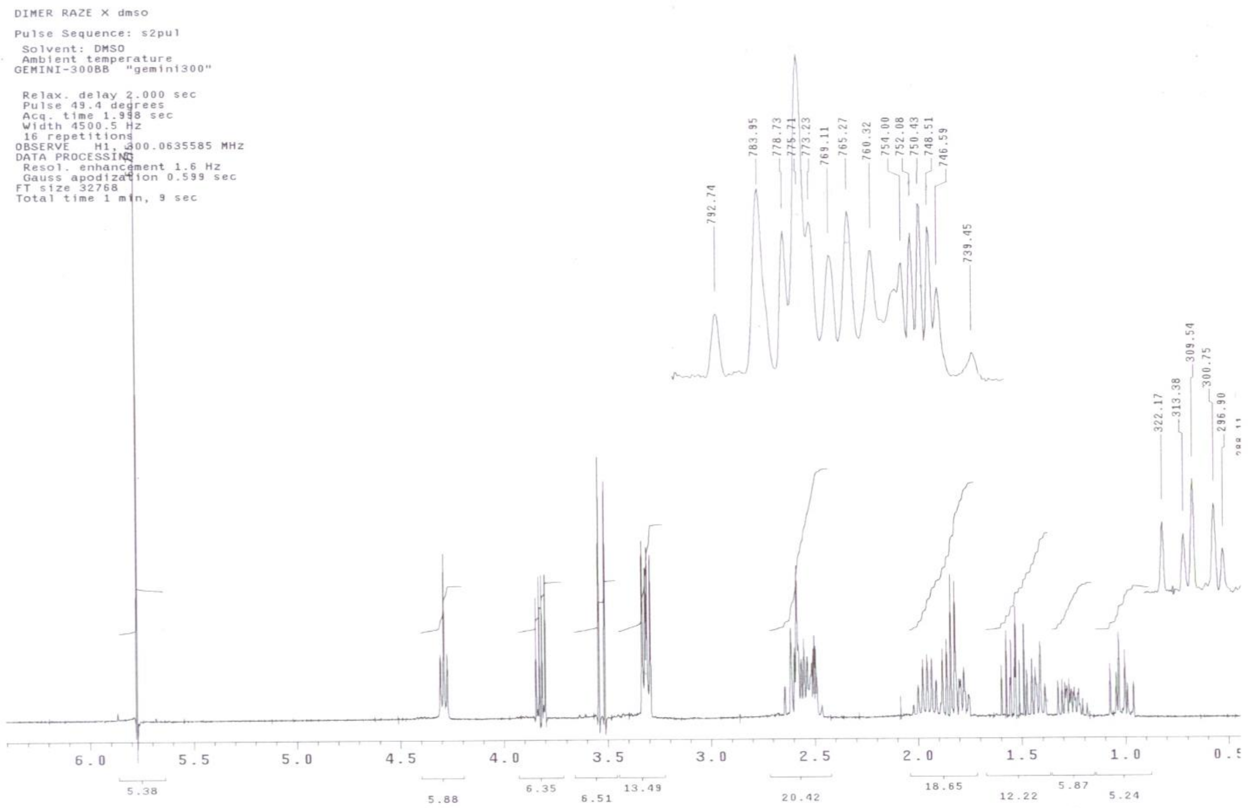

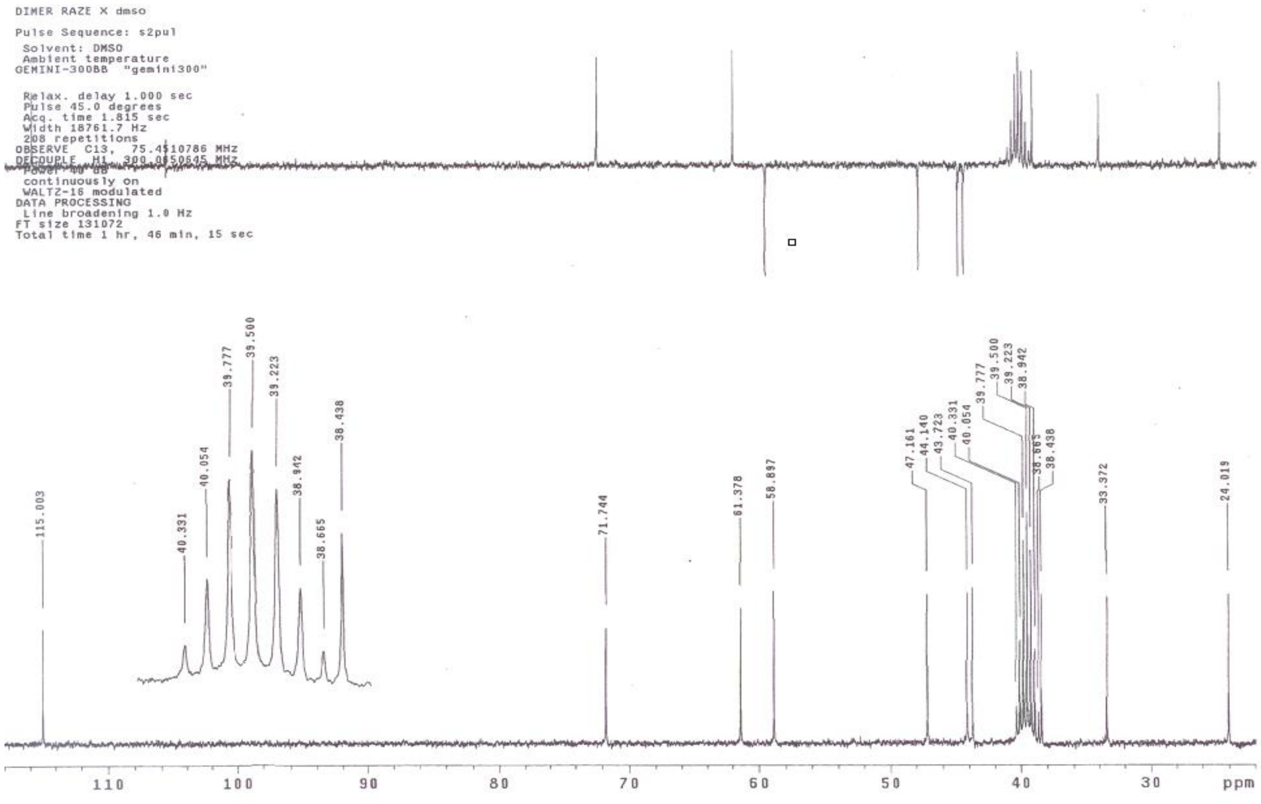

DIMER RAZE X dmso

Pulse Sequence: relayh

Solvent: DMSO
Ambient temperature
GEMINI-300BB "gemini300"

Relax. delay 1.000 sec
COSY 90-90
Acq. time 0.232 sec
Width 1104.7 Hz
2D Width 1104.7 Hz
4 repetitions
128 increments
OBSERVE H1, 300.0635585 MHz
DATA PROCESSING
Sine bell 0.116 sec
F1 DATA PROCESSING
Sine bell 0.058 sec
FT size 512 x 512
Total time 11 min, 55 sec

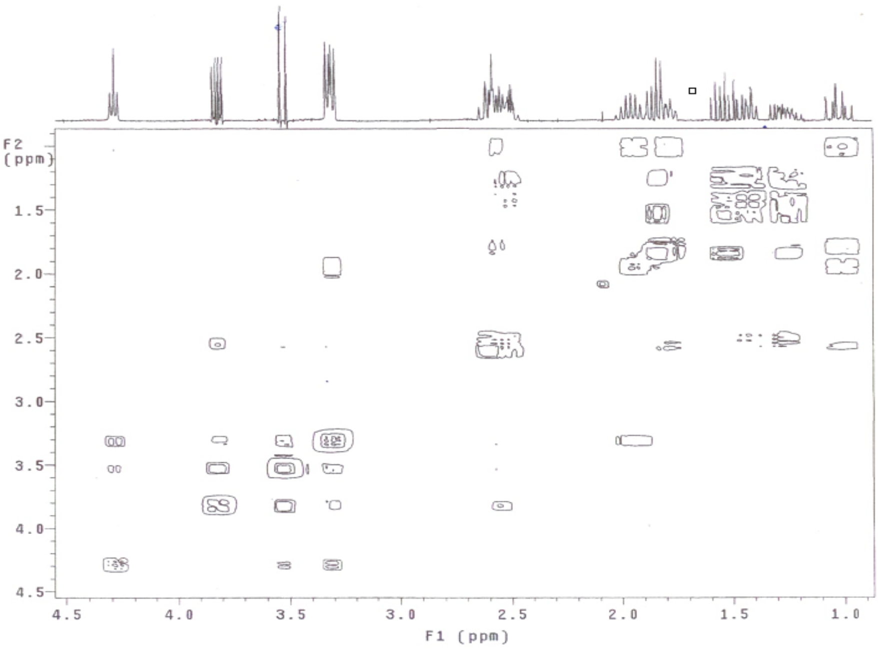

DIMER RAZE X dmso

Pulse Sequence: hetcor

Solvent: DMSO
Ambient temperature
GEMINI-300BB "gemini300"

Relax. delay 1.000 sec
Acq. time 0.066 sec
Width 3902.4 Hz
2D Width 1093.6 Hz
256 repetitions
64 increments
OBSERVE C13, 75.4510786 MHz
DECOUPLE H1, 300.0643459 MHz
Power 40 dB
on during acquisition
off during delay
WALTZ-16 modulated
DATA PROCESSING
Line broadening 1.0 Hz
F1 DATA PROCESSING
Line broadening 0.3 Hz
FT size 512 x 256
Total time 5 hr, 16 min, 54 sec

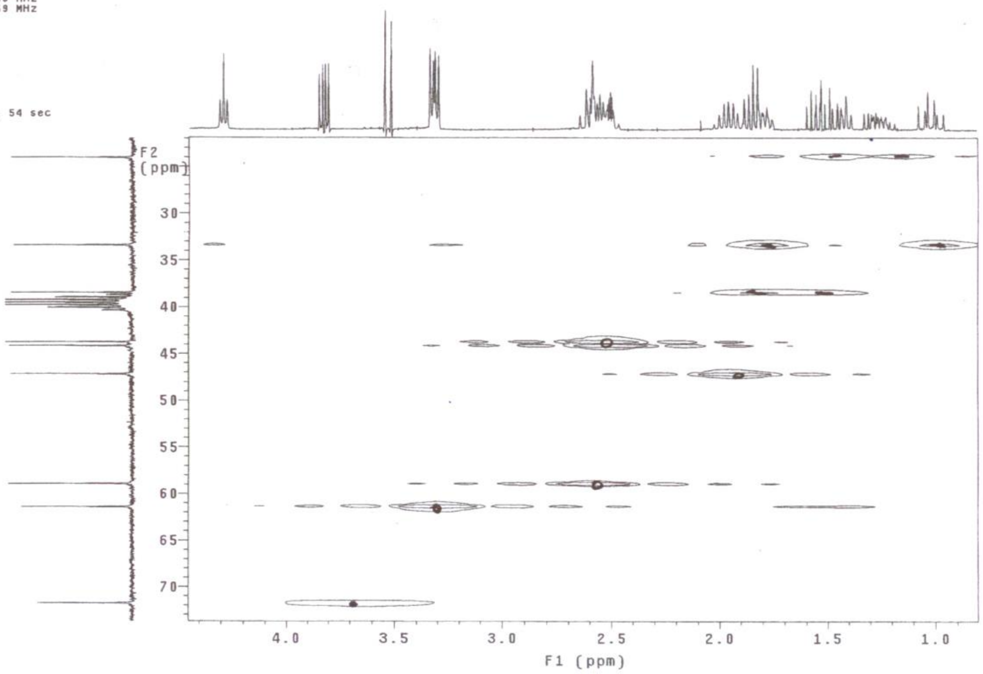

1.15. ^{1}H, ^{13}C, COSY and HETCOR spectra in $CDCl_3$ of the hemiketal compound **15**

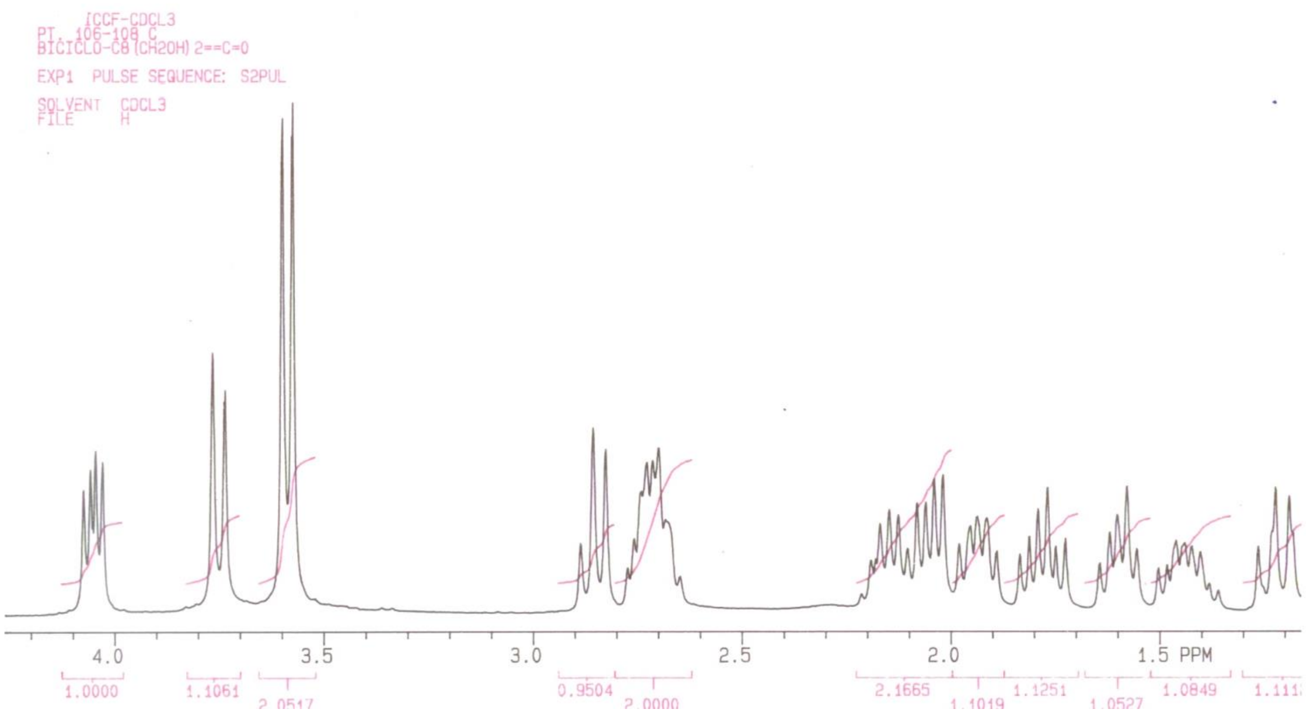

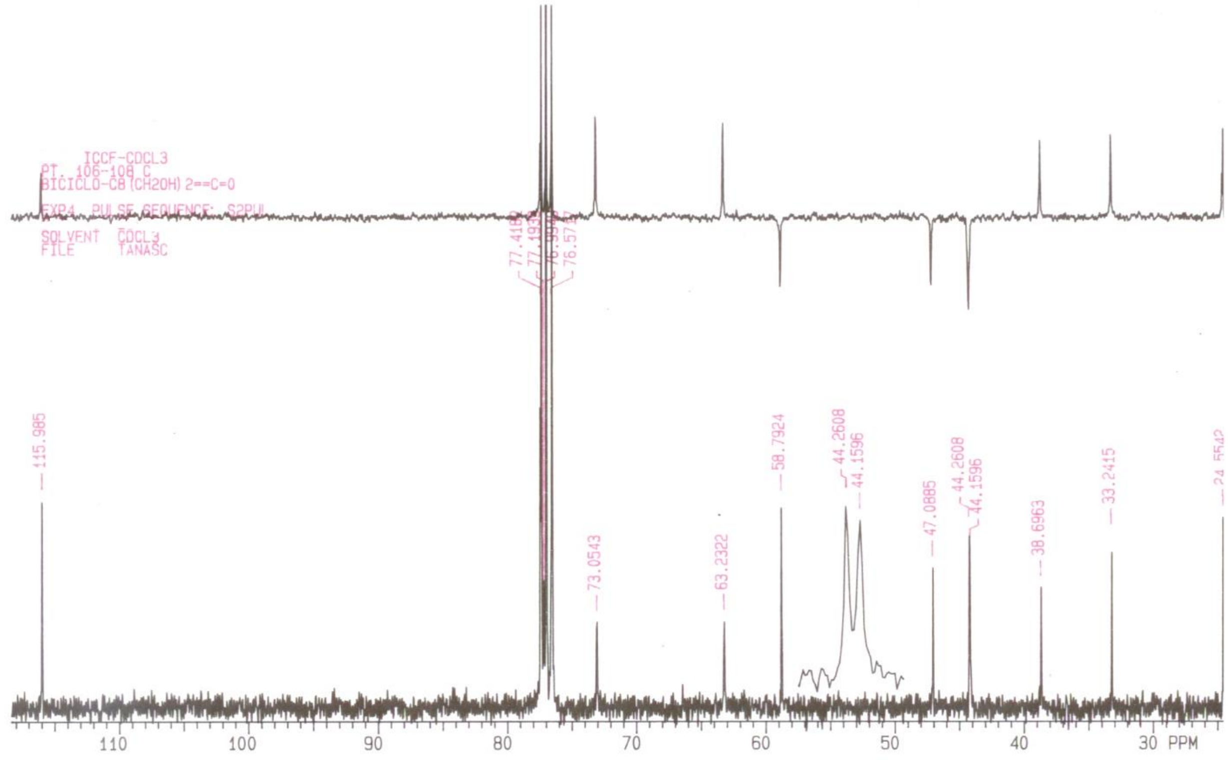

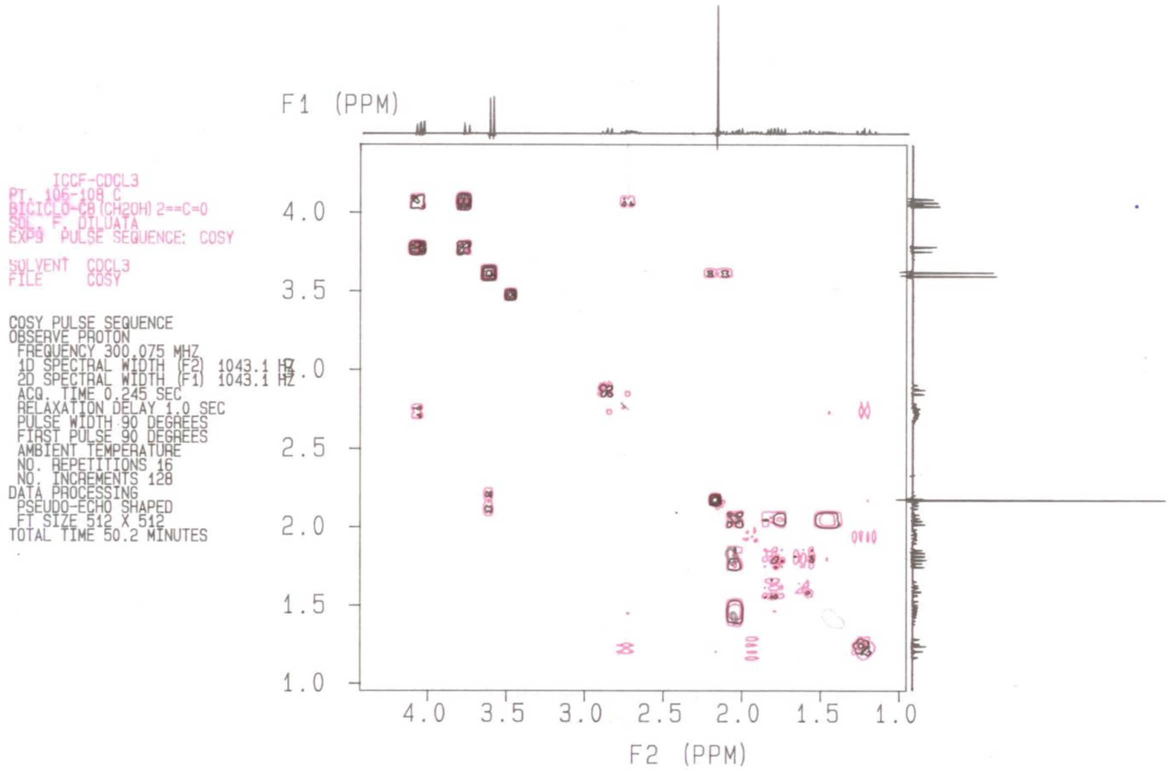
ICCF-CDCL3
PT. 106-108 C
BICICLO-C8 (CH2OH) 2==C=0
SOL. F. DILUATA
EXP9 PULSE SEQUENCE: COSY
SOLVENT CDCL3
FILE COSY
COSY PULSE SEQUENCE
OBSERVE PROTON
FREQUENCY 300.075 MHZ
1D SPECTRAL WIDTH (F2) 1043.1 HZ
2D SPECTRAL WIDTH (F1) 1043.1 HZ
ACQ. TIME 0.245 SEC
RELAXATION DELAY 1.0 SEC
PULSE WIDTH 90 DEGREES
FIRST PULSE 90 DEGREES
AMBIENT TEMPERATURE
NO. REPETITIONS 16
NO. INCREMENTS 128
DATA PROCESSING
PSEUDO-ECHO SHAPED
FT SIZE 512 X 512
TOTAL TIME 50.2 MINUTES
F1 (PPM)
F2 (PPM)

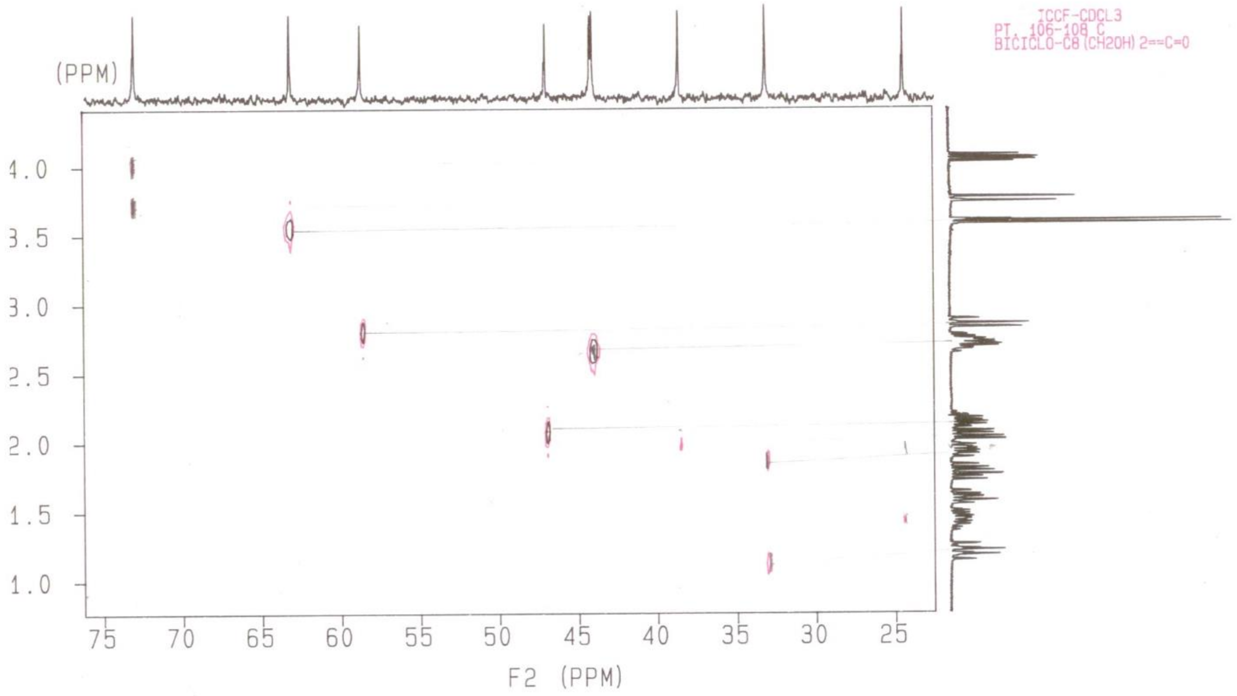
ICCF-CDCL3
PT. 106-108 C
BICICLO-C8 (CH2OH) 2==C=0
(PPM)
F2 (PPM)

2. X-ray crystallography of compounds 3, 5, 8 and 15: Table S1 and Table S2

Table S1. Bond distances (Å) and angles (°).

Compound **8**

O1-C7	1.424(2)	C1-C6A	1.557(3)
O2-C5	1.435(2)	C1-C8	1.509(3)
O3-C8	1.421(2)	C6a-C3a	1.566(3)
C7-C3	1.510(3)	C6A-C6	1.537(3)
C3-C2	1.528(3)	C3A-C4	1.534(3)
C3-C3a	1.541(2)	C4-C5	1.511(3)
C2-C1	1.527(3)	C5-C6	1.522(3)
O1-C7-C3	108.96(16)	C6-C6a-C3a	105.20(16)
C7-C3-C2	114.72(16)	C3-C3a-C6a	105.72(14)
C7-C3-C3a	115.84(16)	C4-C3a-C3	114.80(15)
C2-C3-C3a	103.19(15)	C4-C3a-C6a	104.44(16)
C1-C2-C3	103.14(15)	C5-C4-C3a	104.71(17)
C2-C1-C6a	104.01(15)	O2-C5-C4	110.68(17)
C8-C1-C2	113.43(16)	O2-C5-C6	110.10(18)
C8-C1-C6a	117.95(16)	C4-C5-C6	102.07(17)
C1-C6A-C3a	105.16(15)	C5-C6-C6a	105.69(16)
C6-C6A-C1	117.08(17)	O3-C8-C1	110.16(17)

Compound **3**.

O1-C4	1.450(3)	C10-C11	1.388(3)
O1-C16	1.340(3)	C10-C15	1.384(4)
O2-C16	1.208(3)	C11-C12	1.378(4)
O3-C7	1.450(3)	C12-C13	1.369(4)
O3-C23	1.331(3)	C13-C14	1.377(4)
O4-C23	1.195(3)	C14-C15	1.377(4)
O5-C8	1.448(3)	C16-C17	1.483(3)
O5-C9	1.339(3)	C17-C18	1.381(4)
O6-C9	1.205(3)	C17-C22	1.388(4)
C3-C2	1.533(3)	C18-C19	1.382(4)
C3-C3a	1.541(4)	C19-C20	1.372(5)
C3-C7	1.510(4)	C20-C21	1.374(5)
C1-C2	1.523(4)	C21-C22	1.376(4)
C1-C6a	1.536(4)	C23-C24	1.494(4)
C1-C8	1.504(3)	C24-C25	1.385(4)
C3A-C4	1.530(4)	C24-C29	1.376(4)
C3A-C6a	1.561(3)	C25-C26	1.384(4)
C4-C5	1.519(4)	C26-C27	1.362(5)
C5-C6	1.515(4)	C27-C28	1.370(5)
C6-C6a	1.541(4)	C28-C29	1.386(4)
C9-C10	1.473(4)		
C16-O1-C4	117.94(19)	C15-C10-C11	119.1(3)
C23-O3-C7	116.9(2)	C12-C11-C10	120.2(3)
C9-O5-C8	115.4(2)	C13-C12-C11	120.2(3)
C2-C3-C3a	103.7(2)	C12-C13-C14	120.1(3)

C7-C3-C2	114.0(2)	C13-C14-C15	120.2(3)
C7-C3-C3a	117.2(2)	C14-C15-C10	120.2(3)
C2-C1-C6a	103.6(2)	O1-C16-C17	111.9(2)
C8-C1-C2	115.6(2)	O2-C16-O1	123.0(2)
C8-C1-C6a	113.6(2)	O2-C16-C17	125.1(3)
C1-C2-C3	102.2(2)	C18-C17-C16	118.9(3)
C3-C3a-C6a	105.3(2)	C18-C17-C22	118.8(3)
C4-C3a-C3	116.7(2)	C22-C17-C16	122.3(3)
C4-C3a-C6a	105.2(2)	C17-C18-C19	120.6(3)
O1-C4-C3a	107.59(18)	C20-C19-C18	119.8(3)
O1-C4-C5	113.6(2)	C19-C20-C21	120.3(3)
C5-C4-C3a	105.3(2)	C20-C21-C22	120.0(3)
C6-C5-C4	101.7(2)	C21-C22-C17	120.5(3)
C5-C6-C6a	104.6(2)	O3-C23-C24	112.9(2)
C1-C6a-C3a	105.5(2)	O4-C23-O3	122.8(3)
C1-C6a-C6	116.4(2)	O4-C23-C24	124.3(3)
C6-C6a-C3a	104.7(2)	C25-C24-C23	117.6(3)
O3-C7-C3	108.1(2)	C29-C24-C23	123.0(3)
O5-C8-C1	107.7(2)	C29-C24-C25	119.4(3)
O5-C9-C10	112.4(2)	C24-C25-C26	120.1(3)
O6-C9-O5	123.0(3)	C27-C26-C25	120.2(3)
O6-C9-C10	124.6(2)	C26-C27-C28	120.1(3)
C11-C10-C9	118.2(3)	C27-C28-C29	120.4(3)
C15-C10-C9	122.6(2)	C24-C29-C28	119.9(3)

Compound **5**.

O1-C7	1.454(2)	C5-C6	1.495(3)
O1-C16	1.331(2)	C6-C6a	1.528(3)
O2-C16	1.200(2)	C9-C10	1.486(3)
O3-C5	1.213(3)	C10-C11	1.388(3)
O4-C8	1.449(2)	C10-C15	1.391(3)
O4-C9	1.337(2)	C11-C12	1.380(3)
O5-C9	1.206(2)	C12-C13	1.375(3)
C1-C2	1.522(3)	C13-C14	1.386(3)
C1-C6a	1.530(3)	C14-C15	1.379(3)
C1-C8	1.506(3)	C16-C17	1.483(3)
C2-C3	1.542(3)	C17-C18	1.385(3)
C3-C3a	1.557(3)	C17-C22	1.384(3)
C3-C7	1.503(3)	C18-C19	1.382(3)
C3A-C4	1.531(3)	C19-C20	1.374(3)
C3A-C6a	1.559(3)	C20-C21	1.378(3)
C4-C5	1.509(3)	C21C22	1.380(3)
C16-O1-C7	115.89(15)	O4-C9-C10	112.64(17)
C9-O4-C8	116.22(15)	O5-C9-O4	123.38(19)
C2-C1-C6a	103.33(16)	O5-C9-C10	123.97(19)
C8-C1-C2	110.54(16)	C11-C10-C9	121.69(19)
C8-C1-C6a	117.09(17)	C11-C10-C15	119.95(19)
C1-C2-C3	106.08(16)	C15-C10-C9	118.35(18)

C2-C3-C3a	105.99(16)	C12-C11-C10	119.8(2)
C7-C3-C2	108.81(17)	C13-C12-C11	120.2(2)
C7-C3-C3a	116.89(18)	C12-C13-C14	120.2(2)
C3-C3A-C6a	105.25(16)	C15-C14-C13	120.1(2)
C4-C3A-C3	118.04(17)	C14-C15-C10	119.7(2)
C4-C3A-C6a	105.00(16)	O1-C16-C17	112.86(16)
C5-C4-C3a	106.07(17)	O2-C16-O1	122.8(2)
O3-C5-C4	125.1(2)	O2-C16-C17	124.3(2)
O3-C5-C6	125.6(2)	C18-C17-C16	119.26(19)
C6-C5-C4	109.28(18)	C22-C17-C16	121.35(19)
C5-C6-C6a	107.13(17)	C22-C17-C18	119.3(2)
C1-C6A-C3a	104.87(16)	C19-C18-C17	120.0(2)
C6-C6A-C1	115.34(16)	C20-C19-C18	120.4(2)
C6-C6A-C3a	104.55(16)	C19-C20-C21	119.9(2)
O1-C7-C3	108.80(16)	C20-C21-C22	120.0(2)
O4-C8-C1	109.29(15)	C21-C22-C17	120.4(2

Compound **15**

	Molecule **A**	Molecule **B**
O1-C4	1.433(2)	1.440(2)
O1-C7	1.446(2)	1.451(2)
O2-C4	1.407(2)	1.397(2)
O3-C8	1.426(2)	1.428(2)
C1-C2	1.528(3)	1.530(2)
C1-C6a	1.538(2)	1.541(2)
C1-C8	1.519(3)	1.515(3)
C2-C3	1.534(2)	1.538(2)
C3-C3a	1.554(2)	1.550(2)
C3-C7	1.522(3)	1.529(2)
C3a-C4	1.531(2)	1.550(2)
C3a-C6a	1.549(2)	1.522(2)
C4-C5	1.520(2)	1.518(2)
C5-C6	1.532(3)	1.528(3)
C6-C6a	1.548(2)	1.545(2)
C4-O1-C7	106.97(13)	106.70(12)
C2-C1-C6a	103.13(14)	103.43(14)
C8-C1-C2	114.91(16)	113.18(15)
C8-C1-C6a	114.74(15)	115.41(15)
C1-C2-C3	105.42(14)	105.91(14)
C2-C3-C3a	105.08(14)	105.37(14)
C7-C3-C2	114.32(15)	114.00(15)
C7-C3-C3a	102.39(14)	102.88(14)
C4-C3a-C3	105.72(14)	105.72(14)
C4-C3a-C6a	106.43(13)	106.39(13)
C6a-C3a-C3	106.74(14)	106.90(14)
O1-C4-C3a	104.77(13)	104.36(13)
O1-C4-C5	108.39(14)	107.86(14)
O2-C4-O1	109.67(14)	110.43(14)

O2-C4-C3a	111.82(13)	111.40(14)
O2-C4-C5	115.13(14)	115.70(15)
C5-C4-C3a	106.48(15)	106.40(15)
C4-C5-C6	102.89(14)	103.19(14)
C5-C6-C6a	104.11(14)	104.22(14)
C1-C6a-C3a	104.60(14)	105.03(13)
C1-C6a-C6	115.21(15)	115.22(15)
C6-C6a-C3a	104.53(14)	104.71(14)
O1-C7-C3	104.20(14)	103.61(13)
O3-C8-C1	112.82(16)	111.35(16)

Table S2. Crystallographic data, details of data collection and structure refinement parameters.

	**8**	**3**	**5**	**15**
Chemical formula	$C_{20}H_{36}O_6$	$C_{31}H_{30}O_6$	$C_{24}H_{24}O_5$	$C_{20}H_{32}O_6$
M/g mol^{-1}	372.49	498.55	392.43	368.46
Temp./K	200	200	200	199.9(3)
Crystal system	orthorhombic	monoclinic	monoclinic	monoclinic
Space group	$Pna2_1$	$P2_1/c$	$P2_1/n$	$P2_1/n$
a/Å	12.0378(9)	17.3080(15)	10.6062(8)	14.3087(6)
b/Å	15.2016(13)	7.9623(9)	11.9338(9)	6.12783(17)
c/Å	5.2521(4)	18.879(2)	16.5623(12)	22.1843(7)
α/°	90.00	90.00	90.00	90.00
β/°	90.00	98.052(9)	103.467(7)	107.899(4)
γ/°	90.00	90.00	90.00	90.00
V/Å^3	961.10(13)	2576.1(5)	2038.7(3)	1851.00(11)
Z	2	4	4	4
D_c/g cm^{-3}	1.287	1.285	1.279	1.322
μ/mm^{-1}	0.093	0.089	0.089	0.096
Crystal size/mm^3	0.35 × 0.30 × 0.25	0.35 × 0.30 × 0.20	0.35 × 0.30 × 0.20	0.35 × 0.20× 0.05
θ_{min}, θ_{max}/°	4.32 to 50.06	4.36 to 50.04	4.16 to 50.04	5.64 to 50.06
Reflections collected	7419	10698	8488	27555
Independent reflections	1683 [R_{int} = 0.0378]	4559 [R_{int} = 0.0493]	3600 [R_{int} = 0.0309]	3279 [R_{int} = 0.0536]
Data/restraints/parameters	1683/1/119	4559/0/334	3600/0/262	3279/0/239
R_1^{a} [($I > 2\sigma(I)$)]	0.0382	0.0702	0.0559	1.054
wR_2^{b} (all data)	0.0880	0.1454	0.1199	0.0488
GOF^c	1.036	1.078	1.086	0.1208
Largest diff. peak and hole/e·Å^{-3}	0.14/-0.14	0.16/-0.20	0.24/-0.20	0.22/-0.23

^{a} $R_1 = \Sigma||F_o| - |F_c||/\Sigma|F_o|$, ^{b} $wR_2 = \{\Sigma[w(F_o^2 - F_c^2)^2]/\Sigma[w(F_o^2)^2]\}^{1/2}$. ^{c} GOF = $\{\Sigma[w(F_o^2 - F_c^2)^2]/(n - p)\}^{1/2}$, where n is the number of reflections and p is the total number of parameters refined.
